# DGAT1 activity synchronises with mitophagy to protect cells from metabolic rewiring by iron  depletion

**DOI:** 10.15252/embj.2021109390

**Published:** 2022-04-12

**Authors:** Maeve Long, Alvaro Sanchez‐Martinez, Marianna Longo, Fumi Suomi, Hans Stenlund, Annika I Johansson, Homa Ehsan, Veijo T Salo, Lambert Montava‐Garriga, Seyedehshima Naddafi, Elina Ikonen, Ian G Ganley, Alexander J Whitworth, Thomas G McWilliams

**Affiliations:** ^1^ Translational Stem Cell Biology & Metabolism Program, Research Programs Unit Faculty of Medicine Biomedicum Helsinki University of Helsinki Helsinki Finland; ^2^ MRC Mitochondrial Biology Unit University of Cambridge Cambridge UK; ^3^ MRC Protein Phosphorylation & Ubiquitylation Unit School of Life Sciences The Sir James Black Centre University of Dundee Dundee UK; ^4^ Swedish Metabolomics Centre Department of Plant Physiology Umeå University Umeå Sweden; ^5^ Department of Anatomy Faculty of Medicine Biomedicum Helsinki University of Helsinki Helsinki Finland; ^6^ Minerva Foundation Institute for Medical Research Helsinki Finland; ^7^ Present address: Science for Life Laboratory Department of Oncology and Pathology Karolinska Institutet Stockholm Sweden; ^8^ Present address: Structural and Computational Biology Unit European Molecular Biology Laboratory Heidelberg Germany; ^9^ Present address: Discovery Biology, Discovery Sciences R&D, AstraZeneca Cambridge UK

**Keywords:** DGAT1, iron, lipid droplet, metabolism, mitophagy, Autophagy & Cell Death, Metabolism

## Abstract

Mitophagy removes defective mitochondria via lysosomal elimination. Increased mitophagy coincides with metabolic reprogramming, yet it remains unknown whether mitophagy is a cause or consequence of such state changes. The signalling pathways that integrate with mitophagy to sustain cell and tissue integrity also remain poorly defined. We performed temporal metabolomics on mammalian cells treated with deferiprone, a therapeutic iron chelator that stimulates PINK1/PARKIN‐independent mitophagy. Iron depletion profoundly rewired the metabolome, hallmarked by remodelling of lipid metabolism within minutes of treatment. DGAT1‐dependent lipid droplet biosynthesis occurred several hours before mitochondrial clearance, with lipid droplets bordering mitochondria upon iron chelation. We demonstrate that DGAT1 inhibition restricts mitophagy *in vitro*, with impaired lysosomal homeostasis and cell viability. Importantly, genetic depletion of DGAT1 *in vivo* significantly impaired neuronal mitophagy and locomotor function in *Drosophila*. Our data define iron depletion as a potent signal that rapidly reshapes metabolism and establishes an unexpected synergy between lipid homeostasis and mitophagy that safeguards cell and tissue integrity.

## Graphical abstract

DGAT1‐dependent lipid homeostasis synergizes with PINK1/Parkin‐independent mitophagy to sustain cell and tissue integrity.

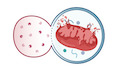

## Introduction

Mitochondrial dysfunction is classically linked to apoptotic cell death (Liu *et al*, 1996) and underscores a range of human disorders for which no disease‐modifying therapies exist, including neurodegeneration, myopathies and inflammaging (Gorman *et al*, 2016). Several mechanisms exist to neutralise mitochondrial dysfunction at different scales, from proteolytic degradation of individual proteins, targeted quality control by MDVs (mitochondrial‐derived vesicles) and selective elimination of the entire organelle by mitochondrial autophagy (termed mitophagy; McWilliams & Muqit, 2017; Palikaras *et al*, 2018; Suomi & McWilliams, 2019; Killackey *et al*, 2020; Long & McWilliams, 2020; Montava‐Garriga & Ganley, 2020; Singh & Ganley, 2021). Because defective mitophagy is predicted to promote tissue pathology, enhancement of mitochondrial turnover represents an attractive therapeutic strategy for several human diseases (Killackey *et al*, 2020). Mitophagy also plays a key role during tissue development and metabolic maturation (Palikaras *et al*, 2018; Rodger *et al*, 2018; Montava‐Garriga & Ganley, 2020). Yet, even in the presence of defective mitophagy, other signalling pathways likely underpin cell survival and integrity. How mitophagy integrates with different pathways to support metabolic integrity is not fully understood.

Increased mitophagy often coincides with metabolic transitions, and the most significant levels of mitophagy *in vivo* occur within tissues of high metabolic demand (Allen *et al*, 2013; Doménech *et al*, 2015; McWilliams *et al*, 2016, 2018a, 2018b, 2019; Esteban‐Martínez *et al*, 2017; Gkikas *et al*, 2018; Singh *et al*, 2020). However, the metabolic basis of mitochondrial turnover has not been studied in depth, and it is not clear if mitophagy is a cause or consequence of such metabolic transitions (Montava‐Garriga & Ganley, 2020). Most of our knowledge about mitophagy comes from the meticulous characterisation of the PINK1/Parkin signalling pathway, a ubiquitin‐dependent stress response activated by mitochondrial depolarisation in cultured cells (McWilliams & Muqit, 2017; Montava‐Garriga & Ganley, 2020). In healthy cells and tissues, steady‐state mitophagy can readily proceed in the absence of PINK1/Parkin signalling (Allen *et al*, 2013; Villa *et al*, 2017, 2018; Lee *et al*, 2018; McWilliams *et al*, 2018a; Yamada *et al*, 2018; Saito *et al,* 2019; Zachari *et al*, 2019; Zhang *et al*, 2019; Alsina *et al*, 2020; Singh *et al*, 2020). Mitophagy is also triggered by other stimuli including hypoxia, and ubiquitin‐independent turnover mechanisms also exist (Killackey *et al*, 2020; Montava‐Garriga & Ganley, 2020).

In recent years, a significant association between mitochondrial turnover and cellular iron homeostasis has transpired. This relationship is intriguing because iron metabolism represents a primordial function of the mitochondrial network, serving as a repository for an estimated 20–50% of cellular iron content (Richardson *et al*, 2010; McBride, 2018; Ward & Cloonan, 2019). Basal mitophagy may also provide an additional means to supplement lysosomes with iron, thereby facilitating functional acidity (Yambire *et al*, 2019; Weber *et al*, 2020). Therapeutic iron chelators such as deferiprone (DFP) are potent inducers of mitophagy (Allen *et al*, 2013; Hara *et al*, 2020; Zhao *et al*, 2020; Munson *et al*, 2021), and the therapeutic induction of mitophagy using clinically approved chelators may also prove more tractable than targeting endogenous mitophagy receptors (Rosignol *et al*, 2020). Iron depletion induces mitophagy independently of PINK1/Parkin (Allen *et al*, 2013) and involves induction of mitochondrial ferritin (Hara *et al*, 2020), yet several key questions remain. Aside from mitophagy, iron depletion induces respiratory chain deficiency (Oexle *et al*, 1999), glycolytic dependence (Allen *et al*, 2013) and lipid accumulation (Crooks *et al*, 2018) by an unknown mechanism. How or whether these events conspire to modulate mitophagy remains a mystery. Here, we establish the metabolic events leading to mitophagy induced by iron depletion, revealing surprisingly rapid and distinctive metabolic effects. Our findings unearth an unexpected yet critical synergy between DGAT1‐dependent lipid droplet biogenesis and mitophagy that safeguards cell and tissue integrity.

## Results

### Iron depletion rapidly reshapes the mammalian metabolome

Deferiprone (DFP) treatment eliminates approximately 50% of the mitochondrial network by PINK1/Parkin‐independent mitophagy (Allen *et al*, 2013). Compared with other therapeutic iron chelators, DFP is a more potent inducer of mitophagy because it is reported to preferentially chelate mitochondrial iron (Sohn *et al*, 2008; Hara *et al*, 2020). Several respirometry studies have reported that iron chelator treatment impairs mitochondrial oxidative phosphorylation (OXPHOS). These phenotypes are regarded to underpin a metabolic state transition whereby the loss of mitochondrial function accompanies the induction of HIF1‐alpha‐driven glycolytic dependence (Oexle *et al*, 1999; Allen *et al*, 2013; Hara *et al*, 2020; Zhao *et al*, 2020). Glycolytic metabolism appears necessary for mitophagy because DFP treatment does not promote mitophagy when cells are forced to use galactose as their primary carbon source (Allen *et al*, 2013).

Nonetheless, cultured proliferating cells have distinct energetic demands compared with tissues, where OXPHOS is essential for many vital organ systems (Vander Heiden *et al*, 2009). The ATP demands of cultured cells are satisfied by glycolysis rather than OXPHOS; hence, mitochondrial networks *in vitro* likely fulfil more anabolic than catabolic functions (Vander Heiden *et al*, 2009; Lunt & Vander Heiden, 2011; Young, 2013; Buescher *et al*, 2015). This raises several fundamental questions regarding the regulatory interplay between mitophagy and metabolism and the control of metabolism by iron homeostasis. For instance, most signalling studies investigating DFP‐induced mitophagy have focussed on timepoints corresponding to the peak of mitochondrial turnover (typically 24 h) (Allen *et al*, 2013; Hara *et al*, 2020; Zhao *et al*, 2020; Munson *et al*, 2021). Consequently, the metabolic events upon immediate iron depletion leading to mitophagy remain unexplored.

**Figure 1:**
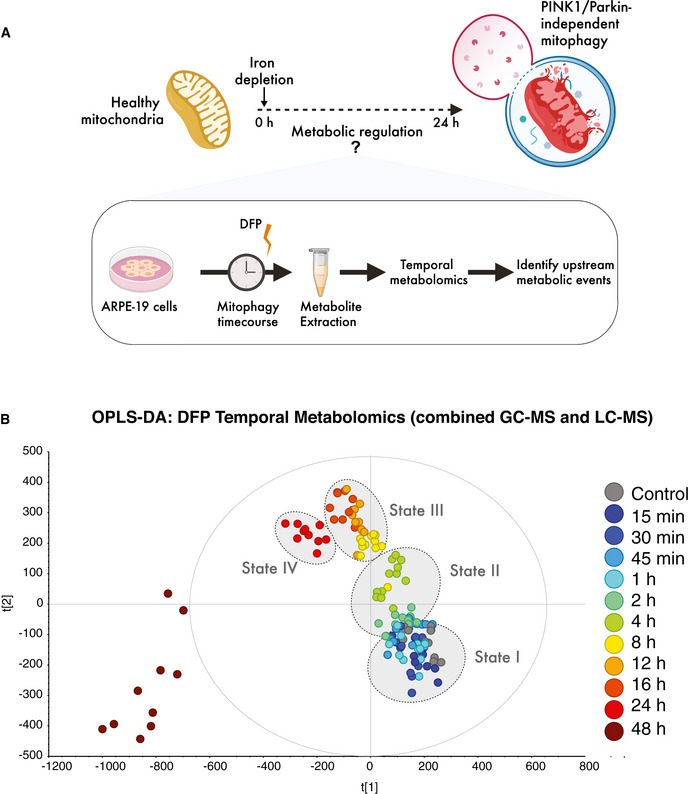
Temporal metabolomics reveals the mammalian metabolome is rapidly reshaped by iron depletion Schematic of temporal metabolomics workflow in human ARPE19 cells.Multivariate modelling of the metabolome reveals distinct transitions over time (states I–IV) in response to iron chelation using deferiprone (DFP). OPLS‐DA was computed and generated from combined LC‐MS and GC‐MS datasets from three independent biological experiments each with four technical replicates per timepoint in each experiment.

To define the metabolic events preceding mitophagy, we performed temporal metabolomics on human ARPE19 cells treated with DFP at a range of acute and extended time points from 15 min to 48 h (Fig 1A). We used LC‐MS and GC‐MS metabolomics to obtain broad coverage of the metabolome following DFP treatment. Multivariate modelling of combined temporal datasets enabled us to discriminate and classify four distinct metabolic states (I–IV) that transitioned and clustered as a function of iron depletion over time (Fig 1B). DFP treatment induced a striking and rapid shift in the metabolome, particularly between the 0–2 h, 2–4 h and 4–8 h clusters (Fig 1B). The cellular metabolome of 24 h DFP‐treated cells was entirely distinct from earlier time points. In contrast, we observed a very high degree of stochastic variation at 48 h of iron depletion, and samples from this extended treatment did not cluster in a stereotypical fashion; hence, we focussed on the 0–24 h timepoints for the remainder of the study (Fig 1B). As mitophagy peaks maximally from 24 h of DFP treatment, our data demonstrate that the metabolome of highly mitophagic cells is entirely distinct from cells with low levels of mitophagy when iron deprivation is the trigger.

### Lipid recomposition is acutely induced by iron depletion in mammalian cells

**Figure 2:**
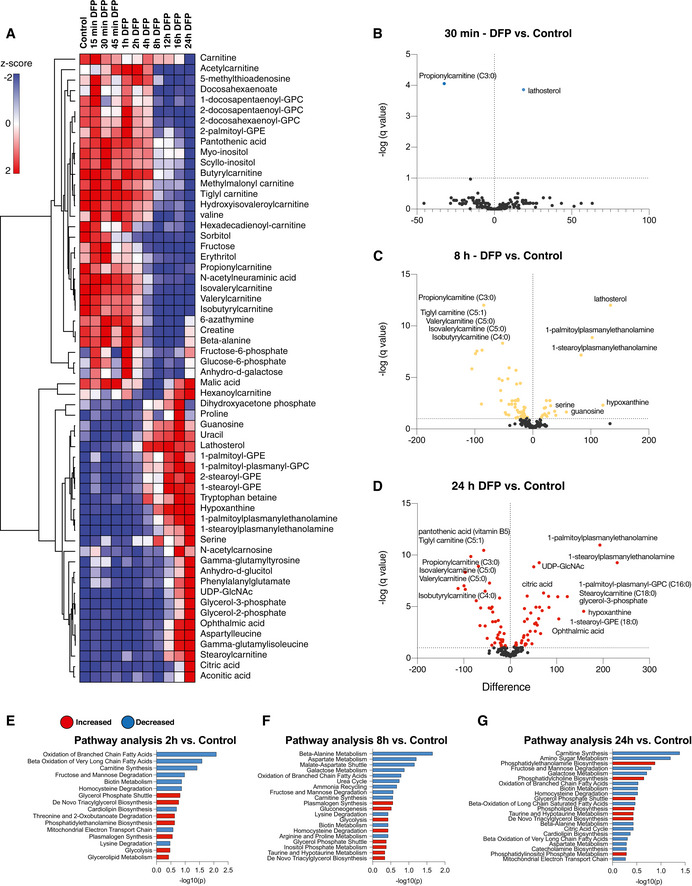
Metabolic rewiring by iron depletion is hallmarked by impaired lipid homeostasis AHierarchical clustering heatmap analysis of the 60 most altered metabolites in cells treated with DFP for the length of time indicated. Each coloured cell on the map corresponds to a *z*‐score value. The list was generated using MetaboAnalyst software and determined with an ANOVA test with a *P* < 0.05 threshold followed by *post‐hoc* analysis. The hierarchical clustering method of one minus Pearson correlation was applied (*n* = 3, with 4 technical replicates per biological replicate).B–DVolcano plots representing the difference in the mean of metabolites from DFP‐treated cells compared with control cells at acute (30 min), 8 h and overnight (24 h) timepoints, generated by unpaired *t*‐test with an FDR of 0.01. Coloured points represent significantly altered features.E–GPathway analysis generated in MetaboAnalyst using the significantly altered metabolites from the volcano plot comparisons. Statistical analysis was determined by hypergeometric test, and the library used for pathway analysis was SMPDB.

**Figure EV1:**
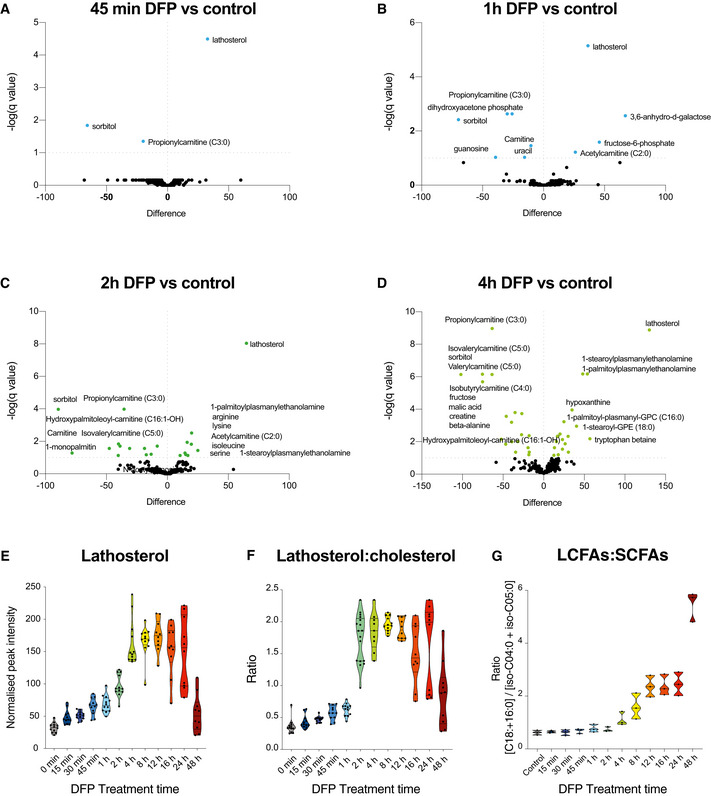
Additional volcano plots and ratios of affected metabolites upon loss of cellular iron A–DVolcano plots representing the difference in the mean of metabolites from DFP‐treated cells compared with control cells at acute (45 min) and at 1, 2 and 4 h timepoints, generated by unpaired T test with an FDR of 0.01. Coloured points are significantly altered.EGraph of lathosterol peak intensities over time (*n = *3 biological experiments with 4 technical replicates per timepoint in each experiment).FRatio of peak intensities of lathosterol over cholesterol upon DFP treatment over time. All data are derived from (*n = *3 biological experiments with 4 technical replicates per timepoint in each experiment).GRatio of peak intensities of long‐chain fatty acids (LCFA) over short‐chain fatty acids (SCFA) upon DFP treatment over time (*n = *3 biological experiments with 4 technical replicates per timepoint in each experiment).

**Figure EV2:**
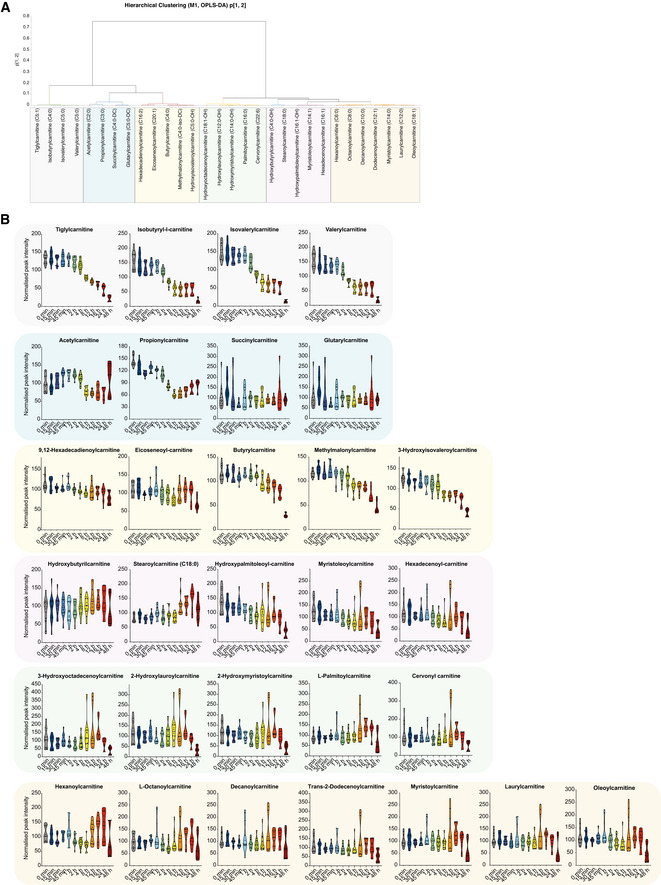
Iron depletion drives selective acylcarnitine dysregulation OPLS‐DA (5 + 1) model fitted for control and DFP samples for acylcarnitines (*N* = 136, *K* = 30). Hierarchical cluster analysis (HCA) for the p1 and p2‐loadings, calculated with Ward. Arbitrary threshold of 0.03 revealed seven sub‐groups for acylcarnitines.Graphs represent peak intensities of acylcarnitine metabolites over time revealing selective time‐dependent alterations upon DFP treatment. All graphs are represented as mean ± SEM, *n = *3 with 4 technical replicates per timepoint.

**Figure EV3:**
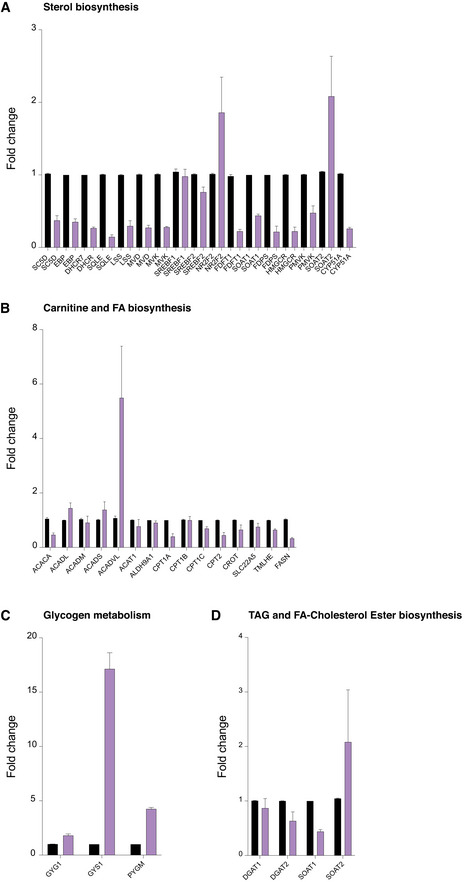
Gene expression analysis by RT‐qPCR upon iron depletion Gene expression analysis by TaqMan RT‐qPCR of mRNA transcripts encoding regulatory enzymes in carnitine and fatty acid biosynthesis in control and 24‐h DFP (1 mM)‐treated cells.Gene expression analysis by TaqMan RT‐qPCR of mRNA transcripts encoding regulatory enzymes in sterol biosynthesis in control and 24‐h DFP (1 mM)‐treated cells.Gene expression analysis by TaqMan RT‐qPCR of mRNA transcripts encoding regulatory enzymes in glycogen biosynthesis in control and 24‐h DFP (1 mM)‐treated cells.Gene expression analysis by TaqMan RT‐qPCR of mRNA transcripts encoding regulatory enzymes in triglyceride and fatty acid‐cholesterol ester biosynthesis in control and 24‐h DFP (1 mM)‐treated cells.

In agreement with the multivariate analysis of combined samples, hierarchical clustering of time points in the DFP treatment series revealed a profound metabolic shift in the metabolome at 8 h of iron depletion (Fig 2A). The acute metabolic response to iron depletion had several distinguishing features, hallmarked by rapid and selective alterations in lipid metabolism (Figs 2A–D and EV1). Acylcarnitines, sterols, plasmanylethanolamines and lysophosphatidylcholines (lyso‐PCs) were all differentially regulated upon iron chelation. We also detected substantially elevated hypoxanthine levels at 8 and 24 h (Figs 2A–D and EV1 and EV2), demonstrating a metabolic signature of pseudo‐hypoxia (Saugstad, 1988). Notably, iron depletion had highly selective effects on acylcarnitine and sterol metabolism within 15–30 mins of treatment (Fig 2B). Acute iron chelation specifically remodelled short‐chain acylcarnitines (SCACs) (Figs EV1 and EV2), reflected by a significant decrease in propionylcarnitine levels (C3:0) within 15 min of DFP treatment, which progressively decreased over time (Figs 2A–D and EV1 and EV2). Other SCACs were also diminished, including tiglylcarnitine, isobutyryl‐l‐carnitine, isovalerylcarnitine and valerylcarnitine (Figs EV1 and EV2). The only elevated long‐chain acylcarnitine species was stearoylcarnitine C18:0 (acylcarnitine C18:0) (Figs 2A–D and EV2A and B), whose elevation is also a feature of defective carnitine metabolism in patients (Minkler *et al,* 2005). An increased LCAC‐to‐SCAC ratio reflected the selective effects of iron depletion on fatty acid length throughout iron chelation (Fig EV1G). In contrast to decreased SCAC levels, we uncovered a reciprocal increase in lathosterol (a cholesterol precursor) at 30 min, with levels increasing progressively throughout iron depletion, suggesting a blockade in cholesterol biosynthesis (Fig 2B). The conversion of lathosterol to cholesterol constitutes a critical transition point in sterol biosynthesis (Krakowiak *et al*, 2003). Temporal analysis of the lathosterol‐to‐cholesterol ratio revealed a sustained elevation over 24 h (Fig EV1F). At later timepoints, we detected sustained changes in levels of lyso‐PC lipids, typically associated with infection and inflammation (Law *et al*, 2019), as well as increased plasmanylethanolamine levels (Figs 2C and D, and EV1D). Pathway analysis of significantly altered metabolites ranked lipid metabolic pathways as the most affected following short DFP exposure, with “decreased oxidation of branched fatty acids” and “increased *de novo* triacylglycerol biosynthesis” amongst the top 10 affected pathways (Fig 2E). In addition to the striking effects of iron depletion on lipid metabolism, “mitochondrial electron transport chain” was also decreased whilst “glycolysis” was increased upon DFP treatment (Fig 2E–G). Changes to lipid metabolism were also further verified by convergent gene expression signatures, measured by RT‐qPCR. Genes for carnitine synthesis, fatty acid metabolism, sterol biosynthesis and glycogen synthesis were all differentially affected by iron chelation (Fig EV3).

Our data reveal that even very brief iron depletion can trigger profound alterations in cellular lipid metabolism. These findings suggest DFP treatment may impair fatty acid metabolism with a corresponding reciprocal increase in TAG biosynthesis. These dramatic changes occur several hours in advance of mitophagy events induced by DFP treatment (mitochondrial damage sensing, priming, sequestration and selective clearance) (Zhao *et al,* 2020). Aberrant lipid signalling accompanies previously reported metabolic changes of dysfunctional mitochondrial Fe‐S biogenesis, impaired OXPHOS and induction of glycolysis at 24 h. These findings reveal an unexpected yet defined metabolic response to a therapeutic iron chelator and a unique metabolic signature for PINK1/Parkin‐independent mitophagy in mammalian cells.

### Lipid droplet biogenesis precedes the onset of mitophagy

**Figure 3:**
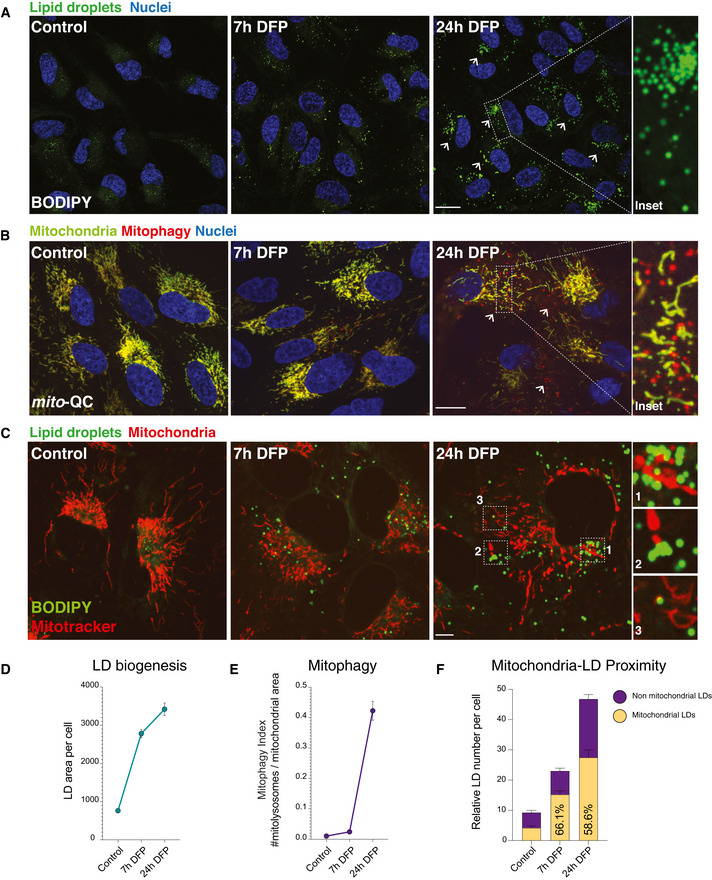
Lipid droplet biogenesis precedes the onset of mitophagy Lipid droplet time course. Representative photomicrographs showing lipid droplet abundance in ARPE19 cells treated with DFP starting at 7 h and maximal at 24 h. Neutral lipids were revealed by BODIPY 493/503. Nuclei are counterstained with Hoescht 33342. Inset shows detail of LDs in a cell treated with DFP for 24 h. Arrows highlight LD accumulation. Scale bars = 5 μm.Mitophagy time course. Representative photomicrographs showing time course of DFP treatment in mitophagy reporter cells (human ARPE19 cells with stable expression of *mito‐*QC). Mitochondrial networks are visible in yellow and mitolysosomes as red‐only puncta signifying mitophagy. Nuclei are counterstained with Hoescht 33342. Mitophagy appears maximal after 24 h of DFP treatment, with little difference between control and 7 h conditions. Inset shows details of mitolysosomes within the mitochondrial network of a highly mitophagic cell following DFP treatment. Arrows highlight mitolysosomes. Scale bar = 5 μm.Lipid droplets accumulate at mitochondria when cells are treated with DFP. Representative photomicrographs showing lipid droplet and mitochondria in ARPE19 cells treated with DFP at 7 h and at 24 h. Neutral lipids were revealed by BODIPY 493/503 and mitochondria by Mitotracker. Nuclei are counterstained with Hoescht 33342. Inset shows detail of LDs in close proximity to mitochondria in cells treated with DFP for 24 h. Scale bar = 5 μm.Quantification of lipid droplet area per cell over time. Lipid droplet area was quantified per cell area using a pipeline generated in CellProfiler. *n* = 3 with at least 50 cells quantified per biological replicate for each condition. Data represent means ± SEM.Quantification of mitophagy over time. Mitophagy index per cell is measured as a ratio of the number of mitolysosomes per mitochondrial content. *n* = 3 with at least 50 cells quantified per biological replicate for each condition. Data represent means ± SEM.Quantification of lipid droplet and mitochondria proximity over time. *n* = 3 with at least 50 cells quantified per biological replicate for each condition. Ratios between control and 24 h DFP; *P* < 0.05.

The earliest metabolic pathways affected by iron depletion were related to neutral lipid biosynthesis and fatty acid homeostasis. Consistent with our findings of DFP treatment on lipid metabolism, iron depletion by another therapeutic chelator (desferrioxamine—DFO) was previously shown to promote lipid droplet (LD) accumulation after 24 h by an unknown mechanism in stable cell lines lacking Fe‐S components (Crooks *et al*, 2018) and in rat renal tissues after prolonged DFP administration *in vivo* (Pereira *et al*, 2019). Yet, how alterations in lipid metabolism relate to mitophagy remains unexplored. Accordingly, we next defined the temporal dynamics between lipid droplet (LD) biogenesis and mitophagy. We treated wild‐type human ARPE19 cells with DFP at different timepoints and inspected LD formation using BODIPY 493/503 labelling. We observed a significant increase in individual LDs within 7 h of DFP treatment, followed by a substantial accumulation and aggregation of LDs at 24 h (Fig 3A and D, *P* < 0.0001). In parallel, we monitored mitophagy using wild‐type human ARPE19 cells expressing the well‐characterised reporter, *mito*‐QC (mCherry‐GFP‐FIS1^101–152^) (Allen *et al*, 2013; McWilliams *et al*, 2016, 2018a, 2018b, 2019; Lee *et al*, 2018; McWilliams & Ganley, 2019; Montava‐Garriga *et al*, 2020; Singh *et al*, 2020; Zhao *et al*, 2020). Under steady‐state conditions, all cytosolic mitochondria are visible by yellow fluorescence (red/green). When dysfunctional mitochondria are delivered to acidic endolysosomal compartments by selective autophagy, GFP fluorescence is quenched, but mCherry fluorescence remains stable. This strategy enables robust quantification of mitolysosome abundance, providing an “end‐point” readout of mitophagy levels in both cultured cells and tissues. Previous studies have demonstrated that DFP induces a significant peak of mitophagy activity after 18–24 h of treatment, with the first mitolysosomes visible between 8 and 16 h of treatment (Allen *et al*, 2013; Montava‐Garriga *et al*, 2020; Zhao *et al*, 2020). Our time‐course experiments using DFP verified this progression in mitophagy reporter cells, and mitolysosomes were most abundant at 24 h following iron depletion (Fig 3B and E, *P* < 0.0001). In contrast to the emergence of LDs at 7 h, mitolysosomes were not apparent at this earlier timepoint, reflecting differences in the dynamics and discrete stages of these processes. These results complement our temporal metabolomics data and demonstrate that iron depletion induces LD biogenesis prior to the encapsulation and delivery of damaged mitochondria to acidic endolysosomes.

LDs can readily associate with subpopulations of mitochondria in different physiological contexts (Wang *et al*, 2011; Rambold *et al*, 2014, 2015; Herms *et al*, 2015; Benador *et al*, 2018, 2019). We next assessed the spatial distribution of DFP‐induced LDs by measuring their proximity to the mitochondrial network upon iron depletion. We used confocal microscopy to quantify LD‐mitochondria proximity upon iron depletion (Fig 3C). These experiments revealed that at 7 and 24 h of DFP treatment, significantly more (60–65%) LDs bordered the mitochondrial network (Fig 3C and G; *P* < 0.001; Movies [Link], [Link], [Link]). These data demonstrate reciprocal effects of iron depletion on two distinct organelle populations, highlighting the emergence and accumulation of LDs against the backdrop of mitochondrial elimination, and demonstrate that iron depletion‐induced LD biogenesis occurs earlier than previously appreciated. Furthermore, LD formation precedes the clearance of damaged mitochondria and nascent LDs exhibit a propensity to cluster with the mitochondrial network before PINK1/Parkin‐independent elimination.

### Selective regulation of lipid droplet biogenesis by DGAT1 upon iron depletion

**Figure 4:**
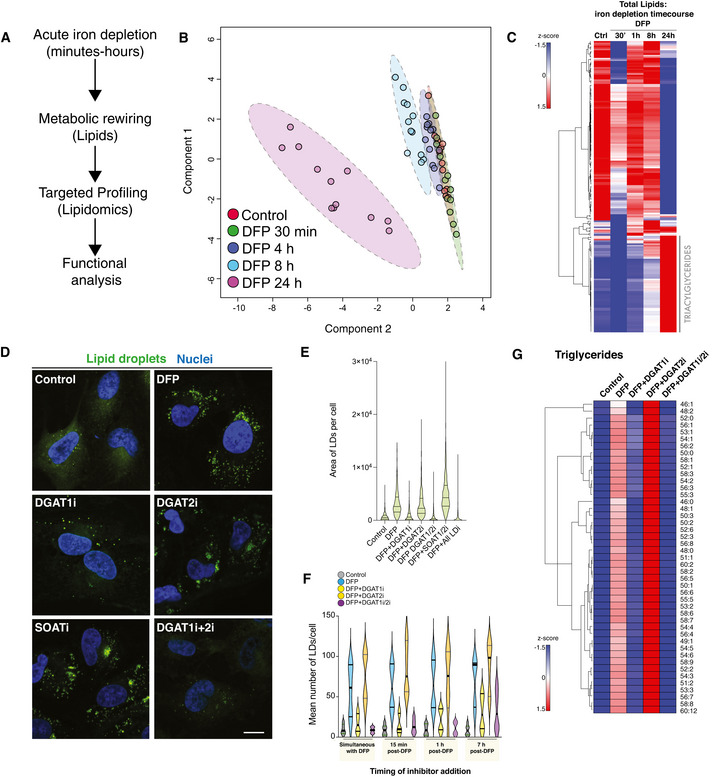
DGAT1 activation drives lipid droplet biogenesis upon iron depletion Line diagram of temporal lipidomics workflow in human ARPE19 cells.Multivariate modelling of the lipidome in cells treated with DFP over time. Sparse PLS‐DA (sPLS‐DA) was performed using Metaboanalyst from LC‐MS datasets from three independent biological experiments each with four technical replicates per timepoint in each experiment.Lipidomic analysis. Hierarchical clustering heatmap analysis showing alterations in lipid species in ARPE19 cells treated with DFP for the length of time indicated. Each coloured cell on the map corresponds to a *z*‐score value. The hierarchical clustering method of one minus Pearson correlation was applied (*n* = 3, with 4 technical replicates per biological replicate).DFP‐induced lipid droplet accumulation is dependent on DGAT1. Representative photomicrographs of ARPE19 cells treated with DFP (1 mM) for 7 h followed by treatment with LD inhibitors (5 μM) as indicated. Cells were fixed after 24 h of DFP treatment. Neutral lipid droplets were stained by BODIPY493/503, nuclei counterstaned with Hoescht and imaged by confocal microscopy after 24 h of DFP treatment (“I” denotes the presence of inhibitor, for example, DGAT1i = DGAT1 inhibition). Scale bar = 5 μm.Quantitation of lipid droplets area per cell from experiments in 2D. Data are represented as mean ± SEM (*n* = 3 with at least 80 cells analysed per condition in each biological replicate).Acute inhibition of LD biosynthesis by DGAT1. Quantitation of mean LD numbers per ± SEM (*n* = 2, with 3 technical replicates per biological experiment. Between 1,000 and 2,000 cells analysed per condition).DGAT1 regulates triglyceride formation upon iron chelation. Hierarchical clustering heatmap analysis showing alterations in triglyceride lipid species in ARPE19 cells treated with DFP for 24 h and lipid droplet inhibitors (5 μM) for 17 h as indicated (i denotes the presence of inhibitor, for example, DGAT1i = DGAT1 inhibition). Each coloured cell on the map corresponds to a *z*‐score value. The hierarchical clustering method of one minus Pearson correlation was applied, and statistical significance was determined by a *t*‐test (*n* = 3, with 5 technical replicates per biological replicate).

**Figure EV4:**
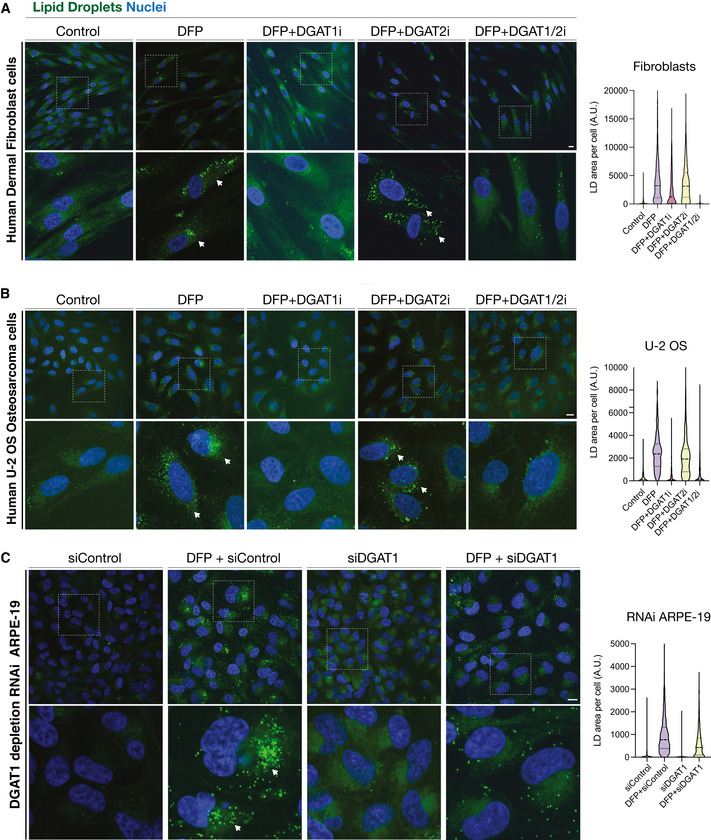
Iron depletion‐induced LD biogenesis occurs via DGAT1 in numerous cell subtypes Representative photomicrographs of human dermal fibroblasts treated for 24 h with DFP in the presence or absence of inhibitors to DGAT1 (DGAT1i), DGAT2 (DGAT2i) or DGAT1 and DGAT2 (DGAT1i/2i).Representative photomicrographs of human U2‐OS osteosarcoma cells, treated as in S5a.Representative photomicrographs demonstrating the effects of RNAi‐mediated DGAT1 depletion upon in human ARPE19 cells, upon 24 h DFP treatment (si = small interfering; siControl refers to scrambled or non‐targeting siRNA). Data information: In all experiments A–C, cells were stained with BODIPY to visualise lipid droplets and fixed. Nuclei were counterstained with Hoescht 33342. Scale bars = 5 μm. Quantitation accompanies each respective panel.

The mechanistic effector of LD biogenesis upon iron depletion remains unknown. LD biogenesis is complex and occurs in the endoplasmic reticulum (ER) bilayer, where fatty acids and cholesterol are converted to triacylglycerides (TAGs) and cholesterol esters (Walther *et al*, 2017; Thiam & Ikonen, 2021). Consistent with the induction of LD biogenesis, lipidomics profiling verified distinct lipid recomposition upon iron depletion (Fig 4A–C), revealing clear signatures of triacylglycerol (TAG) accumulation at 8 h and 24 h (Fig 4C). The final step in TAG biosynthesis involves the esterification of diacylglycerol (DAG) by diacylglycerol transferase enzymes (DGAT1 and DGAT2). DGAT1 is an ER‐resident enzyme, whereas DGAT2 activity is associated with ER subdomains, LDs and mitochondria (Stone *et al*, 2009). Combined inhibition of DGAT1 and DGAT2 impair LD biogenesis in a range of cell types. Thus, we next investigated the contribution of these enzymes to LD biogenesis upon iron depletion. We used DFP to induce LD biogenesis for 7 h before acute treatment with well‐characterised inhibitors of DGAT1 and DGAT2 (Nguyen *et al*, 2017; Salo *et al*, 2019) for 17 h, followed by confocal microscopy analysis (Fig 4D and E). DGAT1 inhibition abolished LD biogenesis, whereas this was not impaired by DGAT2 inhibition (Fig 4D and E, *P* < 0.0001). Combined treatment using DGAT1 and DGAT2 inhibitors suppressed LD biogenesis upon iron depletion (Fig 4D and E, *P* < 0.0001). Conversely, inhibition of sterol‐*O*‐acyltransferases 1 and 2 (SOAT1/2; enzymes responsible for cholesterol ester formation) did not inhibit LD biogenesis in DFP‐treated cells (Fig 4E, *P* > 0.05). Further experiments revealed that simultaneous addition of DFP and DGAT1/2 inhibitors, or very acute inhibition (15‐min, 30‐min or 1‐h post‐DFP) suppressed LD biogenesis comparable to 7‐h treatment (Fig 4F, *P* < 0.0001). Intriguingly, cells treated with DFP and DGAT2 inhibitors showed increased LD accumulation above DFP alone. Lipidomics profiling further verified that DFP‐induced TAG accumulation was selectively abolished by DGAT1i and DGAT1i/2i treatment, but not DGAT2i alone (Fig 4G). DFP treatment had a minimal effect on *DGAT1* or *DGAT2* mRNA transcripts (Fig EV3D). Importantly, iron depletion triggered robust LD biogenesis in several distinct cell types (human fibroblasts and U2‐OS cells), which was significantly and selectively arrested by treatment with DGAT1 inhibitors or upon combined DGAT1/2 inhibition (Fig EV4A and B, *P* < 0.0001). These findings were further phenocopied by siRNA‐mediated depletion of DGAT1 in human ARPE19 cells (Fig EV4C, *P* < 0.0001). Interestingly, other ubiquitin‐dependent and independent mitophagy stimuli did not provoke LD biogenesis to the same extent as iron depletion (Appendix Fig S1). Taken together, these results confirm that lipid rewiring induced by iron depletion is mediated by a DGAT1‐dependent mechanism that esterifies fatty acids to TAGs and ensures their packaging to LDs.

### Iron depletion‐induced LD biosynthesis occurs in the absence of autophagy signalling

**Figure 5:**
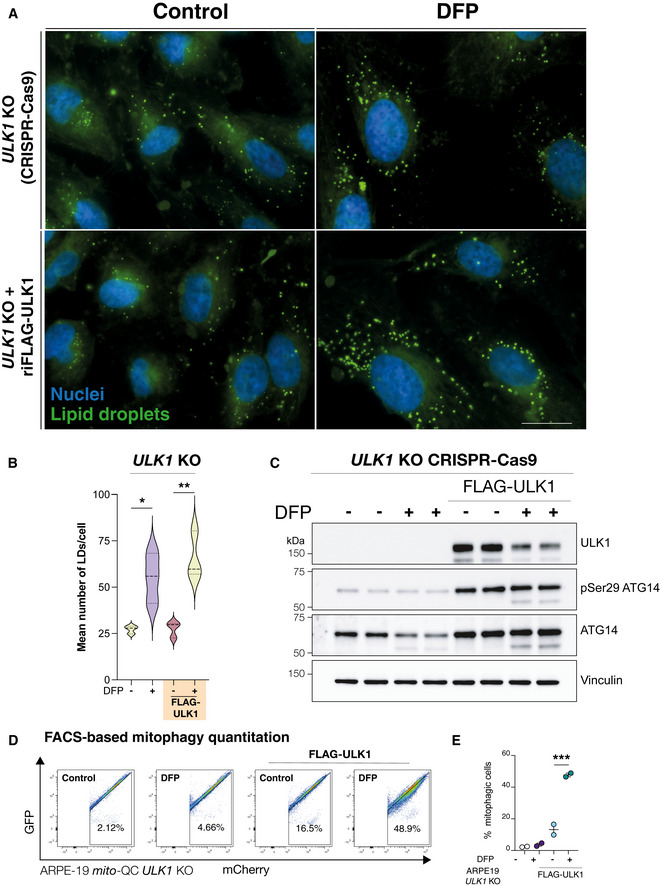
LD biogenesis by iron depletion is autophagy‐independent ARPE19 *ULK1* CRISPR KO cells with/without re‐introduction of FLAG‐ULK1 were treated with DFP for 24 h as indicated. Cells were fixed and stained with BODIPY493/503 to visualise lipid droplets (green) or DAPI to visualise nuclei (blue). Scale bar, 10 μm.Quantification from A, *n* = 3 independent experiments, statistical analysis by one‐way ANOVA. *P < 0.05; **P ≤ 0.01. Median represented by dashed line, quartiles by dotted lines.Authentication of cell lines in A by immunoblot of protein lysates.Representative flow cytometry assay of *mito*‐QC ARPE19 *ULK1* CRISPR KO cells with/without re‐introduction of FLAG‐ULK1, treated with DFP for 24 h. A decrease/increase in GFP/mCherry expression was quantified to measure the per cent of cells undergoing mitophagy (indicated by number in each panel).Quantitation of the flow data representing mean of *n* = 2 ± SEM. Statistical analysis was performed with one‐way ANOVA and a Tukey’s multiple comparisons test. ***P < 0.001.

The relationship between LD biosynthesis and non‐selective macroautophagy is well established. Nutrient depletion or mTORC1 inhibition induces macroautophagy, which liberates fatty acids that are then channelled into nascent LDs for storage (Olzmann & Carvalho, 2019). Both genetic and pharmacological impairments in autophagic flux inhibit LD biogenesis (*Atg5* KO MEFs or bafilomycin A1, respectively) (Rambold *et al,* 2015; Nguyen *et al,* 2017). Conversely, nothing is known about iron depletion‐induced LD biosynthesis and the requirement for autophagy. To mechanistically dissociate DFP‐induced LD biosynthesis from autophagy signalling, we treated *ULK1* KO cells generated by CRISPR‐Cas9 genome editing with DFP and monitored LD biogenesis. The ULK1 complex is a master regulator of autophagy initiation, integrating and transducing multiple upstream signals to promote autophagosome biogenesis (Zachari & Ganley, 2017). Furthermore, ULK1 is also reported to translocate to mitochondria during PINK1/Parkin‐independent mitophagy (Egan *et al*, 2011; Liu *et al*, 2012; Tian *et al*, 2015). Iron chelation induced significant levels of LD biogenesis and accumulation in *ULK1* KO cells (Fig 5A and B; *P* < 0.05). Furthermore, levels of LD biosynthesis were not noticeably altered by re‐complementation of *ULK1* KO cells with wild‐type FLAG‐ULK1 (Fig 5A and B; *P* < 0.001). Biochemical verification confirmed the absence of ULK1 and autophagy signalling in these cells, as well as restoration of DFP‐induced selective autophagy signalling upon re‐complementation with FLAG‐ULK1 (Fig 5C). To verify that DFP could not induce mitophagy in these cells, we also quantified mitophagy in ARPE19 *ULK1* KO cells with stable expression of *mito*‐QC. FACS‐based analysis further demonstrated minimal mitophagy in the absence of endogenous ULK1, which was successfully reversed upon the re‐complementation of FLAG‐ULK1 in KO cells (Fig 5D and E, *P* < 0.001). Together these data establish that iron chelation‐induced LD biogenesis occurs independently of selective autophagy.

### DGAT1 inhibition constrains mitophagy and disrupts endolysosomal homeostasis

**Figure 6:**
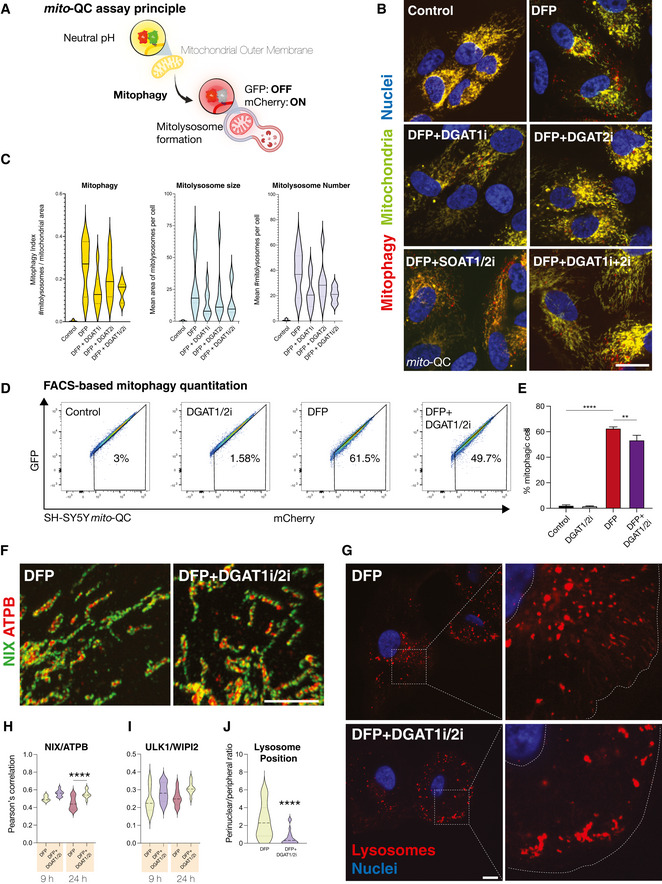
Reduced DFP‐induced mitolysosomes upon DGAT1 inhibition Schematic outlining the *mito*‐QC mitophagy reporter system. mCherry‐GFP is targeted to the mitochondrial outer membrane via the specific targeting sequence of FIS1. Cytosolic mitochondrial network appears as yellow, due to red–green fluorescence. Upon mitophagy, mitochondria delivered to endolysosomes are distinguished by mCherry‐only puncta, whereas GFP does not fluoresce in the acidic microenvironment.Representative photomicrographs of mitophagy reporter cells (human ARPE19 cells with stable expression of *mito‐*QC) treated with DFP (1 mM) for 7 h followed by treatment with LD inhibitors (5 μM) as indicated on the images (I denotes the presence of inhibitor, for example, DGAT1i = DGAT1 inhibition). Cells were fixed after 24 h of DFP treatment. Mitochondrial networks are visible in yellow and mitolysosomes as red‐only puncta signifying mitophagy. Nuclei are counterstained with Hoescht 33342. Scale bar = 5 μm.Mitophagy quantification for experiments in 5a. Mitophagy index per cell is measured as a ratio of the number of mitolysosomes per mitochondrial content. Graph generated using the mean of each biological replicate ± SEM (*n* = 7 with at least 70 cells quantified per biological replicate for each condition). Mean area of mitolysosomes per cell and mean number of mitolysosomes per cell for experiments in 5A.Representative flow cytometry assay of *mito*‐QC SH‐SY5Y cells treated with DMSO or LD inhibitors (DGAT1/2i, 5 μM) for 24 h, in the presence or absence of DFP. A decrease/increase in GFP/mCherry expression is quantified to measure the per cent of cells undergoing mitophagy (indicated by number in each panel).Combined quantitation of *mito*‐QC flow data is represented by mean percentage of cells undergoing mitophagy from three independent experiments ± SEM. Statistical analysis was performed with one‐way ANOVA and Tukey’s multiple comparisons test. ****P < 0.0001; **P < 0.01.Enlarged regions from representative confocal photomicrographs showing the pattern of endogenous mitochondrial NIX in human ARPE19 cells treated with DFP ±DGAT1i/2i. Samples were immunolabelled with primary antibodies to NIX (green) and ATPB (red). Scale bar = 5 μm.Representative confocal photomicrographs of cathepsin‐reactive endolysosomes in human ARPE19 cells treated with DFP ± DGAT1i/2i. Inset shows examples of lysosomal phenotypes. Scale bar = 5 μm.Colocalisation quantitation for experiments shown in 6g. One‐way ANOVA with Bonferroni’s *post‐hoc* test. ****P < 0.0001. Median represented by dashed line, quartiles by dotted lines *n* = 2 biological replicates.Colocalisation quantitation for ULK1/WIPI2 immunolabelling in human ARPE19 cells treated with DFP ± DGAT1i/2i. Median represented by dashed line, quartiles by dotted lines *n* = 2 biological replicates.Quantitation of lysosome positioning in DFP ± DGAT1i/2i. One‐way ANOVA with Bonferroni’s *post‐hoc* test. ****P < 0.0001. Median represented by dashed line, quartiles by dotted lines *n* = 2 biological replicates.

**Figure EV5:**
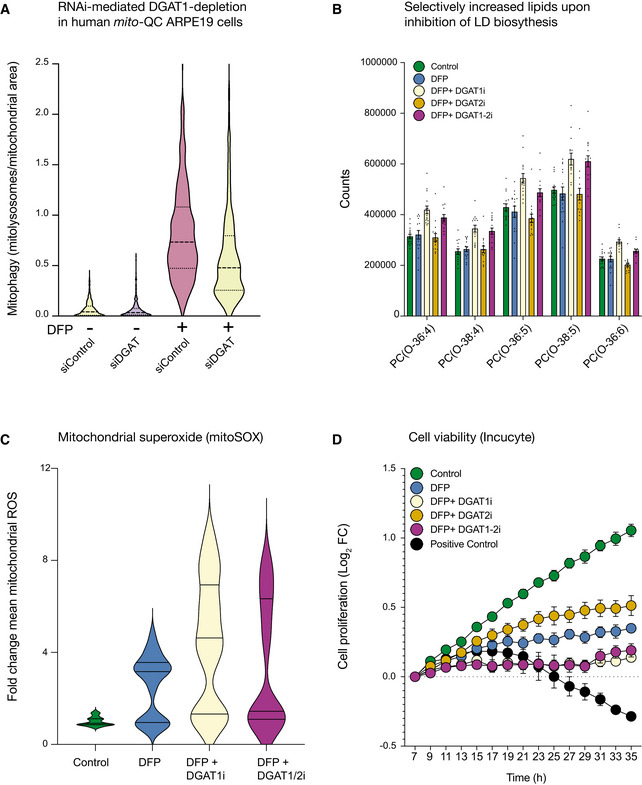
Impaired LD biogenesis promotes lipid dysfunction, oxidative stress and impaired viability upon iron depletion DFP‐induced mitophagy is significantly reduced upon RNAi‐mediated depletion of *DGAT1* in human ARPE19 *mito‐*QC cells (*n* = 3 experimental replicates).DGAT1 inhibition specifically increases ether‐linked phosphatidylcholines during iron chelation. Graph of the amount of specific lipid species measure in conditions indicated on figure (i denotes the presence of inhibitor, for example, DGAT1i = DGAT1 inhibition) and displayed as mean ± SEM (*n* = 3, with 4 technical replicates per biological replicate).Inhibition of DGAT1‐dependent LD biogenesis drives mtROS production. Cells treated with DFP (1 mM) for 24 h and LD inhibitors (5 μM) for 17 h were incubated with MitoSOX and imaged by spinning disc confocal imaging (i denotes the presence of inhibitor, for example, DGAT1i = DGAT1 inhibition). Graph of the mean fold change in fluorescence intensity ± SEM (*n* = 3 with at least 70 cells quantified per biological replicate for each condition).DGAT1 inhibition worsens the effect of iron chelation on cell proliferation. ARPE19 cells treated with DFP (1 mM) in combination with DGAT1 inhibitors (5 μM) ceased to proliferate in comparison with cells treated with DFP only. Positive control cells are treated with puromycin (7 μM). Results are presented as mean ± SEM of Log_2_ fold change, *n* = 3 experimental replicates; each experimental replicate is the average of technical duplicates.

The mobilisation of neutral lipids can support the progression of non‐selective macroautophagy in yeast and human cells (Dupont *et al*, 2014; Li *et al*, 2015; Shpilka *et al*, 2015; Ogasawara *et al*, 2020). Iron depletion induced DGAT1‐dependent formation of LDs, many of which clustered with the mitochondrial network upstream of mitophagy. Thus, we next examined the possible relationship between DGAT1‐dependent LD biogenesis and mitophagy. We treated *mito*‐QC ARPE19 reporter cells with DFP and monitored mitolysosome levels after inhibition of either DGAT1, DGAT2, SOAT1/2 or combined DGAT1/2 inhibition (Fig 6A and B). Predictably, DFP induced robust levels of mitochondrial turnover, evidenced by an abundance of mCherry‐only mitolysosomes after 24 h (Fig 6B and C, *P* < 0.0001). DFP also triggered mitophagy in the absence of LDs, yet the overall number of mitolysosomes was consistently reduced upon DGAT1 inhibition or combined DGAT1/2 inhibition (Fig 6B and C, *P* < 0.05). Consistent with our previous experiments on LDs, SOAT1/2 inhibition did not affect DFP‐induced mitophagy (Fig 6B). However, we observed lower levels of DFP‐induced mitophagy and a significant difference in the number and size of mitolysosomes upon DGAT1 inhibition (Fig 6C, *P* < 0.0001). We next verified these findings using siRNA‐mediated depletion of DGAT1 in DFP‐treated *mito*‐QC ARPE19 cells. These experiments phenocopied our inhibitor data, showing a significant decrease in mitophagy with siDGAT1 compared with control siRNA cells upon iron depletion (Fig EV5A, *P* < 0.0001). We further performed orthogonal verification of our findings using a distinct cell type and detection approach. In agreement with previous experiments, DGAT1/2 inhibition induced a modest yet consistent and significant reduction in DFP‐induced mitophagy in SH‐SY5Y human neuroblastoma cells with stable expression of *mito‐*QC, as revealed by high‐content FACS‐based quantitation Fig 6D and E, *P* < 0.001).

We next investigated the mechanism underlying decreased mitophagy levels upon DGAT1/2 inhibition. DFP induces mitochondrial accumulation of NIX/BNIP3L to engage ATG8 proteins and facilitate selective autophagy (Montava‐Garriga & Ganley, 2020). Although LDs manifested upstream of mitolysosomes, previous work demonstrates DFP increases NIX/BNIP3L levels from 4‐h post‐treatment, with robust stabilisation from 8 h (Zhao *et al*, 2020). We hypothesised that loss of LDs could interfere with sensing of mitochondrial damage or impair priming of the damaged organelle for elimination. Accordingly, we performed high‐resolution confocal imaging of endogenous NIX at 9‐h and 24‐h post‐DFP, in the presence or absence of combined DGAT1/2 inhibition. DFP treatment readily induced mitochondrial NIX accumulation compared to untreated control cells, with high‐resolution microscopy revealing endogenous NIX on damaged mitochondria (Fig 6F–H). Comparative analysis and quantification of confocal images revealed that the targeting and mitochondrial distribution of NIX was not impaired by the absence of LDs at 9 h (Fig 6F–H; *P *> 0.05; 9 h timepoint). Intriguingly, we detected a modest yet statistically significant increase in mitochondrial NIX at 24 h with DGAT1/2 inhibition, suggesting increased mitochondrial dysfunction upon inhibition of LD biogenesis (Fig 6H; *P *< 0.0001; 24 h timepoint). Similarly, we also investigated whether the endogenous autophagy machinery was affected upon DGAT1/2 inhibition during DFP‐induced mitophagy. DFP robustly induced the formation of both ULK1 and WIPI2 foci irrespective of DGAT1/2 inhibition (Fig 6I; *P *> 0.05; 9 h, 24 h timepoints), with no corresponding increase as observed for NIX at 24 h. These data demonstrate that impaired LD biogenesis does not impair damage sensing, priming or autophagic induction upon iron depletion.

Following damage sensing, priming and encapsulation of dysfunctional organelles, selective autophagy next requires a competent pool of acidic endolysosomes for efficient completion. Aberrant lipid metabolism compromises lysosomal activity and altered fatty acid dysfunction can compromise lysosomal membrane homeostasis and pH, which is also tightly connected to their subcellular location (Johnson *et al,* 2016). To this effect, lysosomal position is also a critical determinant of starvation‐induced macroautophagy (Korolchuk *et al,* 2011). Accordingly, we next assessed lysosomal activity and localisation by labelling acidic endolysosomes with the cathepsin‐substrate dye, Magic Red (MR). Although the overall intensity of MR labelling per cell did not differ, these experiments revealed differences in the distribution of cathepsin‐reactive endolysosomes upon DGAT1/2 inhibition (Fig 6G). Cathepsin‐positive endolysosomes were consistently displaced towards the cell periphery in the absence of LDs (Fig 6J; *P* < 0.01) suggesting aberrant localisation and or dynamics. Although lysosomal displacement is often accompanied by alkalinisation, we did not see a consistent or sustained elevation in lysosomal pH upon DGAT1/2 depletion, which we speculate may be a consequence of brief treatment times or experimental duration. Despite reduced mitolysosome abundance in DFP+DGAT1/2 conditions, displacement phenotypes were not immediately apparent in mitophagy reporter cells, suggesting a subset of lysosomes may be preferentially affected by LD biogenesis. Regardless, our data demonstrate reduced levels of DFP‐induced mitophagy upon loss of DGAT1 activity, accompanied by selective impairments in lysosomal homeostasis. Consistent with the protective role of LDs in cellular homeostasis (Nguyen *et al,* 2017), DGAT1/2 inhibition also promoted the enrichment of lipid species (*e.g*. phosphatidylcholines and ceramides) associated with metabolic and membrane dysfunction (Donovan *et al*, 2013; Yeon *et al*, 2017; Stamenkovic *et al*, 2021) (Fig EV5B, Appendix Fig S2), exacerbated mtROS levels (Fig EV5C, *P* < 0.01) oxidative stress metabolism (ophthalmic acid; Fig 2A–D) and compounded the effects of iron depletion on cell proliferation (Fig EV5D, *P* < 0.01) (Soga *et al*, 2006; Servillo *et al*, 2018). Collectively, these findings demonstrate adaptive DGAT1 activity safeguards cellular integrity, by counteracting abnormal lipid metabolism induced by iron depletion to facilitate efficient mitochondrial elimination and cell survival.

### DGAT1 depletion impairs physiological mitophagy *in vivo* and impairs neural function

**Figure 7:**
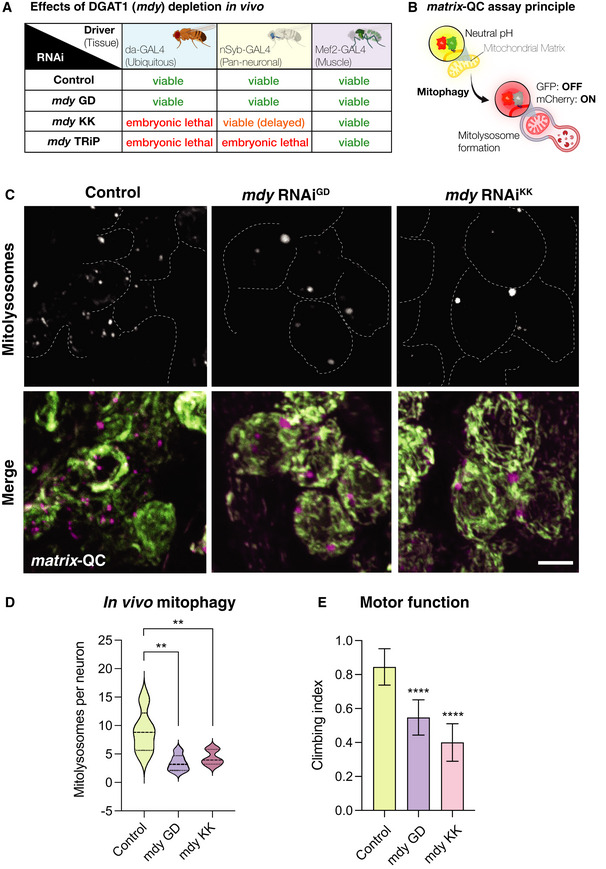
Phenotypic effects of DGAT1 (*mdy*) depletion on mitophagy *in vivo* Phenotypic assessment of DGAT1/*mdy* knockdown by different RNAi transgenes by GAL4 drivers ubiquitously or in a tissue‐specific manner.Schematic for the *matrix*‐QC mitophagy reporter system. mCherry‐GFP is targeted to the mitochondrial matrix via the specific COXVIII targeting sequence. As for *mito*‐QC, the cytosolic mitochondrial network appears in yellow, due to red–green fluorescence. Upon mitophagy, mitochondria delivered to endolysosomes are distinguished by mCherry‐only puncta, whereas GFP does not fluoresce in the acidic microenvironment.Representative photomicrographs of larval neuronal cell bodies expressing the *matrix*‐QC reporter and RNAi for *mdy* or control driven with nSyb‐GAL4. GFP is shown in green, mCherry is shown in magenta. Mitolysosomes (mCherry‐only puncta) are shown in greyscale. Scale bar = 5 μm.Quantitative analysis of mitophagy (mitolysosome per neuronal cell body). Data are shown as violin plot with median (dashed line) and quartile range (dotted lines); *n* = 5–6 animals, with 23–40 cells per animal. One‐way ANOVA with Šidák’s post‐test correction for multiple samples; **P < 0.01.Analysis of locomotor behaviour (climbing) in animals with neuron‐specific (nSyb‐GAL4) knockdown of *mdy* or control. Bars show mean ± 95% CI; *n* = 40, 57 and 45 animals, respectively. Kruskal–Wallis test with Dunn’s *post‐hoc* correction; ****P < 0.0001.

To authenticate the physiological significance of the unexpected link between lipid homeostasis and mitophagy, we next performed genetic experiments to investigate the contribution of endogenous DGAT1 to mitophagy in tissues. In conventional mammalian cell culture systems, mitophagy levels are generally low without pharmacological or genetic induction. However, studies using optical reporter animals have demonstrated that mitophagy is widespread *in vivo* and regulated in a cell and tissue‐specific fashion (McWilliams *et al*, 2016, 2018a, 2018b, 2019; Singh *et al*, 2020). Basal mitophagy is conserved from mice to flies (Lee *et al*, 2018). High levels of basal mitophagy are observed in several tissues, and owing to their high degree of tractability, we studied the effect of DGAT1 depletion in mitophagy reporter flies. *DGAT1* has a single orthologue in *Drosophila* termed midway (*mdy*), which also regulates LD biogenesis and homeostasis *in vivo* (Van Den Brink *et al*, 2018; Lubojemska *et al,* 2021). We targeted *mdy* knockdown using three independent and previously characterised inducible RNAi transgenes in combination with ubiquitous (all tissues: da‐GAL4) or tissue‐specific drivers (pan‐neuronal: nSyb‐GAL4; muscle: Mef2‐GAL4) (Van Den Brink *et al*, 2018; Martínez *et al*, 2020; Lubojemska *et al,* 2021). Our initial characterisation of DGAT1/*mdy* knockdown revealed an important role in development, consistent with the critical metabolic role of DGAT1/*mdy*, with different tissues and different RNAi lines displaying differing severities (Fig 7A).

Our mitophagy experiments in human cells took advantage of the *mito*‐QC reporter, localised to the OMM. For our *in vivo* mitophagy characterisation, we utilised a variant of the mCherry‐GFP double tag reporter, localised to the mitochondrial matrix using the targeting sequence of COXVIII (termed “*matrix*‐QC”). Like *mito*‐QC, *matrix*‐QC exploits the acid‐labile properties of GFP within the acidic endolysosome to provide an end‐point readout of mitophagy events as mCherry‐only puncta (Fig 7B). Because mitophagy in the fly brain has been previously characterised and exhibits abundant mitolysosomes (Lee *et al*, 2018), we induced pan‐neuronal depletion of DGAT1/*mdy* in *matrix*‐QC reporter animals, using two distinct RNAi lines. Confocal analysis and quantitation revealed a striking and highly significant reduction in neuronal mitophagy in both DGAT1/*mdy* knockdown conditions (Fig 7C and D, *P* < 0.01). Mitolysosomes in DGAT1‐deficient animals were less abundant but also visibly altered in profile compared with their control counterparts. Some DGAT1/*mdy* knockdown animals also exhibited wing posture defects, akin to those observed with *pink1* and *parkin* mutant flies (Fernandes & Rao, 2011). We next characterised motor function upon loss of DGAT/*mdy* using the negative geotaxis (climbing) assay which provides a sensitive readout of locomotor ability in flies. Neuron‐specific DGAT1/*mdy* depletion induced striking motor impairments, compromising climbing behaviour in flies (Fig 7E, *P* < 0.0001). Taken together, these phenotypes verify and complement our *in vitro* work, demonstrating an important contribution of DGAT1 to physiological mitophagy and tissue integrity *in vivo*.

## Discussion

Our work establishes the metabolic events arising from iron depletion and reveals an unexpected synergy between mitophagy and DGAT1‐dependent LD formation. We observed surprisingly rapid temporal effects of iron depletion, which induces metabolic rewiring hallmarked by impaired lipid homeostasis. Previous studies reported LD accumulation after extended iron depletion (Crooks *et al*, 2018; Pereira *et al*, 2019), yet the mechanism driving LD biogenesis and its relationship to mitophagy were unknown. We identified DGAT1 as the functional effector of LD biogenesis upstream of mitophagy and authenticated functional crosstalk between DGAT1‐dependent LD biosynthesis and mitophagy using a variety of experimental approaches. Importantly, we observed a genetic phenotype that matched these *in vitro* findings using mitophagy reporter animals lacking DGAT1, verifying the physiological importance of our work.

A clear contrast between our results and the published literature on iron depletion is timing, as most studies have focused predominantly on signalling events following 18–24 h of chelation treatment (Oexle *et al*, 1999; Allen *et al*, 2013; Hara *et al*, 2020; Zhao *et al*, 2020; Munson *et al*, 2021). Specifically, temporal metabolomics revealed that iron depletion is a potent signal that induces alterations in cellular lipid recomposition within minutes. This unprecedented finding suggests that lipid dysfunction may foreshadow defective OXPHOS reported in previous studies (Oexle *et al*, 1999; Allen *et al*, 2013; Crooks *et al*, 2018; Hara *et al*, 2020; Zhao *et al*, 2020). Competition for iron has shaped evolutionary dynamics between primates and pathogens, and mitochondrial iron likely played a key role during eukaryogenesis (Barber & Elde, 2015; McBride, 2018). Iron is also vital for the endolysosomal network (Yambire *et al*, 2019; Weber *et al*, 2020). Further work on the dynamic relationship between lipid homeostasis and iron may reveal fundamental insights into the acquisition of membrane and metabolic integrity.

Metabolic signatures indicated iron depletion‐induced impairments on fatty acid oxidation, carnitine synthesis, cardiolipin biosynthesis and biotin metabolism, signifying mitochondrial dysfunction at acute timepoints. Control of acylcarnitine homeostasis is crucial for cellular health, and DFP has highly selective effects on carnitine handling. Impaired carnitine homeostasis promotes cytosolic acyl‐CoA accumulation, increasing DAG or ceramide, which can drive PKC activation or apoptosis, respectively (Huang *et al,* 1999; Gurr *et al,* 2016). Lipid remodelling was also evident at the endoplasmic reticulum, evidenced by disrupted sterol metabolism and increased TAG biosynthesis. Alterations in fatty acid and sterol metabolism at the early stages of DFP treatment may reflect metabolic signals that might eventually favour phagophore biogenesis, curvature and expansion, given that cholesterol depletion promotes autophagic signalling (Schütter *et al*, 2020; Cheng *et al*, 2006). Correspondingly, *de novo* TAG biosynthesis, glycolysis, glycerolipid metabolism and phospholipid biosynthesis were enriched pathways upon iron depletion. In addition, DFP treatment also increased plasmalogen synthesis, suggesting intriguing links between mitochondrial iron and peroxisomal homeostasis that merit future exploration. Interestingly, Andrejeva and colleagues recently linked altered plasmalogen profiles with autophagosome/autolysosome formation and maintenance (Andrejeva *et al*, 2020). Our current findings suggest that iron depletion‐induced mitochondrial dysfunction redirects glycolytic flux towards adaptive lipid biosynthesis to preserve organelle and cellular integrity (Bar‐Even *et al*, 2012; Chandel, 2021). Ultimately, iron depletion reshaped the metabolome by 8 h of treatment, with the early induction of *de novo* lipogenesis and DGAT1 activity required to esterify fatty acids to TAG for storage within LDs.

The modality of LD biogenesis by iron depletion does not depend upon autophagic signalling, as evidenced by genetic complementation studies in *ULK1* KO cells. However, LDs may stimulate autophagy in different contexts via channelling or sequestering lipid species that promote or prevent autophagosome formation, respectively (Dupont *et al*, 2014; Li *et al*, 2015; Shpilka *et al*, 2015; Velázquez *et al*, 2016). Accordingly, we examined the possibility of a synergistic relationship between DGAT1‐dependent LD biosynthesis and mitophagy upon iron depletion using three reporter strategies. Across distinct cell types, analysis of mitophagy reporter cells revealed a consistent reduction in mitolysosomes upon impaired LD biogenesis. Because we found that nascent LDs rapidly associate with the mitochondrial network upon iron depletion, we predicted this interplay might regulate the priming or “marking” of defective mitochondria for destruction by selective autophagy. DFP induces the accumulation of NIX/BNIP3L on damaged mitochondria, which serves as an “eat‐me” signal by conjugating the damaged organelle with ATG8 proteins. As DGAT1/2 inhibition did not affect mitochondrial NIX/BNIP3L accumulation or distribution, our current findings do not support a role for DFP‐induced LDs in “priming” mitochondria for autophagic elimination at least in this particular context.

The yeast orthologue *Dga1* influences autophagosome biogenesis during nitrogen starvation, and *dga1Δ* mutants harbour excess DAG at the ER, arresting macroautophagy (Li *et al*, 2020). However, DFP treatment readily induced WIPI2 and ULK1 foci irrespective of LD biogenesis. Thus, we next investigated if reduced mitolysosome abundance upon DGAT1 inhibition may reflect impaired or delayed autophagy initiation, impacting autophagosome assembly. Together, we conclude that DGAT1‐dependent LD biosynthesis does not likely influence the priming or encapsulation of damaged mitochondria in our *in vitro* experiments. We speculate the increased crosstalk of LDs with the mitochondrial network may facilitate metabolic plasticity upon iron depletion, as this interplay dramatically changes the functional capacity of mitochondrial subpopulations (*e.g*. peri‐droplet mitochondria in adipose tissues; (Benador *et al*, 2018; Benador *et al*, 2019). The functional significance of this association is currently unclear but may also facilitate neutralising elevated levels of cytotoxic mitochondrial superoxide observed upon loss of LD biosynthesis. Because priming and autophagy initiation were unaffected by DGAT1/2 inhibition, we next explored lysosomal homeostasis, which is critical for mitophagy completion. Notably, live‐cell imaging revealed a displacement of cathepsin‐reactive endolysosomes upon DGAT1/2 inhibition. The positioning of lysosomes is tightly coupled to their activity and degradative capacity (Johnson *et al*, 2016), and defective positioning impairs non‐selective macroautophagy (Korolchuk *et al,* 2011). These findings are consistent with previous work showing that non‐esterified fatty acids (NEFAs) disrupt lysosomal homeostasis and impair autophagic flux (Li *et al,* 2008; Las *et al,* 2011; Jaishy *et al*, 2015; Jaishy & Abel, 2016; Hung & Buhman, 2019), and that NEFAs induce cellular dysfunction in the absence of DGAT1 (Listenberger *et al,* 2003). Aside from lysosomal homeostasis, excess NEFAs also induce mitochondrial dysfunction, which might explain the increased NIX recruitment and mtROS levels observed upon DGAT inhibition (Penzo *et al,* 2002).

Our data suggest that DGAT1 inactivation compounds the metabolic effects of iron depletion, with reduced cell viability and impaired lysosomal homeostasis that restricts the efficiency of mitophagy. More work is needed to understand whether a particular subset of lysosomes exhibits selective vulnerability to lipid imbalance, which we predict could account for the modest yet consistent reduction in mitophagy. Lysosomes appeared to retain their overall acidity upon DGAT1/2 inhibition, but whether fusion dynamics, trafficking or autophagosome‐lysosome reformation might be affected is unclear. In agreement with our discovery that lipid homeostasis modifies mitophagy, a recent study reported a role for two lipid‐binding kinases (GAK and PRKCD) in Parkin‐independent mitophagy, and their inactivation induced endolysosomal dysfunction independently of effects on acidity (Munson *et al*, 2021). Of interest, the accumulation of mitochondrial DAG is hypothesised to play a role here, which is related to oxidative stress (Cowell *et al,* 2019).

Regardless, genetic inhibition of *Dgat1* (*mdy*) *in vivo* had profound effects on mitophagy, as revealed by *matrix*‐QC reporter flies. The additional locomotor phenotypes suggest this crosstalk has relevance for neural integrity. These striking findings clarify the physiological significance of our *in vitro* data, revealing an unexpected interplay with implications for our understanding of PINK1/Parkin‐independent mitophagy. It is noteworthy that previous phenotyping of mouse *Dgat1*/*Dgat2* double‐knockout white adipocytes demonstrated an accumulation of abnormal mitochondria and membranous whorls (Harris *et al*, 2011). Conversely, mitochondrial homeostasis was relatively unaffected in brown adipocytes from double *Dgat1/2* KO mice (Chitraju *et al*, 2020). Cardiomyocyte‐specific ablation of *Dgat1* also induced severe lipotoxic heart failure, mediated by DAG and ceramide (Liu *et al*, 2014). These observations emphasise the importance of cellular and metabolic context for these proteins. Our results further support the growing body of evidence highlighting the significance of LDs in safeguarding cellular integrity (Rambold *et al*, 2015; Chitraju *et al,* 2017; Nguyen *et al*, 2017; Benador *et al*, 2019). Given our evolving knowledge that LDs are more than mere storage organelles (Zechner *et al*, 2017; Xu *et al*, 2018; Olzmann & Carvalho, 2019; Salo & Ikonen, 2019; Henne *et al*, 2020), it will be exciting to explore whether pleiotropic LD subpopulations contribute to selective autophagy in different contexts. Considering the cell‐specific importance of lipid metabolism for neural integrity (Bailey *et al*, 2015; Shimabukuro *et al*, 2016; Marschallinger *et al*, 2020; Ralhan *et al*, 2021; Ramosaj *et al*, 2021), it will also be interesting to ascertain further how lipid signalling affects other mitophagy pathways in different physiological and pathological contexts. Consistent with this, the importance of lipid dysfunction in neurodegenerative disease and cancer continues to garner significant attention (Krahmer *et al*, 2013; Fanning *et al*, 2020; Snaebjornsson *et al*, 2020).

In summary, our results uncover metabolic crosstalk of fundamental importance to organelle quality control. Mitophagy is a compelling therapeutic target for age‐related pathology, particularly neurodegenerative disorders and cancer (Killackey *et al*, 2020). Yet even in the presence of defective mitophagy, additional mechanisms must operate to sustain cell and tissue integrity over decades. Therapeutic targeting of lipid metabolism may represent a lateral strategy to ensure efficient mitophagy. Indeed, clinically approved molecules targeting different aspects of lipid metabolism (*e.g*. statins) induce robust levels of autophagic turnover under certain conditions (Andres *et al*, 2014). It will be interesting to decipher how aberrant lipid homeostasis might exacerbate the effects of mitochondrial dysfunction, thereby compromising cellular integrity and promoting disease.

## Materials and Methods

### Mammalian cell culture

Human ARPE19 and U2‐OS cell lines were purchased from ATCC (Teddington, UK). The ARPE19 and SH‐SY5Y *mito*‐QC stable cell lines were generated by retroviral transduction of *mito*‐QC (Montava‐Garriga *et al*, 2020). Human fibroblasts were purchased from Coriell Institute for Medical Research (NJ, USA). All media and culture supplements were purchased from Gibco, Thermo Fisher Scientific, UK; foetal bovine serum (FBS, Origin: BR) was purchased from Life Science Production (LSP), UK. All cells were cultured in +37°C/5% CO_2_. ARPE19 and SH‐SY5Y cell lines were maintained in DMEM/F12 media with 10% inactivated FBS, 1% l‐Glutamine and penicillin‐streptomycin, whereas U2‐OS cells were maintained in DMEM media. Media were supplemented with 10% inactivated FBS, 1% l‐Glutamine and penicillin‐streptomycin. Human fibroblasts were maintained in Eagle's minimum essential media with Earle's salts with 15% FBS, 1% l‐Glutamine, 0.5% penicillin‐streptomycin and 1% non‐essential amino acids. For metabolomics and lipidomics studies, cells were used in experiments for up to 10 passages.

### Cell treatments

All compounds were purchased from Merck Sigma‐Aldrich unless otherwise stated. Deferiprone (DFP) treatments were performed for the length of time indicated in the figure legends using 1 mM DFP as previously described in (Allen *et al*, 2013; Zhao *et al,* 2020; Munson *et al*, 2021). Dimethyl sulfoxide (DMSO), DGAT1 and DGAT2 inhibitors (5 μM, PZ0207 and PZ0233, respectively) and SOAT1/2 inhibitor (2 μg/ml, S9318) were used to treat for the length of time indicated in figure legends. Unless otherwise indicated, cells were treated with DFP and 7 h later treated with lipid droplet inhibitors or DMSO. DFP and all inhibitors were freshly prepared prior to addition. 20 µM CCCP, 5 µM oligomycin, 10 µM antimycin A and 10 µM ivermectin (Zachari *et al,* 2019 *)* were used to treat cells for 24 h. Small interfering RNA (siRNA) oligonucleotides were purchased from Ambion (Thermo Fisher Scientific). Two siRNA oligonucleotides (#s16567 and #s16568 for DGAT1 and Negative Control 1 and 2) targeting the same transcript were pooled together, following determination of *DGAT1* silencing efficiency by RT‐qPCR. Oligofectamine (Life Technologies) was used to transfect cells for 72 h with Silencer Select siRNAs, following the manufacturer’s protocol.

### Stable cell line generation

To generate the retroviruses carrying the gene of interest, DNA constructs in pBABE vectors along with constructs for VSV‐G (vesicular stomatitis virus G protein) and Gag‐Pol (both from Takara Bio) were co‐transfected in HEK293‐FT cells. 6 μg of the pBABE plasmid, 3.8 μg of Gag‐Pol and 2.2 μg of VSV‐G were combined with 36 μl Lipofectamine 2000 in 600 μl Opti‐MEM (both from Thermo Fisher Scientific) for 20 min in RT. For the transfection, complexes were applied onto a 70% confluent dish of HEK293‐FT cells along with 5 ml of Opti‐MEM medium. Opti‐MEM medium was replaced with fresh DMEM culture medium after 5 h. The next morning, media were replaced with 10‐ml fresh culture medium. 24 h later, the medium containing formed viral particles was collected and filtered (0.45 μm pore size filter) and applied to cells along with 10 μg/ml polybrene to maximise infection efficiency. After 24 h, the medium was replaced with fresh medium and the next day cells were selected either with culture medium containing 2 μg/ml puromycin or with 100 μg/ml hygromycin.

### Antibodies and DNA constructs

For immunoblotting, the following primary antibodies were used: anti‐ULK1 [clone D8H5] (Cell Signaling Technology, Rabbit MAb, Cat# 8054S, 1:1,000, RRID:AB_11178668), anti‐ATG14 [clone D1A1N] (Cell Signaling Technology, Rabbit MAb, Cat# 96752. 1:1,000, RRID:AB_2737056), pS29 ATG14 [clone D4B8M] (Cell Signaling Technology, Cat# 92340S, 1:1,000, RRID:AB_2800182); anti‐Vinculin [clone EPR 8185], (Abcam, Rabbit MAb, Cat# ab129002; 1:1,000, RRID:AB_2800182. Secondary antibodies for immunoblotting were purchased from Thermo Fisher Scientific. For immunocytochemistry, the following primary antibodies were used: anti‐ATPB [clone 3D5] (Abcam, Cat# ab14730; Mouse mAb, 1:500; RRID:AB_301438); anti‐BNIP3L/Nix [clone D4R4B] (Cell Signaling Technology, Cat# 12396, Rabbit mAb, 1:100; RRID:AB_2688036); anti‐ULK1 [clone D8H5] (Cell Signaling Technology, Rabbit mAb, Cat# 8054S, 1:500, RRID:AB_11178668); anti‐WIPI2 (Bio‐Rad, Cat# MCA5780GA, Mouse mAb, 1:500; RRID:AB_11178668). Secondary antibodies for immunocytochemistry were purchased from Invitrogen (Life Technologies) and used at 1:500. The DNA constructs used (Flag‐ULK1 [DU45617], mCherry‐GFP‐FIS 101‐152 [DU55502]) were generated at the MRC PPU Reagents and Services and are available to purchase online https://mrcppureagents.dundee.ac.uk.

### Temporal metabolomics analysis by GC‐MS and LC‐MS

Culture dishes were placed on an ice‐cold surface, and cells were washed rapidly in ice‐cold PBS. 500 µl of 90% MeOH (HPLC‐grade Methanol, Fisher Scientific) including internal standards (13C3‐Caffeine, D‐sucrose‐13C12 both obtained from Merck Sigma‐Aldrich (St. Louis, MO, USA) were added to each well. Cells were rapidly harvested using a cell scraper, and 400 µl of extraction solution (including cells) from each well was transferred to pre‐chilled 1.5‐ml Sarstedt reaction tubes on dry ice. Frozen samples were stored at −80°C for further use. Metabolic profiling by GC‐MS and LC‐MS was performed at the Swedish Metabolomics Center in Umeå, Sweden. Prior to analysis, the metabolites were extracted as follows: To each sample, 100 µl of 90% MeOH including the following internal standards (13C9‐phenylalanine, D4‐cholic acid, salicylic acid‐D6, 13C9‐caffeic acid, succinic acid‐D4, l‐glutamic acid‐13C5,15N, putrescine‐D4, hexadecanoic acid‐13C4, d‐glucose‐13C6, obtained from Merck Sigma‐Aldrich (St. Louis, MO, USA), L‐proline‐13C5, alpha‐ketoglutarate‐13C4, myristic acid‐13C3, cholesterol‐D7 from Cil (Andover, MA, USA) and a tungsten bead was added. The samples were shaken at 30 Hz for 2 min in a mixer mill and centrifuged at +4°C, 14,000 rpm, for 10 min. 200 µl (LC‐MS) and 50 µl (GC‐MS) of the supernatant was transferred to micro‐vials, and solvents were evaporated to dryness. The dried vials were stored at −80°C until analysis. GC‐MS and LC‐MS analyses were performed as previously described (Diamanti *et al*, 2019). Briefly, the GC‐MS samples were derivatised in a total volume of 30 µl, and 0.5 µl of the derivatised sample was injected in splitless mode by an L‐PAL3 autosampler (CTC Analytics AG, Switzerland) into an Agilent 7890B gas chromatograph equipped with a 10 m × 0.18 mm fused silica capillary column with a chemically bonded 0.18 μm Rxi‐5 Sil MS stationary phase (Restek Corporation, U.S.) The injector temperature was 270°C, the purge flow rate was 20 ml/min, and the purge was turned on after 60 s. The gas flow rate through the column was 1 ml/min, the column temperature was held at 70°C for 2 min, then increased by 40°C/min to 320°C and held there for 2 min. The column effluent was introduced into the ion source of a Pegasus BT time‐of‐flight mass spectrometer, GC/TOFMS (Leco Corp., St Joseph, MI, USA).

Prior to LC‐MS analysis, the sample was re‐suspended in 10 + 10 µl methanol and Milli‐Q water. All samples were first analysed in positive mode. Thereafter, the instrument was switched to negative mode, and a second injection of each sample was performed. The chromatographic separation was performed on an Agilent 1290 Infinity UHPLC‐system (Agilent Technologies, Waldbronn, Germany). The compounds were detected with an Agilent 6550 Q‐TOF mass spectrometer equipped with a jet stream electrospray ion source operating in positive or negative ion mode. All parameters were described as previously (Diamanti *et al*, 2019). The samples were analysed in batches according to a randomised run order on both GC‐MS and LC‐MS. For the GC‐MS data, all non‐processed MS‐files from the metabolic analysis were exported from the ChromaTOF software in NetCDF format to MATLAB 2019b (Mathworks, Natick, MA, USA), where all data pre‐treatment procedures, such as base‐line correction, chromatogram alignment, data compression and Multivariate Curve Resolution were performed. Mass spectra and retention index comparison was performed using NIST MS 2.2 software. For the LC‐MS data, all data processing was performed using the Agilent Masshunter Profinder version B.10.00 (Agilent Technologies Inc., Santa Clara, CA, USA). The processing was performed in a targeted fashion. A pre‐defined list of metabolites (including amino acids, bile acids, acylcarnitines, fatty acids, lysophosphatidylcholines, nucleotides, short length peptides and steroids, among others) commonly found in human samples was searched for using the batch‐targeted feature extraction in Masshunter Profinder. The identification of the metabolites was based on MS, MSMS and retention time information and confirmed by authentic standards run on the same system with the same chromatographic and mass spectrometry settings.

### Temporal lipidomics analysis by LC/Q‐TOF‐MS

Culture dishes were placed on an ice‐cold surface, and cells were washed rapidly in ice‐cold PBS. 500 μl of 90% MeOH (HPLC‐grade Methanol, Fisher 526 Scientific) was added to each cell culture dish. Cells were rapidly harvested using a cell scraper, and 400 μl of extraction solution (including cells) from each well was transferred to pre‐chilled 1.5‐ml Sarstedt reaction tubes on dry ice. Frozen samples were stored at −80°C for further use. Lipidomic profiling by GC‐MS and LC‐MS was performed at the Swedish Metabolomics Center in Umeå, Sweden. Prior to analysis, the MeOH was evaporated under a steam of nitrogen and the lipid content was extracted following a modified folch protocol (Nygren *et al*, 2011; Orešič *et al*, 2012; Diab *et al*, 2019).

In detail, 50 µl of 0.15 M HCl and 250 µl extraction buffer (2:1 v/v chloroform:methanol) including internal standards (tripalmitin‐1,1,1‐13C3 and 16:0‐d31 ceramide) were added to the sample. The sample was shaken with two tungsten beads at 30 Hz for 3 min in a mixer mill, after which the beads were removed. The samples were let to stand at room temperature for 30 min. The sample was centrifuged at +4°C, 14,000 rpm, for 3 min. 200 µl of the lower phase were collected and divided into two different micro‐vials (60 + 120 µl) and stored at −80°C until analysis. The LC‐MS analysis of the lipid extracts was performed on an Agilent 1290 Infinity UHPLC‐system coupled to an Agilent 6540 Q‐TOF mass spectrometer (Agilent Technologies, Waldbronn, Germany) as described in Diab *et al,* 2019.

### RNA extraction, cDNA synthesis and RT‐qPCR

RNA was harvested from cells using the NucleoSpin RNA Plus kit (Macherey‐Nagel) following the manufacturer’s protocol. RNA concentration was measured via NanoDrop spectrophotometry and stored at −80°C until further use. Using a High‐Capacity cDNA Reverse Transcription kit (Applied Biosystems) and random hexamer primers, 600 ng of total RNA was transcribed into cDNA according to the manufacturer's protocol in a Bio‐Rad CFX96 PCR system. Real‐time quantitative PCR (RT‐qPCR) was carried out using 5x HOT FIREPol^®^ Probe Universal qPCR mix (Solis Biodyne) and 10 μM of pre‐designed primers and TaqMan probes (Merck Sigma‐Aldrich). Table 1 contains the complete list of primers and probes sequences used in this study. The delta‐delta CT method was used to analyse the RT‐qPCR results and determines fold changes in gene expression according to MIQE guidelines (Bustin *et al*, 2009). Three reference genes were used, namely *Actin*, *B2M* and *YWHAZ*.

**Table 1:** Taqman probes used in this study.

Taqman probe name	Taqman probe sequence
FH1_ACACA	AAAATCCACAATGCCAACCC
RH1_ACACA	TGTCAGCTGTTTCTCTAGCC
PH1_ACACA	[6FAM]GGCGCTGGTTTGTGGAAGTGGAAGG[OQA]
FH1_ACADL	TTGCATGGCGAAATATTGGG
RH1_ACADL	TAGATTGGCTGAACTCTGGC
PH1_ACADL	[6FAM]TGTGTACAGCTCCATGGAGGTTGGGGA[OQA]
FH1_ACADM	GTTCGGGGAGTATGTCAAGG
RH1_ACADM	AATACTTCTCAGGACCCTGC
PH1_ACADM	[6FAM]ACCCGTGTATTATTGTCCGAGTGGCCG[OQA]
FH1_ACADS	CAAGATAGCCATGCAAACCC
RH1_ACADS	TTCTCAGCGTAGTTCACAGC
PH1_ACADS	[6FAM]ATTGCCCAGACCGCCCTCGATTGT[OQA]
FH2_ACADVL	TCATGCCACTAATCGTACCC
RH2_ACADVL	TAGCACTCACCATGTAAGCC
PH2_ACADVL	[6FAM]TGGGCTGATCCAGGAGAAGCTGGCA[OQA]
FH1_ACAT1	GGTTCCATTGCAATTCAGGG
RH1_ACAT1	AAATAGGTAAGCCTGCACCC
PH1_ACAT1	[6FAM]GGACAAGCTCCTACAAGGCAGGCAGT[OQA]
FH1_ALDH9A1	AAGGAAGAGATCTTTGGGCC
RH1_ALDH9A1	CAGCTACCACTCTATGAGCC
PH1_ALDH9A1	[6FAM]GCAGCTGGCGTCTTTACCAGGGACA[OQA]
FH1_CPT1A	AAATCTCTACTACACGGCCG
RH1_CPT1A	ACATGACGTACTCCCAAAGG
PH1_CPT1A	[6FAM]ACGGGAAGATGGGCCTCAACGCTGA[OQA]
FH2_CPT1B	GGACATTCCAAAACAGTGCC
RH2_CPT1B	ACTGGAAGCAGTACAACTCC
PH2_CPT1B	[6FAM]AGTTCCTACCAGGTGGCCAAGGCGT[OQA]
FH1_CPT1C	GATCCCTGTTCAGCAAATGC
RH1_CPT1C	TAACCATGGTCATCAGCAGG
PH1_CPT1C	[6FAM]TGTTTCCTCAGGCGGTGGATTCGGG[OQA]
FH2_CPT2	AGATGATGGTTGAGTGCTCC
RH2_CPT2	AGGCAAGATGATCCCTTTGG
PH2_CPT2	AGCAATGGGCCAGGGCTTTGACCGA
FH1_CROT	CCCAAACCACATTGTAGTGC
RH1_CROT	TCCATCAGGTTCACTATGGC
PH1_CROT	[6FAM]TGTTTGGTCACCCCGCCAGAGCTT[OQA]
FH1_CYP51A1	GTTTTGGCTCAGTTGTTCCC
RH1_CYP51A1	GCAGAGAATTGCTTGAACCC
PH1_CYP51A1	[6FAM]TCTGTTGCCCAGGCTGGAGTGCAGT[OQA]
FH1_DHCR7	TGGAATCTTCTAAGGGCACG
RH1_DHCR7	ACAGATGAGGAAACTGAGGC
PH1_DHCR7	[6FAM]TGGGGCTGTCAAGAGCGTGTTCTGCCA[OQA]
FH1_EBP	AGTGTGTGGGTTCATTCACC
RH1_EBP	TCCCTTGGCATACTCTTTCC
PH1_EBP	[6FAM]TGGTGATCGAGGGCTGGTTCGTTCTC[OQA]
FH3_FDFT1	TCCTTTACCAACCAGACTGG
RH3_FDFT1	AACTCAAGGGAGATCGTTGG
PH3_FDFT1	[6FAM]GCAAGGAGAAGGATCGCCAGGTGCT[OQA]
FH1_FDPS	GGATGCTGATAGTCTCCAGC
RH1_FDPS	GATACCAGCAGATCTGTCCC
PH1_FDPS	[6FAM]TGGGCTGGTGTGTGGAACTGCTGCA[OQA]
FH1_HMGCR	GACCAACCTACTACCTCAGC
RH1_HMGCR	TAAGTGACAATTCCCCAGCC
PH1_HMGCR	[6FAM]TCCTGGGGAAAATGCCCGGCAGCTT[OQA]
FH3_LSS	GATCCATAACACATGCTGGG
RH3_LSS	CCCAGCAATGTTTTCCTGC
PH3_LSS	[6FAM]TTCGGCATCCTGACATCGAGGCCCA[OQA]
FH2_MVD	TGGAGACACGTTTCTGAAGG
RH2_MVD	GTCATCCAGGATTTGAGGCC
PH2_MVD	[6FAM]ACATCATTGTCACTCAGGTGGGGCCAG[OQA]
FH3_MVK	CAACATTGGTATCAAGCGGG
RH3_MVK	GTGTTGTGACATCACCTTGC
PH3_MVK	[6FAM]TGGGATGTGGCCAGGCTTCAGTCAC[OQA]
FH2_NR2F2	GGACCACATACGGATCTTCC
RH2_NR2F2	TGAGGTGAACAGGACTATGG
PH2_NR2F2	[6FAM]TGGAGAAGCTCAAGGCGCTGCACGT[OQA]
FH3_PMVK	CCACTCAAGGAACAGTATGC
RH3_PMVK	ATCTTCCTGCAAAAGAAGCC
PH3_PMVK	[6FAM]AGAGGAGAAACGCCAGGCTGACCCA[OQA]
FH2_PRDX4	GAGGAGTGCCACTTCTACG
RH2_PRDX4	GCTGTTCCTTCCCAGTAGG
PH2_PRDX4	[6FAM]TGGACAAGTGTACCCGGGAGAGGCA[OQA]
FH2_PRDX5	GACGTCTCAAGAGGTTCTCC
RH2_PRDX5	AGATGATATTGGGTGCCAGG
PH2_PRDX5	[6FAM]GGCCCTGAATGTGGAACCAGATGGCA[OQA]
FH3_SC5D	TAGCATACGGATCCGAGTCC
RH3_SC5D	CAACACGGAGTACAAGATCC
PH3_SC5D	AGGAGAGGCTGGCAGGGGCTAAGTGAT
FH1_SLC22A2	GATATCGGAGAACAGTGGGG
RH1_SLC22A2	GCAAGAAGAAGAAGTTGGGC
PH1_SLC22A2	[6FAM]TGCTAGCTGGGGTGGCTTACGCACT[OQA]
FH1_SLC22A4	TCAGGACTCGGAATATTGCC
RH1_SLC22A4	AGGAAACAGTTCAGGTAGGC
PH1_SLC22A4	[6FAM]GCTGCTATGGATGCTGACCTCAGTGGG[OQA]
FH2_SLC22A5	ATGGTCTACGTGTACACAGC
RH2_SLC22A5	GAAGTAGGGAGACAGGATGC
PH2_SLC22A5	[6FAM]AACATGGGTGTGGGAGTCAGCTCCACA[OQA]
FH1_SLC25A11	TTCTTCCTCTGCGGTAAAGG
RH1_SLC25A11	TCAGAAGTTTTCCAGCTCCC
PH1_SLC25A11	[6FAM]TCCAGCTTGCCCTGCTCGTCCTGAT[OQA]
FH3_SLC25A13	TCTACAAGTTGCAGAGTCGG
RH3_SLC25A13	CAGTTGATCGTTGGTTCTGC
PH3_SLC25A13	[6FAM]TGCTGGAGCTGTTGGAGCCACTGCT[OQA]
FH2_SOAT1	CGGAATATCAAACAGGAGCC
RH2_SOAT1	CGTAACATCTCAGCAAAGGC
PH2_SOAT1	[6FAM]CAGCGCTCGTGTTCTGGTCCTATGTGT[OQA]
FH1_SOAT2	CTGCTGCTCATCTTCTTTGC
RH1_SOAT2	CGGTAGAACATCCTGTCTCC
PH1_SOAT2	[6FAM]GCTCAACGCCTTTGCCGAGATGCT[OQA]
FH3_SQLE	GATATTCTCTCAGGCCTGCC
RH3_SQLE	TATTGGTTCCTTTTCTGCGC
PH3_SQLE	[6FAM]AAGGAGCAGCTCGAGGCCAGGAG[OQA]
FH2_SREBF1	CCTGACCATCTGTGAGAAGG
RH2_SREBF1	AAGAAGCAGGTCACACAGG
PH2_SREBF1	[6FAM]GCAGCTCCATTGACAAGGCCGTGCA[OQA]
FH3_SREBF2	TGCCCTTCAAGTACCAACC
RH3_SREBF2	TCTTGCCCCATCATTACAGG
PH3_SREBF2	[6FAM]TGGTGGGCAGCAGTGGGACCATTCT[OQA]
FH1_TMLHE	CAGTTTTGGGGGTGAAAAGG
RH1_TMLHE	GTAGGTGGGACAATCTGTGG
PH1_TMLHE	[6FAM]AAAAGGCGGGTGAAAGGCTGCCTCC[OQA]

### Cell dyes

BODIPY™ 493/503, LipidTOX™, and MitoTracker Red CMX ROS, MitoSOX™ Red Mitochondrial Superoxide Indicator (Thermo Fisher Scientific) (Thermo Fisher Scientific) were used according to the manufacturer’s guidelines. Cathepsin‐reactive lysosomes were visualised using Magic Red (Bio‐Rad, ICT937) according to the manufacturer’s guidelines and as described (Bright *et al,* 2016) with minor modifications. Briefly, cells were seeded 48 h before the measurement and treated with DFP ± DGAT1/2 inhibitors as previously outlined above before co‐incubation with Magic Red™ (1:26 dilution of stock solution as per manufacturer's protocol) for 30 and 5 min with Hoescht 33342 (1 µg/ml) followed by live‐cell imaging as described below.

### Immunocytochemistry

Immunocytochemistry was performed as previously described (Allen *et al,* 2013; McWilliams *et al,* 2016). Briefly, cells cultured on coverslips were washed briefly in temperature‐equilibrated PBS followed by fixation using 3.7% formaldehyde in 0.2 M HEPES (as above) for 20 min, washed twice with quench solution (DMEM/HEPES) and incubated for 10 min in DMEM/HEPES at room temperature. Following a single wash in PBS, a 5‐min permeabilization step was performed using 0.2% Triton X100, after which samples were washed 2× and blocked in 1% BSA/PBS and incubated for 15 min with gentle agitation. Primary antibodies were diluted in 1% BSA/PBS and applied to samples in humidified chambers and incubated for 1 h at +37°C. Following three 10 min washes in PBS/BSA with gentle agitation, secondary antibody was applied for 30 min at room temperature, protected from light. Samples were counterstained with Hoescht 33342 and washed three times for 10 min each in PBS/BSA, prior to mounting in Vectashield H‐1000.

### Image acquisition and microscopy

Cells were grown on glass coverslips and cultured/treated as described above. ARPE19 *mito*‐QC cells were fixed in 3.7% PFA at pH 7.0 in 0.2 M HEPES, counterstained with Hoechst 33342 and VECTASHIELD Antifade Mounting Medium H‐1000 was used to mount coverslips on slides. Images were acquired using an ANDOR Spinning Disc Microscope equipped with a Zyla camera (Plan Apochromat ×40 objective, NA 1.15). For high‐content temporal analysis of LD biogenesis, ARPE19 cells were seeded on Ibidi black wall *m* plates (24 well format) with culture and treatment conditions as described above. Samples were labelled with BODIPY and MitoTracker and fixed as described above, followed by imaging with a PerkinElmer Opera Phenix Platform. For analysis of LDs in *ULK1* KO cells, images were acquired using a wide‐field Nikon Eclipse Ti wide‐field microscope using a Nikon Plan Apo ×60 oil immersion objective.

### Live‐cell imaging

Cells were grown in 35‐mm glass bottom dishes (Ibidi) and cultured/treated as described above. Prior to imaging, samples were incubated with 2 μM of BODIPY™ 493/503 (Thermo Fisher Scientific) and 5 nM MitoTracker Red CMX ROS (Thermo Fisher Scientific) in cell culture media for 30 min at +37°C/5% CO_2_. Cells were washed with warm media and incubated in media at +37°C/5% CO_2_ before proceeding with live‐cell imaging using an ANDOR Spinning Disc Microscope equipped with a Zyla camera (Plan Apochromat ×40 objective, NA 1.15) and an optical zoom of 1.5. Images were acquired every 10 ms for a total of 30 s. Live cell videos were processed with Imaris software (Bitplane, Zürich, Switzerland). For mitochondrial ROS imaging, cells were incubated with 2 μM of MitoSOX (Thermo Fisher Scientific) for 30 min at +37°C/5% CO_2_. Cells were washed with warm media and incubated in media at +37°C/5% CO_2_ before proceeding with live‐cell microscopy using an ANDOR Spinning Disc Microscope equipped with a Zyla camera (Plan Apochromat ×40 objective, NA 1.15). For imaging cathepsin‐reactive endolysosomes, a × 100 SR Apo TIRF Objective, NA 1.49 with Andor Zyla 4.2 sCMOS camera was used.

### Image analysis and quantitation

Images were analysed with CellProfiler (Carpenter *et al*, 2006), or Fiji (ImageJ; NIH) (Schindelin *et al*, 2012). BODIPY™ 493/503 or LipidTOX™ stained cells were processed using a CellProfiler pipeline developed to segment and count individual objects and measure area occupied by the object. Mitochondria‐lipid droplet proximity was analysed manually by counting the total number of lipid droplets in a cell and the proportion of these adjacent to mitochondria. For all analyses, images were obtained using uniform random sampling. All images in each experimental group were processed as a batch using identical protocols. Mitophagy levels were analysed using Fiji *mito*‐QC counter macro as described in (Montava‐Garriga *et al*, 2020). Briefly, individual cells were segmented manually in Fiji and saved as regions of interest for each image. The mCherry and GFP signals were thresholded using Fiji mito‐QC counter macro with the following parameters: radius for smoothing images = 1, ratio threshold = 1 and red channel threshold = mean + 1 SD. The resulting parameters (e.g. number of mitolysosomes, mitolysosomes size and mitochondrial content) were calculated for each cell (Montava‐Garriga *et al*, 2020). Images acquired using high‐content platform were processed and analysed with Harmony software (PerkinElmer). Data analysis for lysosome positioning was performed using Image J/Fiji (NIH) as previously described (do Couto *et al*, 2020). Briefly, perinuclear regions were defined according to Hoescht labelling and concentric zones were applied to determine the cell peripheral region. After thresholding, lysosomal numbers were counted for each perinuclear and cell peripheral region.

### Western blotting

For Western blotting, cells were washed twice in ice‐cold PBS and were subsequently scraped in lysis buffer (50 mM Hepes, pH 7.4, 150 mM NaCl, 1 mM EDTA, 10% glycerol, 0.5% NP‐40 and protease/phosphatase inhibitor cocktails. Lysates were incubated on ice for 20 min, following centrifugation at 20,000 *g* at 4°C. The supernatant was then transferred to a new tube, and lysates were subjected to protein content estimation using Bio‐Rad Protein Assay Dye Reagent Concentrate (Bio‐Rad), according to the manufacturer’s instructions. Samples were prepared in 1× (LDS) lithium dodecyl sulphate buffer (Thermo Fisher Scientific) before loading on homemade 10% Bis‐Tris for electrophoresis following standard protocols. Gels were next subjected to wet transfer onto PVDF membranes. Membranes were blocked in 5% milk in TBS‐Tween for 30 min in RT, following washes in TBS‐Tween and primary antibody incubation O/N at 4°C. Antibody dilutions were as follows: for ULK1, ATG14, pS29 ATG14 1:1,000 in 5% BSA/TBS‐Tween, for Vinculin 1:10,000 in 5% BSA/TBS‐Tween. Upon primary antibody incubation, membranes were washed three times in TBS‐Tween, followed by secondary antibody (Thermo Fisher Scientific) incubation for 60 min in RT. Membranes were then washed three times in TBS‐Tween, followed by development with a Chemidoc imaging system (Bio‐Rad). For treatments, cells were at ~90% confluency for Western blotting.

### Flow cytometry

SH‐SY5Y and ARPE19 cells, stably expressing mcherry‐GFP‐FIS1, were grown on 6‐cm dishes until reaching 70% confluency and treated with either DMSO or PF‐04620110 (5 μM) and PF‐06424439 (5 μM), as indicated, for 24 h in the presence or absence of DFP. Cells were harvested for analysis by washing once with PBS, followed by trypsinisation with trypsin–EDTA (0.25%) (Thermo Fisher Scientific). The cells were then fixed in 3.7% PFA/10 mM Hepes, pH 7.0, for 15 min and finally re‐suspended in 0.4 ml of Dulbecco’s PBS containing 1% FBS into 5‐ml Falcon round‐bottom polystyrene test tubes 12 × 75 mm (Thermo Fisher Scientific).

Flow cytometry data were acquired on an LSR Fortessa II with DIVA software (BD Biosciences). Cells were gated according to their forward‐ and side‐scatter profiles. 488‐nm laser was used to detect GFP in emission filter 530/30 and 561‐nm laser to detect mCherry in emission filter 610/20. Data were analysed using FlowJo software v10.7.1 (BD Biosciences). 20–50,000 cells were analysed per condition, with fluorescent detection in green and red channels. Increased mitophagy was determined for individual cells by detecting decreased green versus red fluorescence, based on gating determined by the green and red fluorescence of vehicle (DMSO)–treated control cells. For treatments, cells were at 60–70% confluency for FACS on the day of the experiment.

### Cell proliferation assay

24 h prior to the experiment, 30,000 ARPE19 cells were seeded per well of a 24‐well plate (Corning^®^). The next day, cells were treated in duplicate with 1 mM DFP; 7 h later, cells were treated with lipid droplet inhibitors or DMSO. 7 μM puromycin (Merck Sigma‐Aldrich) was used as a positive control. Phase contrast images were acquired using the IncuCyte ZOOM live‐cell analysis system equipped with a 10× air objective for a total of 35 h. Images were acquired every 2 h, and 9 fields of view were imaged per well. Cell proliferation and confluency were analysed with a pipeline designed with the in‐built ZOOM analysis system. Briefly, cellular debris was removed by limiting the size of the detected object, and rolling ball algorithm removed the background uniformly. The initial numbers of cells in the wells were used for cell number normalisation. Proliferation of cells is depicted as a log2 fold change.

### *Drosophila* experiments

Flies were raised under standard conditions in a temperature‐controlled incubator with a 12 h:12 h light:dark cycle at 25°C and 65% relative humidity, on food consisting of agar, cornmeal, molasses, propionic acid and yeast. The following strains were obtained from the Bloomington *Drosophila* Stock Centre (RRID:SCR_006457): ubiquitous *da*‐*GAL4* driver (RRID: BDSC_55850), muscle‐specific *Mef2*‐*GAL4* driver (RRID: BDSC_27390), pan‐neuronal‐specific *nSyb*‐*GAL4* driver (RRID: BDSC_68222) and *UAS‐mdy RNAi* (RRID: BDSC_65963). Additional RNAi lines were obtained from the Vienna *Drosophila* Resource Centre for *mdy*—v6367 (GD) and v100003 (KK)—and *lacZ* control, v51446 (Dietzl *et al*, 2007). The *matrix‐*QC reporter line was generated by fusing the mCherry‐GFP coding sequences from *mito‐*QC (Lee *et al,* 2018) to the mitochondrial targeting sequence of COXVIII from *UAS‐mito‐HA‐GFP* (RRID: BDSC_8842). This was cloned into pUAST.attB transgenesis vector and inserted into the attP16 genomic landing site via phiC31‐mediated integration.

#### Drosophila behaviour

For locomotor assays, climbing (negative geotaxis assay) was assessed as previously described using the pan‐neuronal‐specific driver nSyb‐GAL4, with minor modifications (Greene *et al,* 2003). Viability assays were performed by following the developmental stages from embryo to fully mature adults.

#### Drosophila mitophagy reporter animals

Analysis of mitolysosomes was done as previously described (Lee *et al,* 2018). Briefly, spinning disc microscopy–generated images from dissected larval brains were processed using IMARIS (version 9.0.2) analysis software (BitPlane; RRID:SCR_007370) to identify and count individual red‐only puncta. The GFP and mCherry signals were adjusted to reduce background noise and retain only the distinct mitochondrial network and red‐only puncta, respectively. A surface‐rendered 3D structure corresponding to the mitochondria network was generated using the GFP signal. This volume was subtracted from the red channel to retain the mCherry signal that did not colocalise with the GFP‐labelled mitochondria network. The mitolysosome puncta were selected according to their intensity and an estimated size of 0.5 µm diameter, previously measured with IMARIS. Additionally, the puncta were filtered with a minimum size cut‐off of 0.2 µm diameter. The remaining puncta were counted as the number of mitolysosomes. Larval CNS soma was analysed individually where discrete cells could be distinguished.

### Statistical analysis and figures

**Figure 8:**
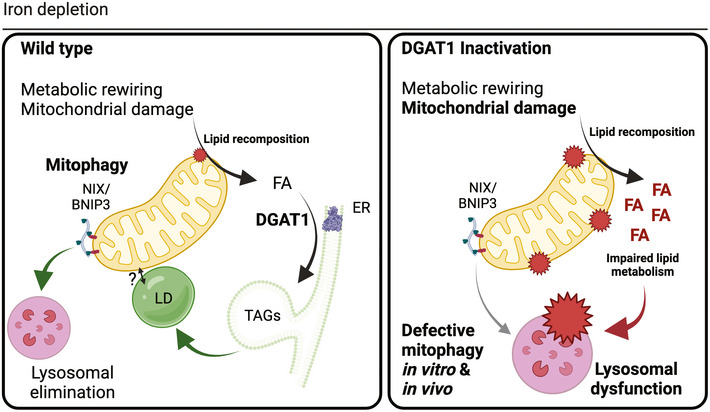
DGAT1 and mitophagy synergise to safeguard cell and tissue integrity Iron depletion rapidly reshapes the cellular metabolome. DFP treatment alters glucose utilisation to promote lipid biosynthesis and TAG storage in lipid droplets via DGAT1 activity, upstream of NIX‐dependent mitochondrial clearance. Without DGAT1, fatty acids cannot be esterified into TAG, compounding lipid dysfunction that impairs lysosomal homeostasis, leading to inefficient mitophagy and promoting cell death. Strikingly, genetic depletion of *DGAT1 in vivo* also impairs basal mitophagy, demonstrating the physiological relevance of our *in vitro* findings.

Statistical analyses were performed in GraphPad Prism v 8.0. Student’s *t*‐test was used for pairwise comparisons, whereas multiple comparisons were analysed with one‐way analysis of variance (ANOVA) and Bonferroni’s *post‐hoc* test where indicated in the figure legends. Multivariate modelling for metabolomics was conducted using SIMCA software v.16 (Umetrics, Sartorius Stedim Data Analytics, Umeå, SE), and MetaboAnalyst, version 4.0 and 5.0 (https://www.metaboanalyst.ca/; Xia *et al,* 2009; Xia & Wishart, 2011). Heatmaps were generated using Morpheus (https://software.broadinstitute.org/morpheus/). For *Drosophilia* motor function assays, groups of flies used were blinded by a different investigator than the one performing the assay, and a Kruskal–Wallis nonparametric test with Dunn’s *post‐hoc* correction for multiple comparisons was used. For analysis of tissue mitolysosomes, images were blinded prior to analysis and statistical significance was calculated by one‐way ANOVA with Šidák’s post‐test correction for multiple samples. For all the analyses, samples were collected and processed simultaneously, and therefore, no randomisation was appropriate. Figures were assembled in Adobe Illustrator, and graphical illustrations or elements in Figs 1, 6, 7 and 8 were created with BioRender.com (https://biorender.com/).

## Author contributions

**Maeve Long:** Conceptualization; Data curation; Formal analysis; Investigation; Visualization; Methodology; Writing—original draft; Writing—review & editing. **Alvaro Sanchez‐Martinez:** Data curation; Formal analysis; Validation; Investigation; Visualization; Methodology; Writing—review & editing. **Marianna Longo:** Data curation; Formal analysis; Validation; Investigation; Visualization; Methodology; Writing—review & editing. **Fumi Suomi:** Data curation; Formal analysis; Validation; Investigation; Visualization; Methodology; Writing—review & editing. **Hans Stenlund:** Resources; Data curation; Software; Formal analysis; Investigation; Visualization; Methodology; Writing—original draft; Writing—review & editing. **Annika Johansson:** Resources; Data curation; Software; Supervision; Validation; Investigation; Methodology; Writing—original draft; Writing—review & editing; Project administration. **Homa Ehsan:** Data curation; Validation; Investigation; Writing—review & editing. **Veijo Tuomas Verneri Salo:** Writing—review & editing. **Lambert Montava‐Garriga:** Software; Methodology; Writing—review & editing. **Seyedehshima Naddafi:** Formal analysis; Validation; Methodology; Writing—review & editing. **Elina Ikonen:** Resources; Supervision; Funding acquisition; Methodology; Writing—review & editing. **Ian G Ganley:** Resources; Supervision; Funding acquisition; Validation; Visualization; Methodology; Project administration; Writing—review & editing. **Alexander J Whitworth:** Resources; Supervision; Funding acquisition; Validation; Visualization; Methodology; Project administration; Writing—review & editing. **Thomas McWilliams:** Conceptualization; Resources; Supervision; Funding acquisition; Visualization; Methodology; Writing—original draft; Project administration; Writing—review & editing.

In addition to the CRediT author contributions listed above, the contributions in detail are:

The authors AS‐M, MLongo, FS, and HS equally contributed to this work.

## Disclosure and competing interests statement

L.M.G. is now an employee of AstraZeneca plc. E.I. is an EMBO member; this has no bearing on the editorial consideration of this article for publication. The other co‐authors declare no competing interests or disclosures.

## Supporting information



Appendix

Expanded View Figures PDF

Movie EV1

Movie EV2

Movie EV3

Movie EV4

## Data Availability

This study includes no data deposited in external repositories.
