# Supplementary material for: DGAT1 activity synchronises with mitophagy to protect cells from metabolic rewiring by iron depletion
Source: EMBO J. 2022 Apr 12;41(10):e109390. doi: 10.15252/embj.2021109390 (PMC9108618; doi:10.15252/embj.2021109390)

APPENDIX PDF

EMBOJ-2021-109390R

DGAT1 activity synchronises with mitophagy to protect cells from metabolic rewiring by iron depletion

Maeve Long[1,7], Alvaro Sanchez-Martinez[6‡], Marianna Longo[5‡], Fumi Suomi[1‡], Hans Stenlund[4‡], Annika I. Johansson[4], Homa Ehsan[1], Veijo T. Salo[1,2,3,9], Lambert Montava-Garriga[5,8], Seyedehshima Naddafi[1,2], Elina Ikonen[1,2,3], Ian G. Ganley[5], Alexander J. Whitworth[6], Thomas G. McWilliams[1,2]*

*****_Correspondence_: thomas.mcwilliams@helsinki.fi

Table of contents

Page 2 – Supplementary Figure Legends
Page 3 – Appendix Figure S1
Page 4 – Appendix Figure S2

Supplementary Figure Legends

Appendix Figure S1:
Analysis of LD biogenesis in response to different mitophagy stimuli.

S1a. Representative photomicrographs of ARPE19 cells treated for 24 h with the compound indicated on the image in the following amount; DFP (1mM), CCCP (20 µM), Oligomycin (5 μM), Antimycin A (10 μM), Ivermectin (10 μM). Cells were stained with BODIPY™ and fixed. Nuclei are counterstained with Hoescht33342. Scale bar = 5 μm.
S1b. Associated quantitation for S4a.

Appendix Figure S2:
Aberrant lipid metabolism upon loss of LD biogenesis.

S2a. Comparative modelling of the lipidome between DFP vs. DFP+DGAT1i/2i at 24 h. OPLS-DA OPLS-DA was performed using MetaboAnalyst from LC-MS datasets described above.
S2b. Heatmap showing enrichment and loss of lipid species upon loss of LD biogenesis. Each coloured cell on the map corresponds to a z-score value. Whilst DFP-induced TAGs are depleted upon DGAT1i/2i inhibition, a broad enrichment signature of several other lipid species is evident, including cytotoxic ceramides and glycerophospholipids. All samples from *n*=3 experiments are shown.
S2c. Average values of S7b shown for simplicity.

Appendix Figure S1

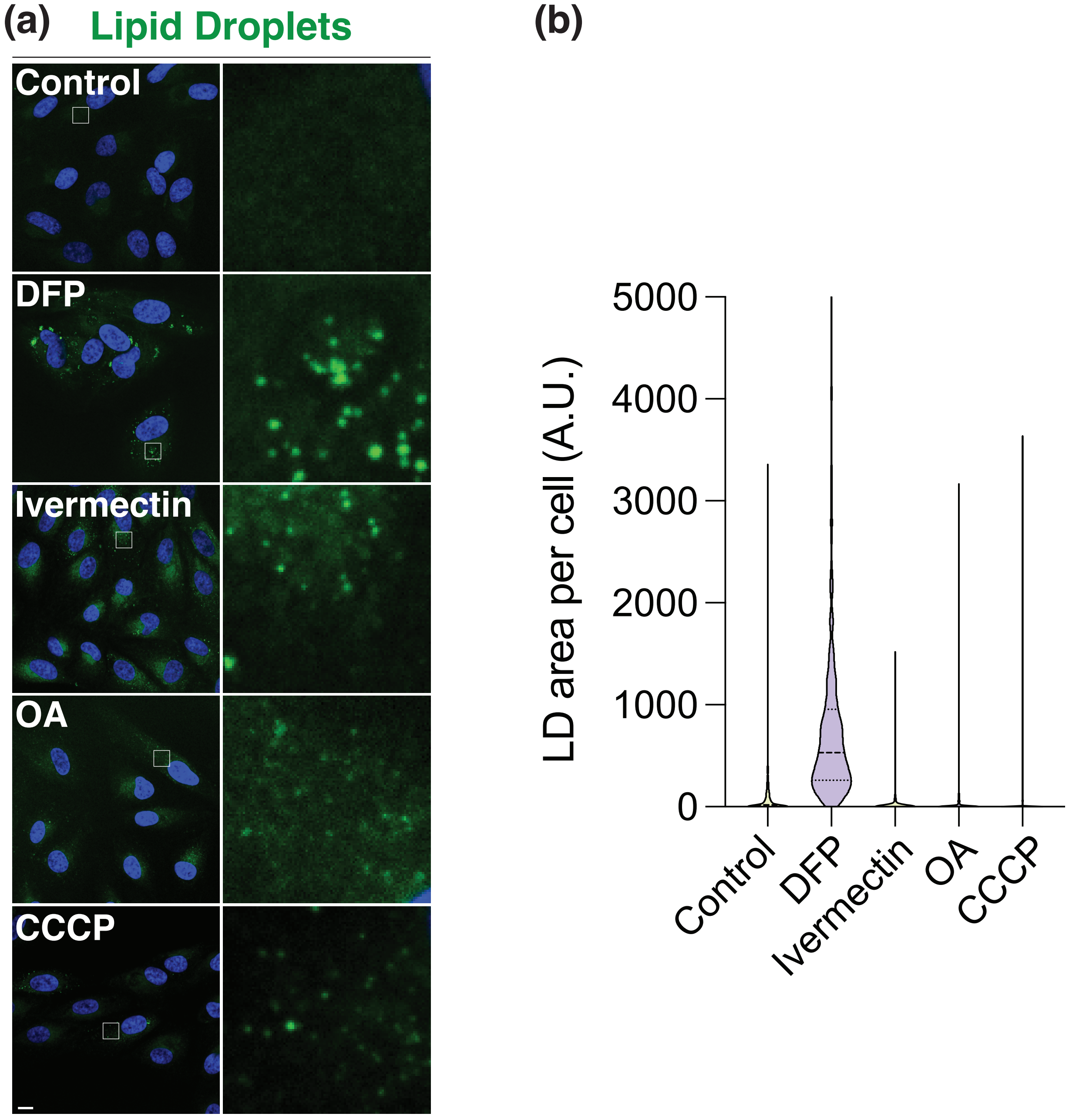

Appendix Figure S2

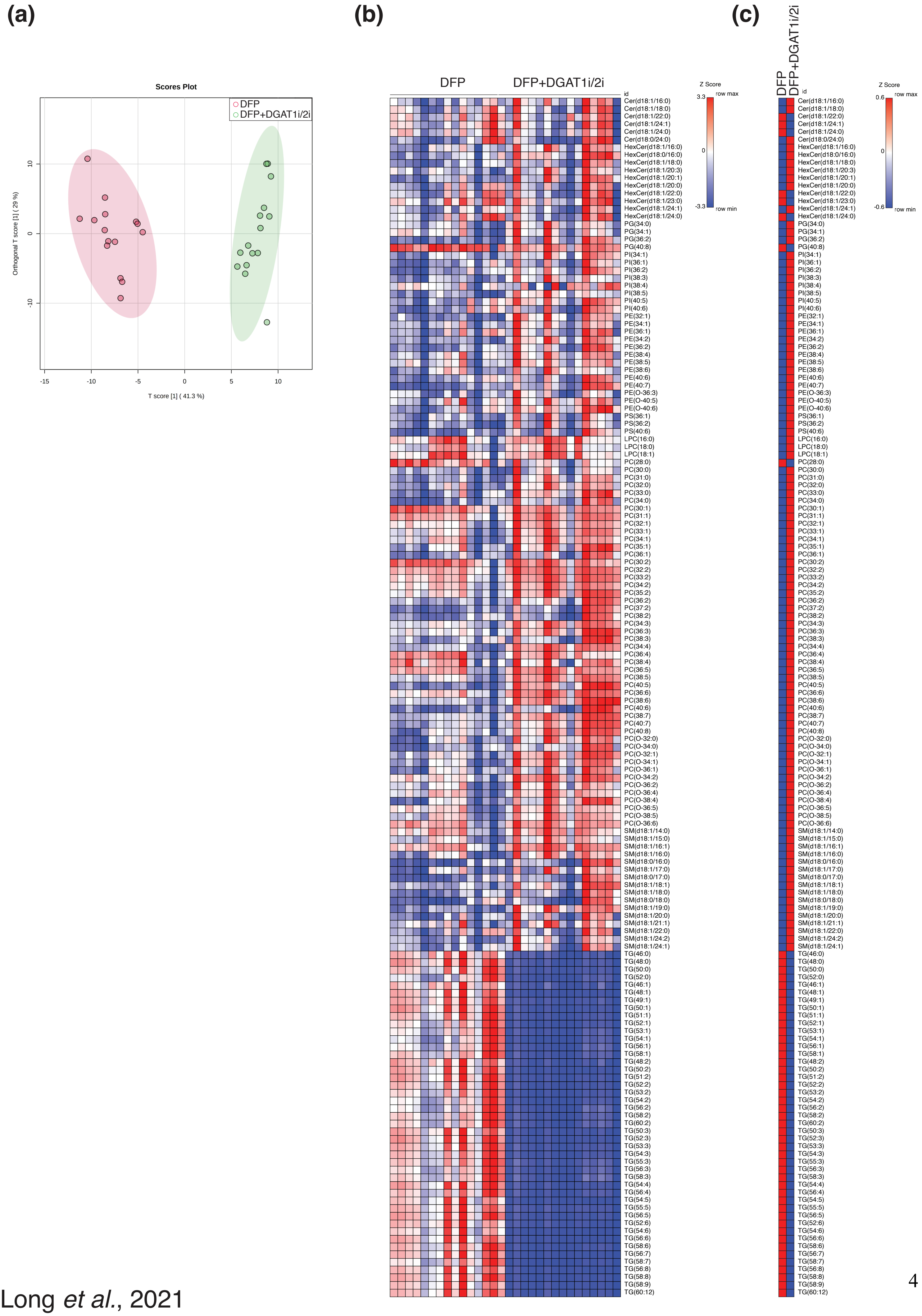

Supplement: Supplementary file 1 — Appendix [file EMBJ-41-e109390-s006.pdf]
