# Supplementary material for: DGAT1 activity synchronises with mitophagy to protect cells from metabolic rewiring by iron depletion
Source: EMBO J. 2022 Apr 12;41(10):e109390. doi: 10.15252/embj.2021109390 (PMC9108618; doi:10.15252/embj.2021109390)
Supplement: Supplementary file 2 — Expanded View Figures PDF [file EMBJ-41-e109390-s004.pdf]

Expanded View Figures

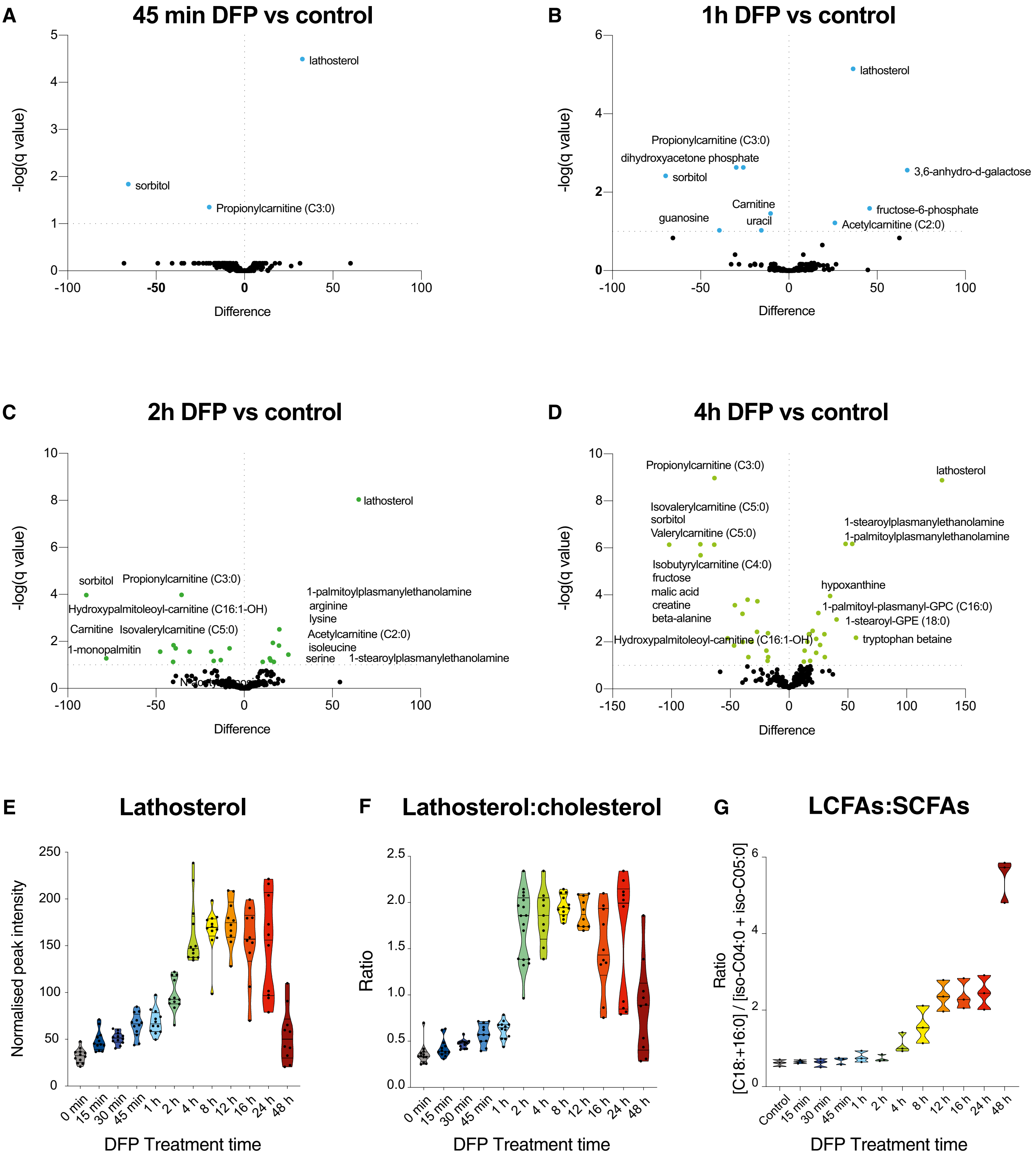

Figure EV1.

© 2022 The Authors

© 2022 The Authors

Figure EV1. Additional volcano plots and ratios of affected metabolites upon loss of cellular iron.

A–D Volcano plots representing the difference in the mean of metabolites from DFP-treated cells compared with control cells at acute (45 min) and at 1, 2 and 4 h timepoints, generated by unpaired T test with an FDR of 0.01. Coloured points are significantly altered.

E Graph of lathosterol peak intensities over time (n = 3 biological experiments with 4 technical replicates per timepoint in each experiment).

F Ratio of peak intensities of lathosterol over cholesterol upon DFP treatment over time. All data are derived from (n = 3 biological experiments with 4 technical replicates per timepoint in each experiment).

G Ratio of peak intensities of long-chain fatty acids (LCFA) over short-chain fatty acids (SCFA) upon DFP treatment over time (n = 3 biological experiments with 4 technical replicates per timepoint in each experiment).

Figure EV2. Iron depletion drives selective acylcarnitine dysregulation.

A OPLS-DA (5 + 1) model fitted for control and DFP samples for acylcarnitines (N = 136, K = 30). Hierarchical cluster analysis (HCA) for the p1 and p2-loadings, calculated with Ward. Arbitrary threshold of 0.03 revealed seven sub-groups for acylcarnitines.

B Graphs represent peak intensities of acylcarnitine metabolites over time revealing selective time-dependent alterations upon DFP treatment. All graphs are represented as mean ± SEM, n = 3 with 4 technical replicates per timepoint.

© 2022 The Authors

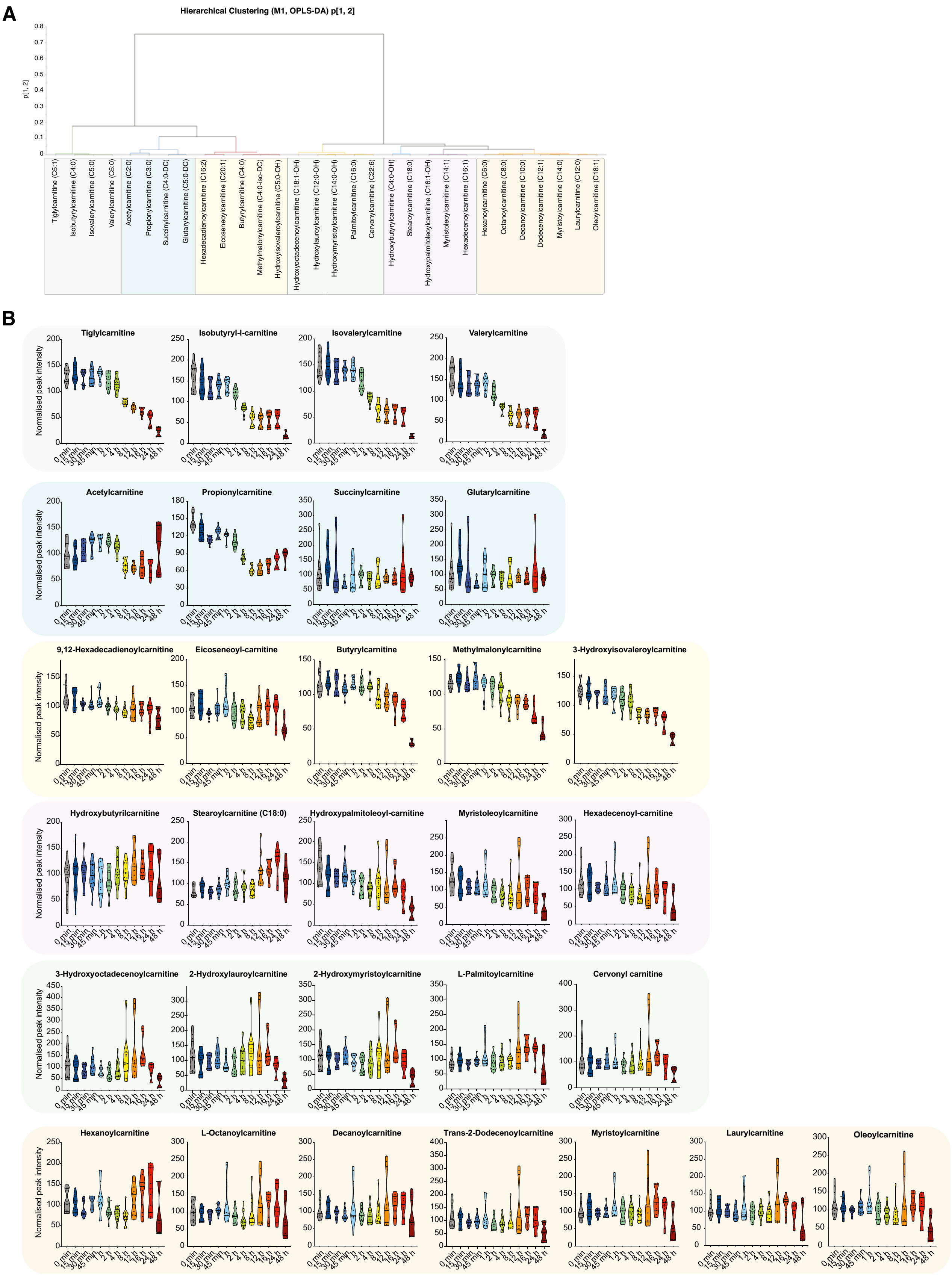

Figure EV2.

© 2022 The Authors

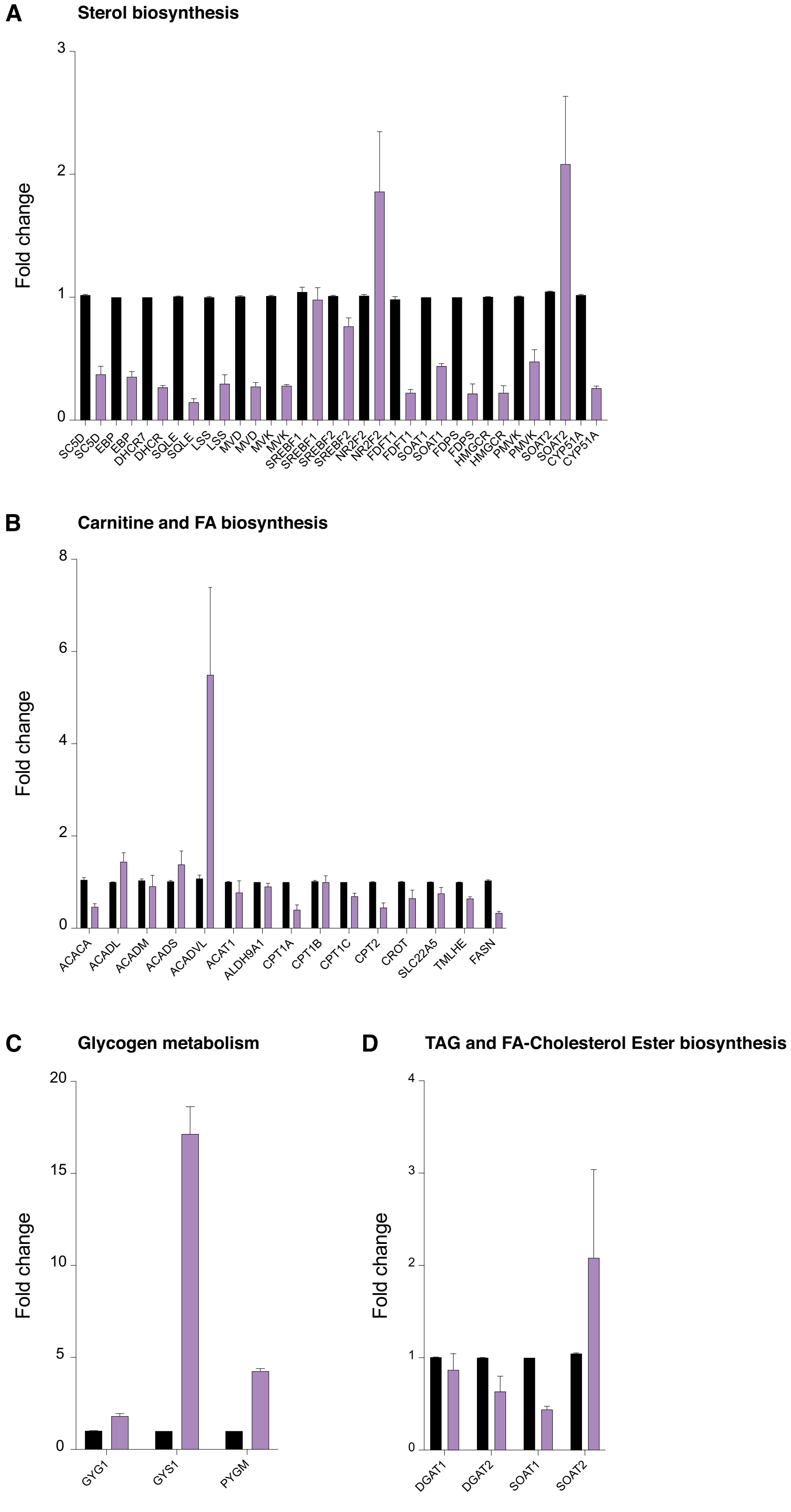

Figure EV3. Gene expression analysis by RT-qPCR upon iron depletion.

A Gene expression analysis by TaqMan RT-qPCR of mRNA transcripts encoding regulatory enzymes in carnitine and fatty acid biosynthesis in control and 24-h DFP (1 mM)-treated cells.

B Gene expression analysis by TaqMan RT-qPCR of mRNA transcripts encoding regulatory enzymes in sterol biosynthesis in control and 24-h DFP (1 mM)-treated cells.

C Gene expression analysis by TaqMan RT-qPCR of mRNA transcripts encoding regulatory enzymes in glycogen biosynthesis in control and 24-h DFP (1 mM)-treated cells.

D Gene expression analysis by TaqMan RT-qPCR of mRNA transcripts encoding regulatory enzymes in triglyceride and fatty acid-cholesterol ester biosynthesis in control and 24-h DFP (1 mM)-treated cells.

© 2022 The Authors

Figure EV4. Iron depletion-induced LD biogenesis occurs via DGAT1 in numerous cell subtypes.

A Representative photomicrographs of human dermal fibroblasts treated for 24 h with DFP in the presence or absence of inhibitors to DGAT1 (DGAT1i), DGAT2 (DGAT2i) or DGAT1 and DGAT2 (DGAT1i/2i).

B Representative photomicrographs of human U2-OS osteosarcoma cells, treated as in S5a.

C Representative photomicrographs demonstrating the effects of RNAi-mediated DGAT1 depletion upon in human ARPE19 cells, upon 24 h DFP treatment (si = small interfering; siControl refers to scrambled or non-targeting siRNA).

Data information: In all experiments A–C, cells were stained with BODIPY to visualise lipid droplets and fixed. Nuclei were counterstained with Hoescht 33342. Scale bars = 5 µm. Quantitation accompanies each respective panel.

 © 2022 The Authors

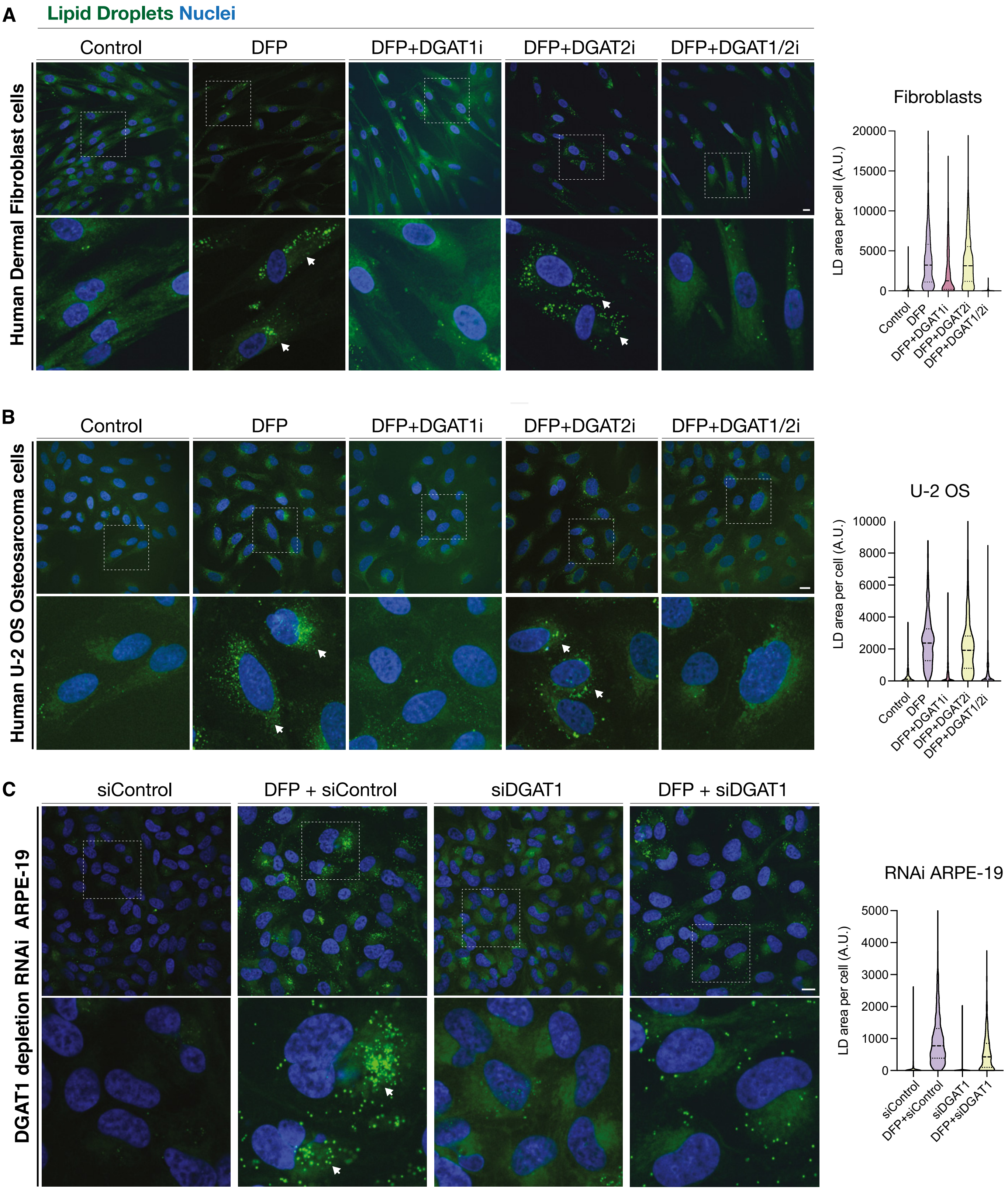
A
Lipid Droplets Nuclei
Control
DFP
DFP+DGAT1i
DFP+DGAT2i
DFP+DGAT1/2i
Human Dermal Fibroblast cells
Fibroblasts
LD area per cell (A.U.)
20000
15000
10000
5000
0
B
Human U-2 OS Osteosarcoma cells
U-2 OS
10000
8000
6000
4000
2000
C
siControl
DFP + siControl
siDGAT1
DFP + siDGAT1
DGAT1 depletion RNAi ARPE-19
RNAi ARPE-19
5000
4000
3000
2000
1000
DFP+siControl
DFP+siDGAT1

Figure EV4.

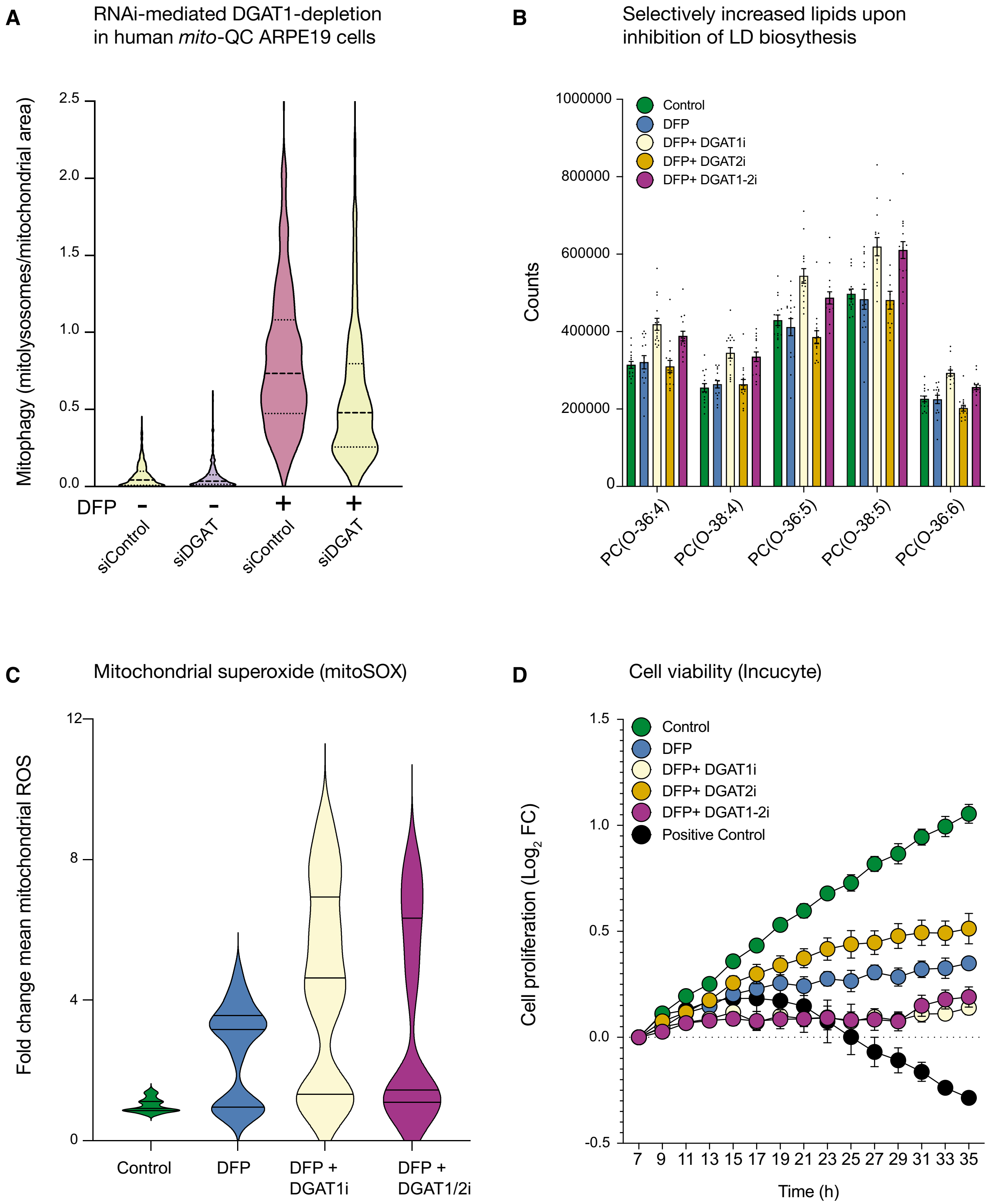

Figure EV5.

 © 2022 The Authors

Figure EV5. Impaired LD biogenesis promotes lipid dysfunction, oxidative stress and impaired viability upon iron depletion.

A DFP-induced mitophagy is significantly reduced upon RNAi-mediated depletion of *DGAT1* in human ARPE19 *mito*-QC cells (n = 3 experimental replicates).

B DGAT1 inhibition specifically increases ether-linked phosphatidylcholines during iron chelation. Graph of the amount of specific lipid species measure in conditions indicated on figure (i denotes the presence of inhibitor, for example, DGAT1i = DGAT1 inhibition) and displayed as mean ± SEM (n = 3, with 4 technical replicates per biological replicate).

C Inhibition of DGAT1-dependent LD biogenesis drives mtROS production. Cells treated with DFP (1 mM) for 24 h and LD inhibitors (5 μM) for 17 h were incubated with MitoSOX and imaged by spinning disc confocal imaging (i denotes the presence of inhibitor, for example, DGAT1i = DGAT1 inhibition). Graph of the mean fold change in fluorescence intensity ± SEM (n = 3 with at least 70 cells quantified per biological replicate for each condition).

D DGAT1 inhibition worsens the effect of iron chelation on cell proliferation. ARPE19 cells treated with DFP (1 mM) in combination with DGAT1 inhibitors (5 μM) ceased to proliferate in comparison with cells treated with DFP only. Positive control cells are treated with puromycin (7 μM). Results are presented as mean ± SEM of Log_2 fold change, n = 3 experimental replicates; each experimental replicate is the average of technical duplicates.

© 2022 The Authors
