# Supplementary material for: DGAT1 activity synchronises with mitophagy to protect cells from metabolic rewiring by iron depletion
Source: EMBO J. 2022 Apr 12;41(10):e109390. doi: 10.15252/embj.2021109390 (PMC9108618; doi:10.15252/embj.2021109390)
Supplement: Supplementary file 7 [file EMBJ-41-e109390-s001.pdf]

THE EMBO JOURNAL

DGAT1 activity synchronises with mitophagy to protect cells from metabolic rewiring by iron depletion

Maeve Long, Alvaro Sanchez-Martinez, Marianna Longo, Fumi Suomi, Hans Stenlund, Annika Johansson, Homa Ehsan, Veijo Salo, Lambert Montava-Garriga, Seyedehshima Naddafi, Elina Ikonen, Ian Ganley, Alexander Whitworth, and Thomas McWilliams
DOI: 10.15252/embj.2021109390

Corresponding author(s): Thomas McWilliams (thomas.mcwilliams@helsinki.fi)

Review Timeline:

Submission Date:	5th Aug 21
Editorial Decision:	18th Aug 21
Revision Received:	17th Jan 22
Editorial Decision:	8th Feb 22
Revision Received:	24th Feb 22
Accepted:	25th Feb 22

Editor: Daniel Klimmeck

Transaction Report:

(Note: With the exception of the correction of typographical or spelling errors that could be a source of ambiguity, letters and reports are not edited. Depending on transfer agreements, referee reports obtained elsewhere may or may not be included in this compilation. Referee reports are anonymous unless the Referee chooses to sign their reports.)

1st Editorial Decision 18th Aug 2021

Dear Dr McWilliams,

Thank you for submitting your work for consideration by the EMBO Journal and transferring your manuscript from Review Commons, now listed as EMBOJ-2021-109390. My apologies for getting back to you with unusual protraction due to the high current load of submissions to the journal.

We have now carefully assessed your manuscript together with the referee reports and your point-by-point response to their concerns. I am happy to say that we find the results to be of interest for the EMBO Journal, and thus are positive to have a revised study re-evaluated by the referees.

Given the referees' positive recommendations and based on your detailed response, I would thus like to invite you to submit a revised version of the manuscript, addressing the issues raised. I should add that it is EMBO Journal policy to allow only a single round of revision, and acceptance of your manuscript will therefore depend on the completeness of your responses in this revised version.

We generally allow three months as standard revision time. As a matter of policy, competing manuscripts published during this period will not negatively impact on our assessment of the conceptual advance presented by your study. However, we request that you contact the editor as soon as possible upon publication of any related work, to discuss how to proceed. Should you foresee a problem in meeting this three-month deadline, please let us know in advance and we may be able to grant an extension.

When submitting your revised manuscript, please carefully review the instructions below.

Please feel free to approach me any time should you have additional questions related to this.

Thank you for the opportunity to consider your work for publication.

I look forward to your revision.

Kind regards,

Daniel Klimmeck

Daniel Klimmeck, PhD
Senior Editor
The EMBO Journal

Instruction for the preparation of your revised manuscript:

1) a .docx formatted version of the manuscript text (including legends for main figures, EV figures and tables). Please make sure that the changes are highlighted to be clearly visible.

2) individual production quality figure files as .eps, .tif, .jpg (one file per figure).

3) a .docx formatted letter INCLUDING the reviewers' reports and your detailed point-by-point response to their comments. As part of the EMBO Press transparent editorial process, the point-by-point response is part of the Review Process File (RPF), which will be published alongside your paper.

4) a complete author checklist, which you can download from our author guidelines (https://wol-prod-cdn.literatumonline.com/pb-assets/embo-site/Author Checklist%20-%20EMBO%20J-1561436015657.xlsx). Please insert information in the checklist that is also reflected in the manuscript. The completed author checklist will also be part of the RPF.

5) Please note that all corresponding authors are required to supply an ORCID ID for their name upon submission of a revised manuscript.

6) It is mandatory to include a 'Data Availability' section after the Materials and Methods. Before submitting your revision, primary datasets produced in this study need to be deposited in an appropriate public database, and the accession numbers and

database listed under 'Data Availability'. Please remember to provide a reviewer password if the datasets are not yet public (see https://www.embopress.org/page/journal/14602075/authorguide#datadeposition).
In case you have no data that requires deposition in a public database, please state so in this section. Note that the Data Availability Section is restricted to new primary data that are part of this study.
*** Note - All links should resolve to a page where the data can be accessed. ***

7) Our journal encourages inclusion of *data citations in the reference list* to directly cite datasets that were re-used and obtained from public databases. Data citations in the article text are distinct from normal bibliographical citations and should directly link to the database records from which the data can be accessed. In the main text, data citations are formatted as follows: "Data ref: Smith et al, 2001" or "Data ref: NCBI Sequence Read Archive PRJNA342805, 2017". In the Reference list, data citations must be labeled with "[DATASET]". A data reference must provide the database name, accession number/identifiers and a resolvable link to the landing page from which the data can be accessed at the end of the reference. Further instructions are available at .

8) We would also encourage you to include the source data for figure panels that show essential data. Numerical data can be provided as individual .xls or .csv files (including a tab describing the data). For 'blots' or microscopy, uncropped images should be submitted (using a zip archive or a single pdf per main figure if multiple images need to be supplied for one panel). Additional information on source data and instruction on how to label the files are available at .

9) We replaced Supplementary Information with Expanded View (EV) Figures and Tables that are collapsible/expandable online (see examples in https://www.embopress.org/doi/10.15252/embj.201695874). A maximum of 5 EV Figures can be typeset. EV Figures should be cited as 'Figure EV1, Figure EV2" etc. in the text and their respective legends should be included in the main text after the legends of regular figures.

- For the figures that you do NOT wish to display as Expanded View figures, they should be bundled together with their legends in a single PDF file called *Appendix*, which should start with a short Table of Content. Appendix figures should be referred to in the main text as: "Appendix Figure S1, Appendix Figure S2" etc. See detailed instructions regarding expanded view here: .

- Additional Tables/Datasets should be labelled and referred to as Table EV1, Dataset EV1, etc. Legends have to be provided in a separate tab in case of .xls files. Alternatively, the legend can be supplied as a separate text file (README) and zipped together with the Table/Dataset file.

10) When assembling figures, please refer to our figure preparation guideline in order to ensure proper formatting and readability in print as well as on screen:
http://bit.ly/EMBOPressFigurePreparationGuideline

Please remember: Digital image enhancement is acceptable practice, as long as it accurately represents the original data and conforms to community standards. If a figure has been subjected to significant electronic manipulation, this must be noted in the figure legend or in the 'Materials and Methods' section. The editors reserve the right to request original versions of figures and the original images that were used to assemble the figure.

11) For data quantification: please specify the name of the statistical test used to generate error bars and P values, the number (n) of independent experiments (specify technical or biological replicates) underlying each data point and the test used to calculate p-values in each figure legend. The figure legends should contain a basic description of n, P and the test applied. Graphs must include a description of the bars and the error bars (s.d., s.e.m.).

Further information is available in our Guide to Authors: https://www.embopress.org/page/journal/14602075/authorguide

Revision to The EMBO Journal should be submitted online within 90 days, unless an extension has been requested and approved by the editor; please click on the link below to submit the revision online before 16th Nov 2021:

Link Not Available

Author response

We thank the Editor and the Reviewers for all their work in assessing our manuscript and helping us improve it. We are particularly grateful for their encouraging feedback and recognition of its quality, novelty, and impact. We believe all the comments were fair and reasonable, and as indicated below, we have tried to address these and capture all concerns as best as possible within the current constraints of the global pandemic. We have updated some figures to incorporate new data, and the manuscript contains three additional main figures, plus two extra supplemental figures (referred to as 'Expanded View' - changes are summarised in the associated table). Although a complete clarification of the mechanism is not possible in this manuscript, we think we have accomplished most of what the reviewers have asked us to verify. Importantly, the manuscript is greatly strengthened by several elements.

First, we were able to uncouple LD biogenesis from the autophagy machinery using autophagy-deficient cells to demonstrate DFP robustly induces LD biogenesis in the absence of autophagy. Second, our mitophagy findings are rigorously consolidated by their independent validation in two independent, unrelated laboratories, which employed differential strategies and systems to demonstrate that loss of DGAT1/2 signalling restricts mitophagy (new Figure 6d-e). Third, we show that NIX/BNIP3L-dependent priming and autophagy initiation are not altered by DGAT1/2 inhibition, but instead, we found that loss of LD biogenesis impairs lysosomal positioning and compounds the lipid imbalances upon iron depletion (new Figure 6f-j, Appendix Figure S2). Finally, we performed genetic experiments that authenticated the physiological significance of our mitophagy findings using an *in vivo* reporter model (using a distinct reporter strategy) (new Figure 7), which also revealed an unexpected motor phenotype. We have included this compelling data because it consolidates these exciting new links between lipid homeostasis, metabolism, and mitophagy. We feel our findings are timely and expect they will be of broad significance to researchers in multiple fields who are increasingly turning their attention to mitophagy and metabolism.

Detailed description of changes made in response to referees – summary table - Long *et al*.

Please note: "Supplemental figures" now listed in "Expanded View" and "Appendix" format (EMBO guidelines)

Original Submission	Revised Submission	Amendments
Figure 4	Additional panel "4f"	**New data panel**
-	**Figure 5**	**New primary figure**
-	Figure 5a	**New data panel**
-	Figure 5b	**New data panel**
-	Figure 5c	**New data panel**
-	Figure 5d	**New data panel**
-	Figure 5e	**New data panel**
Figure 5	Designated **new Figure 6**	
-	Figure 6a	New explanatory graphic
-	Figure 6d	**New data panel**
-	Figure 6e	**New data panel**
-	Figure 6f	**New data panel**
-	Figure 6g	**New data panel**
-	Figure 6h	**New data panel**
-	Figure 6i	**New data panel**
-	Figure 6j	**New data panel**
Figure 6	Designated **new Expanded View Figure EV5**	-
Figure 6d	-	Deleted line schematic
-	**Figure 7**	**New primary figure**
-	Figure 7a	**New data panel**
-	Figure 7b	New explanatory graphic
-	Figure 7c	**New data panel**
-	Figure 7d	**New data panel**
-	**Figure 8**	**New primary figure (schematic)**
	**Expanded View Figure EV4**	**New Expanded View figure**
	Figure EV4a	**New data panel**
	Figure EV 4b	**New data panel**
	Figure EV 4c	**New data panel**
Figure S4	Designated **new Appendix Figure S1**	
	Appendix Figure S1b	**New data panel**
	**Expanded View Figure EV5**	
	Figure EV5a	**New data panel**
	**Appendix Figure S2**	**New supplemental figure**
	Figure S2a	**New data panel**
	Figure S2b	**New data panel**
	Figure S2c	**New data panel**

Reviewer #1 (Evidence, reproducibility and clarity (Required)):

"The study by Long et al. shows that mitochondrial iron depletion caused by DPE treatment induces an early increase in lipid droplet formation that precedes mitophagy. They found that lipid droplet formation occurs with an increase in the association of mitochondria to lipid droplets, with the expansion of lipid droplets occurring at later times being dependent on DGAT1. Supporting this conclusion, DGAT1 inhibition blocks LD expansion and increases mitochondrial ROS production, decreasing both mitophagy and cell viability. From these data, the authors conclude that DGAT1 activity and lipid metabolism remodeling are required for mitophagy. However, the evidence presented can have alternative interpretations and thus more experiments are required to support the conclusions of the authors:"

Author response

- We thank the reviewer for their considered review. They have raised important points that we have tried to address, and their suggestions have resulted in a more refined mechanistic insight that was lacking from the original manuscript.

"1) DGAT2 might be involved in LD biogenesis (occurring the first 7h of DPE treatment) and DGAT1 is only involved in the expansion of lipid droplets occurring after 7h of treatment. Authors should perform one experiment in which DGAT1/2 inhibitors are added at the same time or 15 minutes after adding DFE."

Author response

- We performed these experiments as advised, and we also incorporated an extra timepoint in addition to this using high-content confocal microscopy. Briefly, addition of DGAT inhibitors simultaneously with DFP, 15 mins post DFP or 1-hour post-DFP all abolished LD biogenesis. Thus, although LDs manifest by microscopy at the 7h timepoint, these experiments show that acute DGAT1 inhibition blocks the effects of iron depletion on LD biogenesis. Data are shown below and incorporated as a new panel - in the main data, as **Figure 4F (please see below):**

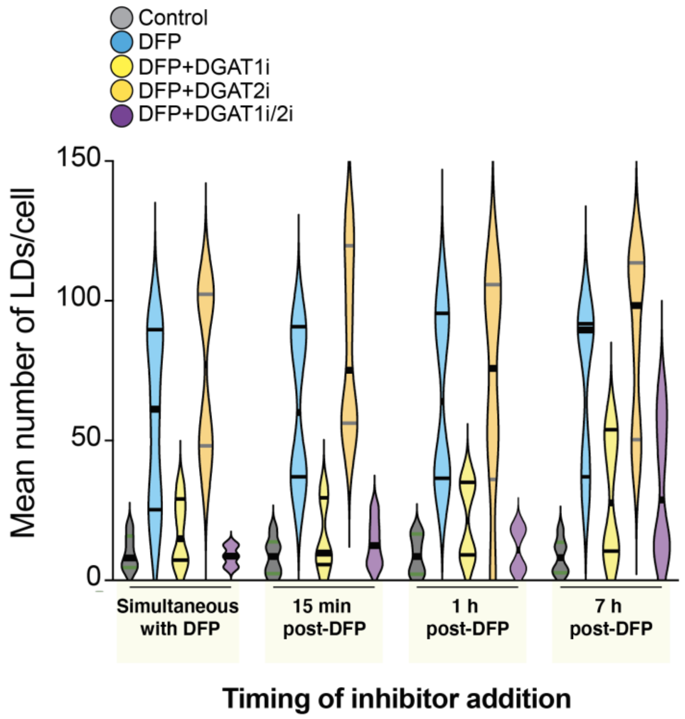

New Figure 4F

- <u>Brief description:</u> Iron depletion and simultaneous inhibition of DGAT1 or DGAT1/2 activity abolishes LD biogenesis in human ARPE-19 cells, equivalent to inhibitor addition post-DFP. Data derived from high content confocal microscopy; *n*=2. An expanded figure legend is available in the revised text.

"2) It is a possibility that an increase non-esterified/free fatty acids caused by DGAT1 inhibition is responsible for the reduction in mitophagy, by impairing lysosomal function (Zi et al., Hepatology 2008). To discard this possibility and support the authors' conclusions, lysosomal pH should be measured. If the conclusion of the authors is correct (TG synthesis mediated by DGAT1 needed for mitophagy), lysosomal acidity and autophagic flux should not be impaired by DGAT1 inhibition."

Author response

- We thank the reviewer for raising this point on mechanism, which we are committed to resolving. The suggestion about lysosomal homeostasis proved to be very incisive and this prediction has emerged to be more than a possibility! Accordingly, we have revised our interpretation of the data considering the new findings described below.
- Briefly, our experiments revealed significant alterations in the spatial disposition of lysosomes upon inhibition of LD biogenesis during mitophagy induction. Live cell imaging revealed altered lysosomal positioning upon inhibition of LD biogenesis (new imaging data included).
- We tried very hard using several methods to assess lysosomal pH, and the results were variable – sometimes there appeared to be a clear effect of DGAT1i/2i inhibition on lysosomal pH, other times this was not the case (we used dual dextran labelling, DQ-BSA and cathepsin activity assays). In the interests of rigour and precision, we conclude that, in our hands, the dynamic range of the assays we used were not sensitive enough to consistently detect reproducible changes. We attribute this to the current experimental setup and predict that longer timepoints or different setups may be needed, however this would exceed the window of interest to our studies. **Regardless, the selective effects of DGAT1/2 inhibition on lysosomal positioning and displacement were extremely consistent**. Quantification of confocal images revealed that the absence of LD biogenesis is associated with lysosomal displacement i.e., more peripheral lysosomes. The position of lysosomes has emerged as a key feature of their functional integrity and is tightly related to pH (Johnson *et al*., 2016 Journal of Cell Biology, PMID: 26975849). Moreover, lysosome positioning and distribution has previously been implicated in the progression of non-selective macroautophagy (Korolchuk *et al*., 2011 Nature Cell Biology PMID: 21394080). Given findings from converging experiments, we predict this perturbation in lysosomal integrity accounts for the defect in mitophagy.The spatial disposition of mitolysosomes in reporter cells was not overtly affected by DGAT1/2 inhibition, suggesting a subset of lysosomes may be compromised by lipid imbalance prior to fusion. However, the dynamic interplay and precise mechanism by which DGAT1 inactivation impairs lysosomal homeostasis remains to be clarified. We have updated the manuscript to reflect this revised interpretation and thank the reviewer again for their excellent suggestion. Data are now included as **Figure 6f-j, also shown below:**

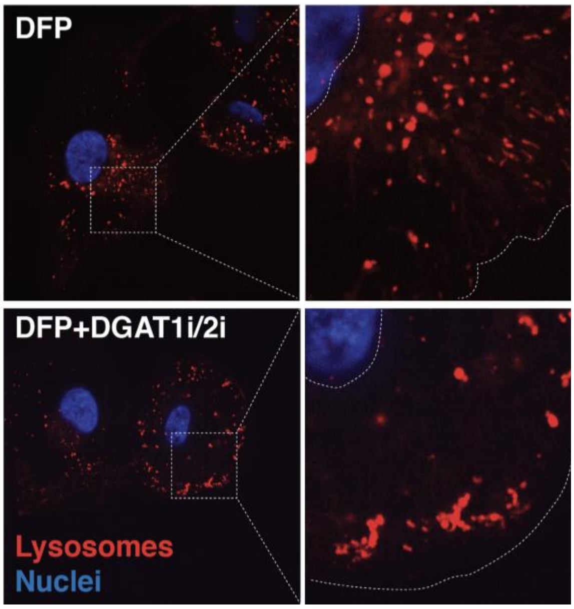

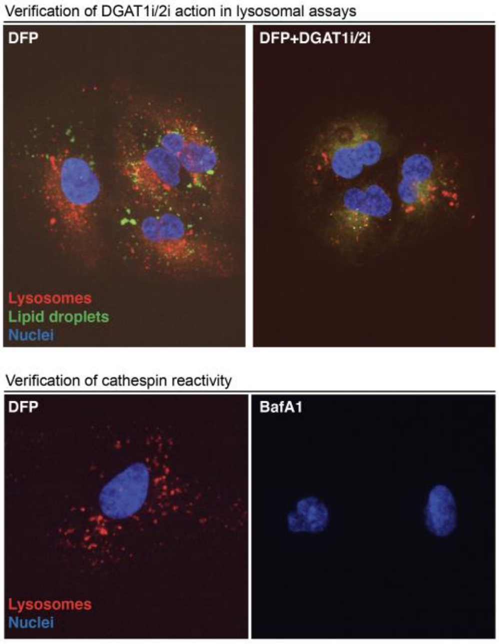

<u>Representative images from optimisation experiments</u>: showing effect of DGAT1i/2i treatment on LD biogenesis (green) in experiments monitoring lysosomal homeostasis (red). MR-cathespin-reactivity (red) was abolished upon treatment with the lysosomal V_1V_0 ATPase inhibitor Bafilomycin A1.

New Figure 7g (left panel)

- <u>Brief description:</u> Representative photomicrographs showing loss of LD biogenesis upon mitophagy induction disrupts lysosomal homeostasis in human ARPE-19 cells. Lysosomes were illuminated using the Cathespin-reactive substrate dye Magic Red (as described in Bright *et al*., 2016 *Current Biology*; PMID: 27498570) and visualised using live cell spinning disk confocal microscopy. Scale bar = 5 microns. Lysosomes within the inhibitor treated cells were often distinguished by their more peripheral or altered positions.

Continued -

- When considered with new data from additional experiments suggested by other reviewers on mitophagy signalling (no defect in ULK1, WIPI2 puncta formation or in the induction of NIX/BNIP3L signalling **– shown below and included as a new panel in Figure 6**), we conclude that the mitophagy defect lies at the level of the lysosomal dysfunction from abarrent lipid metabolism (non-esterified fatty acids) which is exacerbated by impaired LD biogenesis. Accordingly, we have tapered our discussion regarding ROS.
- As the reviewer helpfully highlighted, there is precedence for lysosomal dysfunction upon loss of lipid homeostasis. In our model, iron depletion already rewires metabolism, inducing changes in lipid homeostasis that are normally neutralised by DGAT1-dependent LD biosynthesis. We predict these effects are compounded by loss of LD biogenesis, compromising lysosomal homeostasis and restricting the efficiency of mitophagy.

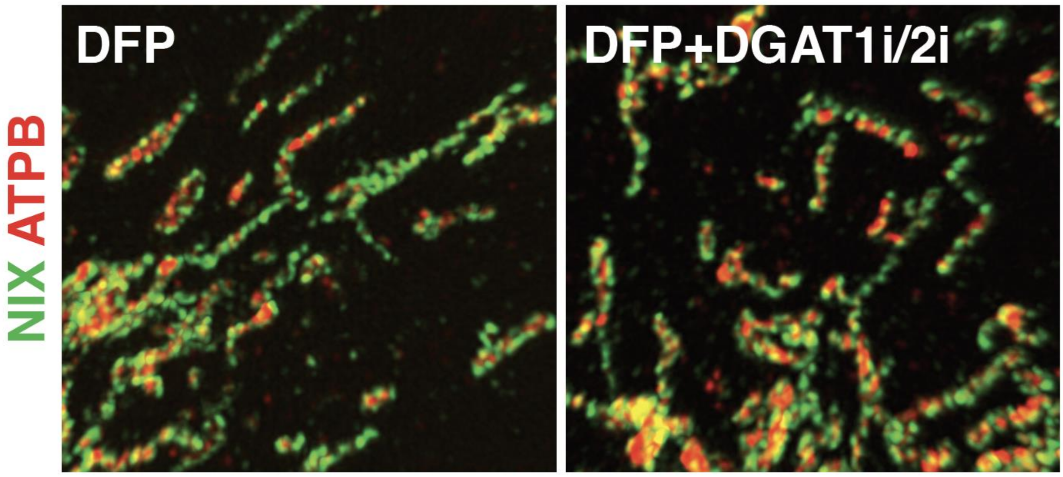

New Figure 7f

- Brief description: Representative photomicrographs demonstrating that impaired LD biogenesis does not disrupt the early stages of mitophagy – specifically, recognition and priming of recognition or mitochondrial priming of damaged mitochondria for elimination by NIX/BNIP3. High resolution confocal microscopy revealed no alteration in the distribution and targeting of endogenous NIX (green) to mitochondria (ATP5B; red) between DFP and DFP+DGAT1i/2i treated conditions. Scale bar = 2 microns.

New Figure 7h-j

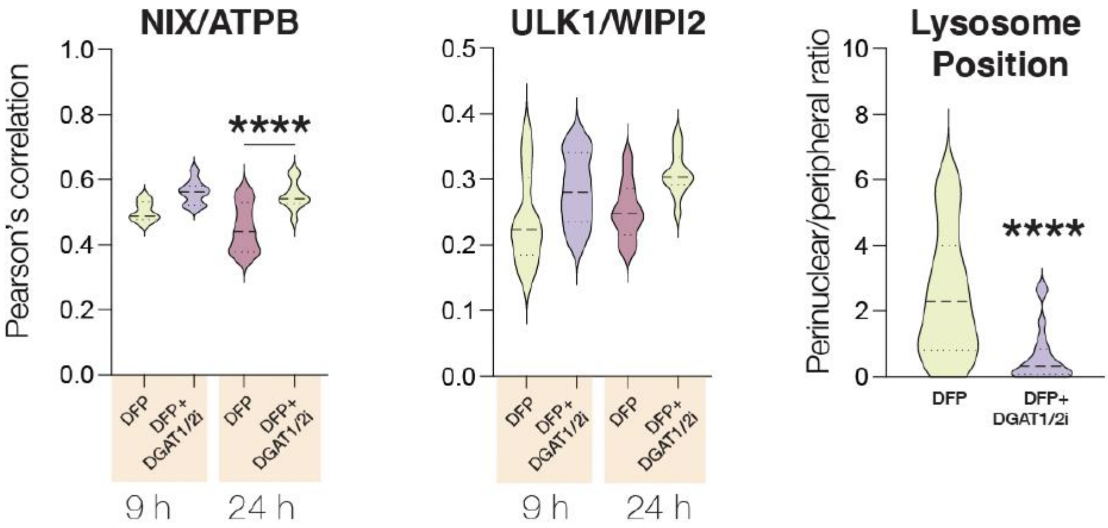

- Brief description: Quantitation of new imaging experiments; No reduction is observed in the selective autophagy marker NIX or in the levels of WIPI2/ULK1-positive puncta upon mitophagy induction and blockade of LD biosynthesis (we assayed levels at the onset of NIX stabilisation, ULK activity and at the peak of mitolysosome formation). Conversely, DGAT1/2 inhibition during DFP-induced mitophagy disrupts lysosomal positioning. In

"3) Authors' attribute the change in lipid metabolism to mitochondrial dysfunction induced by iron depletion, via elevated mitochondrial ROS production. However, the early lipidome profile does not recapitulate defects in mitochondrial beta-oxidation resulting from electron

transport chain dysfunction (i.e. similar levels of acylcarnitines). In addition, mitochondrial ROS were only measured after 24h of DPE treatment, despite ROS are proposed to be an early effector of DPE actions. Furthermore, mitochondria associated to lipid droplets have higher respiratory function (Bosch et al., Science 2020). To support the authors' model, mitochondrial respiratory capacity, fatty acid oxidation and ROS should be measured at early time points of DPE treatment (before 7h) and determine whether mitochondria antioxidants can prevent lipid formation in DPE treated cells. Without these experiments, another possibility could be that, rather than mitochondrial dysfunction and elevated ROS as a primary event resulting from DPE treatment, iron depletion might be altering phospholipid synthesis or other lipid specifies first in a way that fatty acid precursors need to be re-routed to neutral triglycerides within lipid droplets. This selective change in lipid metabolism could still lead to more non-esterified fatty acid-induced toxicity to mitochondria and lysosomes, exacerbated by DGAT1 inhibition."

Author response

- These are great points, many thanks. We have revised our model based on the Reviewer's previously suggested experiments investigating lysosomal homeostasis – and have reformulated our focus on mitochondrial ROS (we have moved this data to **Figure EV5c**).
- We apologise for the lack of clarity in the original submission: indeed, our metabolomics profiling reveals highly specific and significant changes in subsets of short chain acylcarnitine species at early timepoints (and these are in **Figure EV1a-d, EV1g, and extensively detailed in Figure EV2**). At later timepoints, we also observed reduced levels of *CPT1* and *CPT2* mRNA transcripts upon DFP treatment (**Figure EV3b**). Although these findings are not direct readouts of beta-oxidation – they represent convergent lines of evidence that suggest mitochondrial lipid handling in DFP-treated cells is likely compromised. The ratio of short chain to long chain fatty acids is markedly altered upon iron depletion (**Figure EV1g**)., and loss of LD biogenesis exacerbates the consequences of this metabolic rewiring (**Figure EV5b, EV5d and Appendix Figure S2**).
- We agree regarding the differential bioenergetic properties of peri-droplet mitochondria, which would be certainly interesting to examine in the context of our findings and have incorporated this perspective into the revised discussion. We fully agree that it would be interesting to measure these bioenergetic parameters, but this is not currently feasible for us. Indeed, beta-oxidation is not straightforward to measure, and available assays to directly monitor reliable changes are notoriously challenging, due to their variability and low dynamic range. Given the number of variables at play in our system (iron depletion, timing of LD inhibition, subsets of mitochondria and LDs) it is beyond our current capabilities and the scope of the present manuscript.
- Complementing this revised focus, our *in vitro* data is strongly complimented by **exciting *in vivo* data that demonstrates the physiological significance of DGAT1 for basal mitophagy in tissues.** This compelling phenotype using a distinct mitophagy reporter verifies the basis of our *in vitro* data and consolidates the link between lipid metabolism and physiological mitophagy as a new **Figure 7 (shown below). Tissue-specific depletion of DGAT1 impairs *in vivo* mitophagy in two RNAi lines.** Intriguingly, the profile of mitolysosomes in these mutants also exhibit some morphological differences compared to control animals. Accordingly, these exciting phenotypic findings have

reorientated our perspective on mitochondrial ROS, which we have now moved to Expanded View (Figure EV5).

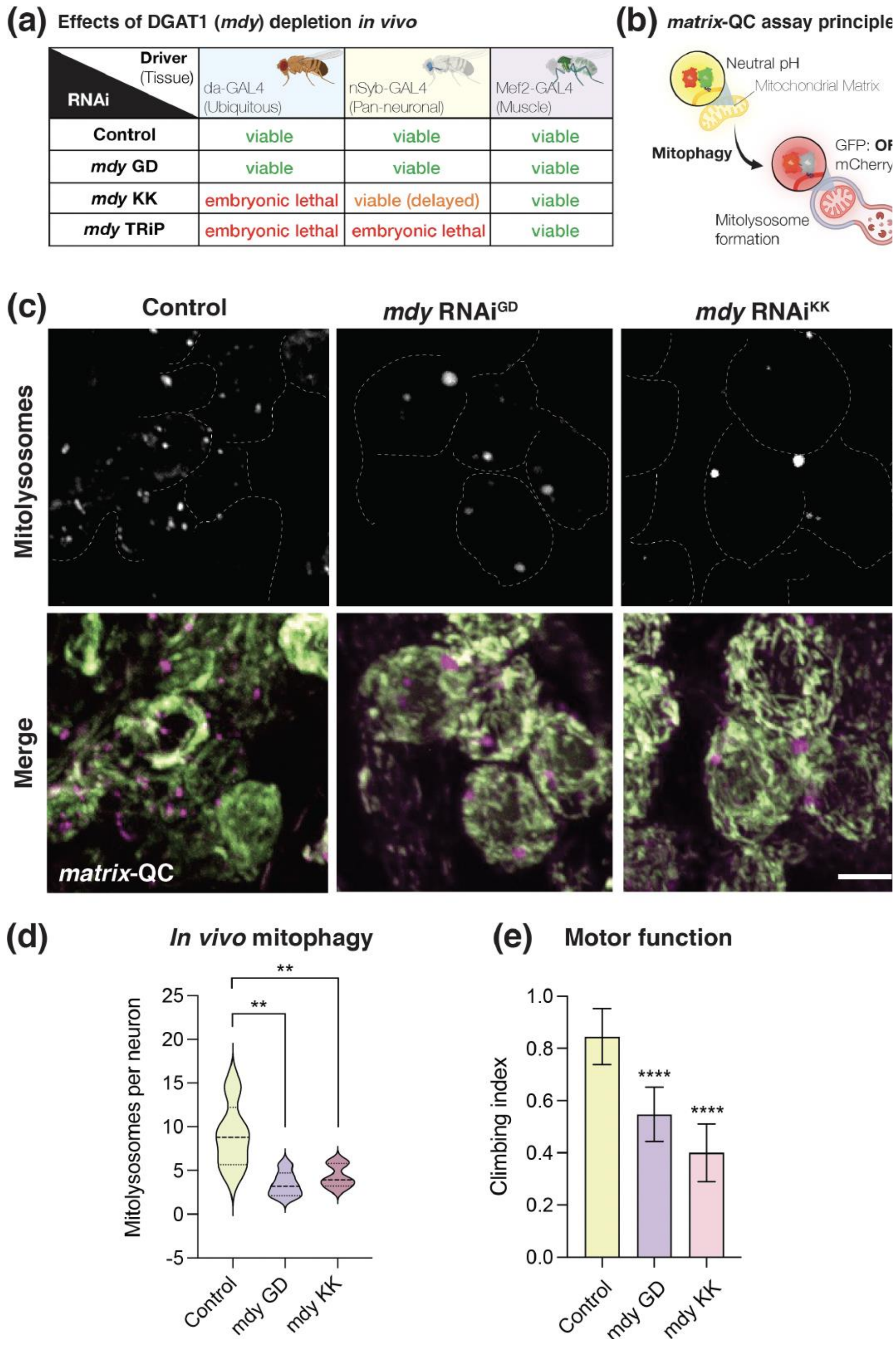

New Figure 7

- Brief description: We assessed the physiological significance of DGAT1 for mitophagy *in vivo* using genetic experiments in reporter animals. We accomplished this using pan-neuronal RNAi-mediated depletion of DGAT1 (*mdy*) in *matrix*-QC reporter flies (mCherry-

GFP-COXVIII). Quantitation of confocal images from two distinct mutants revealed a robust impairment in mitophagy levels *in vivo* (*n*=6 animals per condition), in addition to motor dysfunction, as revealed by the climbing assay. Figure detailed extensively in the main text.

Reviewer #1 (Significance (Required)):

"The finding that changes in lipid metabolism induced by iron depletion occur very early and can even precede changes in mitochondrial function and mitophagy is highly significant. It points to mitochondrial iron playing an important role in lipid biosynthesis.

The audience can be broad, from scientists interested in metabolism to the ones interested in regulatory mechanisms of mitophagy, as well as scientists interested in mechanisms of lipid synthesis regulation."

Author response

- We thank the Reviewer for their considered and constructive evaluation, and for recognising the impact and novelty of our work. Their great suggestions have enabled us to both refine and strengthen our study.

Reviewer #2 (Evidence, reproducibility and clarity (Required)):

"In this study, the authors utilise metabolomics to assess the temporal metabolic profile of cells treated with the iron chelator DFP. The authors identify altered lipid homeostasis which was proposed to be upstream of mitophagy initiation (although what the authors refer to as mitophagy initiation requires clarification, discussed further below). The changes in lipid homeostasis were correlated with increased lipid droplet biogenesis, which has previously been reported for DFP treated cells. The lipid droplets appear to have increased contacts with mitochondria, and through the inhibition of DGAT1 (a lipid droplet biogenesis factor), the authors shows that lipid droplet biogenesis is important for DFP induced mitophagy and for limiting mitoROS production. The work is based on a very strong foundation with clear and robust data, but would benefit with further experiments to help strengthen the main conclusions and to better understand the role of lipid droplets in mitophagy."

Author response

- We thank the Reviewer for their systematic analysis and for highlighting important points that helped introduce greater precision and strengthen our main conclusions. We are grateful for the acknowledgement regarding the quality of our work.

Major Comments:

"1. Figure 3: There are some questions around what is meant by the authors when they refer to the formation of red mitoQC foci as the onset of mitophagy. The delivery of mitochondria to lysosomes is a late-stage event of mitophagy. The onset of mitophagy is more likely to be represented by early autophagy markers of ULK1 complex subunits, while the early-mid stage is represented by PI3P binding proteins including WIPI2 and DFCP1. It would therefore be important to stain and quantitate early-stage markers in relation to lipid droplet formation. The onset of mitophagy needs to be clearly defined because it can be differentially interpreted by readers. It is fine if the authors choose to separate mitophagy initiation from delivery to lysosomes, but this needs to be experimentally determined and made clear in the text."

Author response

- We thank the Reviewer for this comment. We agree that the paper would benefit from more clarity and precision with respect to reading about mitophagy stages, and to also mechanistically uncouple selective autophagy from LD biogenesis.
- Regarding the timing of DFP-induced mitophagy: this has been resolved in previous published experiments by the Ganley lab (Please see Zhao *et al*., 2020; PMID: 32420530 – Figure 1). In this paper, prolonged DFP treatment induces the expression of selective autophagy markers, NIX and BNIP3L, as monitored by immunoblotting in time course experiments. NIX and BNIP3L start to accumulate around 4 hours post-DFP treatment, with significant stabilisation from 8 hours of DFP treatment. Accordingly, we have updated the text to reflect the nuances of these monitoring approaches more precisely.
- For the reviewer's interest, we were also able to experimentally resolve endogenous NIX decorating damaged mitochondria in DFP-treated cells using super-resolution microscopy at 8 hours (see response to point regarding Figure 5, below), even though we do not detect many mitolysosomes in the reporter cell lines at this timepoint. Clearly,

sensing and priming of damaged mitochondria is distinct from the end point readout that mitophagy reporters provide, and we have made this point more explicit in the revised text.

“2. Figure 3G: How do the 60% levels of mitochondria bordering mitochondria compare to the ratio in the control? Is it significantly different? This would help to make a stronger case that contacts between mitochondria and lipid droplets are indeed increased upon iron depletion.”

Author response

- We have quantified differences between the ratios and confirm the increase is significantly different (****P*<0.001) We have updated the text to incorporate this metric.

“3. Figure 5: To ensure that the DGAT1 inhibitor does not have any off-target effects on mitophagy, can the authors utilise DGAT1 KO or DGAT1 RNAi along with appropriate DGAT1 rescue controls?”

Author response

- We thank the reviewer for raising this important point. We conducted equivalent experiments using DFP in cells treated with scrambled or siRNAs to DGAT1 and monitored LD biogenesis (we confirmed efficiency of knockdown by qPCR). The response is identical to chemical inhibition of DGAT1 signalling and these new data are now included as **Expanded View Figure EV4c (below)**. In terms of these inhibitors, they have been used in many other studies, and in our hands, also robustly inhibit DFP-induced LD biogenesis in two additional cell types: U2-OS human osteosarcoma cells as well as human patient fibroblasts.

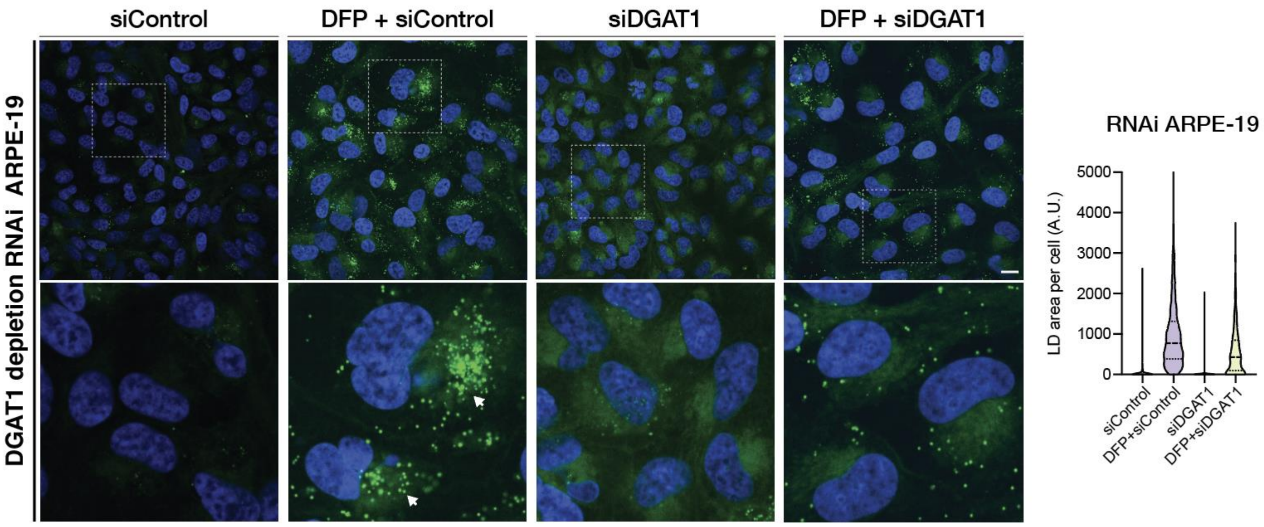

Continued -

- Related to the Reviewer’s point - we performed a genetic experiment *in vivo*, using depletion of DGAT1 in a distinct mitophagy reporter in an animal model (*Drosophila - matrix*-QC: mCherry-GFP-COXVIII). We thank the reviewer for the suggestion of a genetic experiment, as it has enabled to authenticate the physiological relevance of

DGAT1 for mitophagy *in vivo*. Pan-neuronal depletion of DGAT1 (*mdy*) markedly impaired basal mitophagy in brain tissue, accompanied by a compelling locomotor phenotype. Taken together, we have used multiple methods to verify the tools used and phenotypes observed in the study and our revised manuscript robustly implicates lipid homeostasis in physiological mitophagy. We have included these new findings **as new panels in a new main Figure 7** (**shown below**).

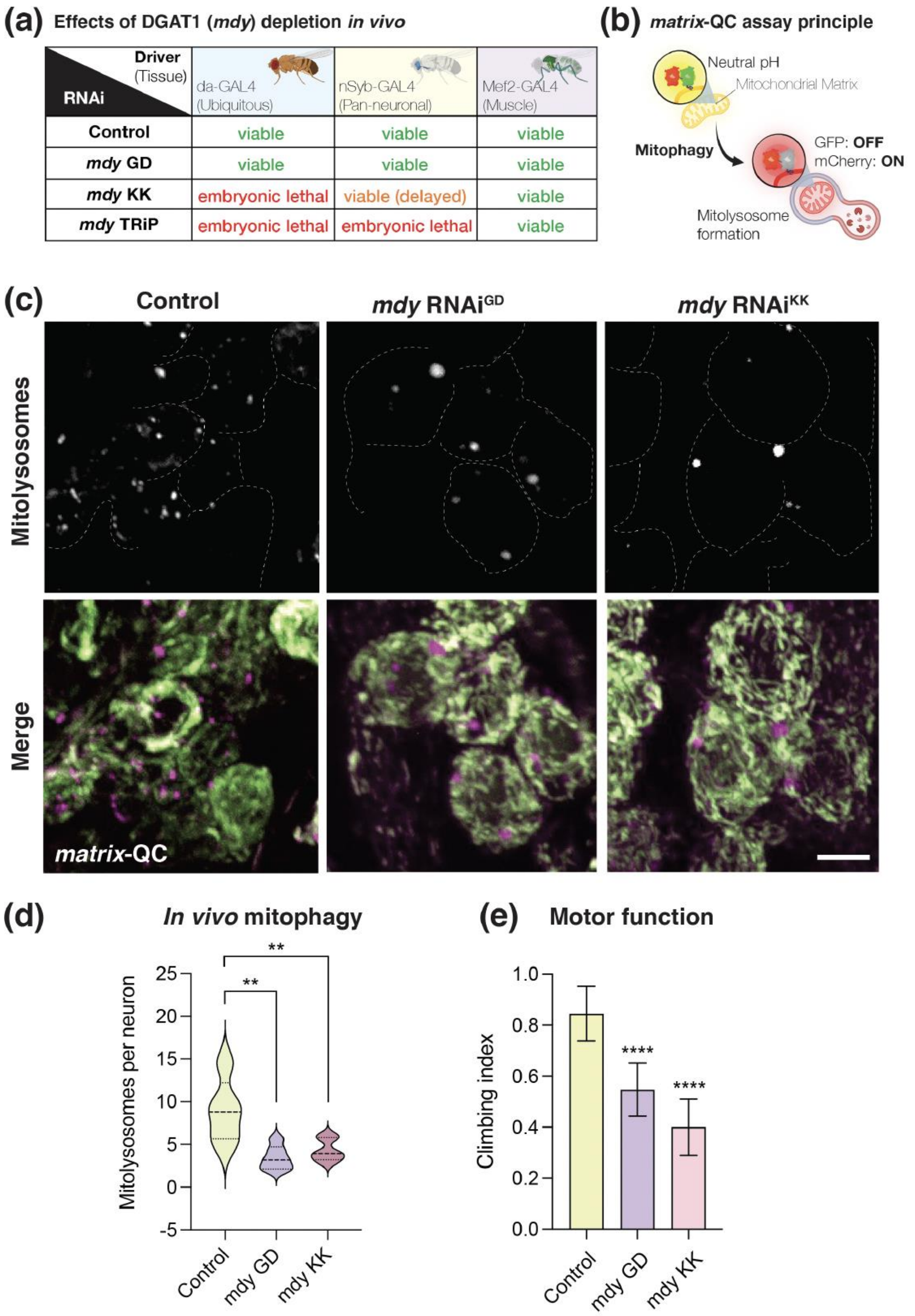

Continued from page 10 - **New Figure 7:**

Brief description: We assessed the physiological significance of DGAT1 for mitophagy *in vivo* using reporter animals. We accomplished this using pan-neuronal RNAi-mediated depletion of DGAT1 (*mdy*) in *matrix*-QC reporter flies (mCherry-GFP-COXVIII). Quantitation of confocal images from two distinct mutants revealed a robust impairment in mitophagy levels *in vivo* (*n*=6 animals per condition), in addition to motor dysfunction, as revealed by the climbing assay.

"4. Figure 5: Does DGAT1 inhibition reduce starvation induced autophagy or is it specific to DFP induced mitophagy? This would help clarify whether the induction of lipid droplets is related to metabolic rewiring to support mitophagy or if there is a general role for lipid droplets in supporting autophagy."

Author response

- We thank the reviewer for raising this important point. The functional role of DGAT1-dependent LD biogenesis in starvation-induced macroautophagy was addressed in a landmark study by the Olzmann Lab at Berkeley (Nyugen et al., 2017). The authors found no effect of DGAT1 depletion or inhibition on the progression of macroautophagy. With this in mind, we speculate there are likely context-specific roles for LD subsets during distinct metabolic states where different forms of selective autophagy are engaged. A unifying theme from our selective autophagy study and the Olzmann paper is that LDs play a cytoprotective role, buffering cells from toxic metabolites that would otherwise lead to decreased viability – presumably through impaired mitochondrial and lysosomal function. We have updated the text to make this point more explicit.

"5. Figure 5: Where does the mitophagy defect of DGAT1 inhibition lie? Decreased mitophagy initiation events (e.g. labelled by ULK1 complex subunit foci), decreased autophagosome formation (e.g. LC3 or GABARAP foci, electron microscopy) or decreased autophagosome-lysosome fusion (as was measured by the mito-QC reporter)? This can help clarify the critical stage at which lipid droplet biogenesis is required for mitophagy. Are BNIP3 and NIX induced to the same level in DGAT1 inhibited cells?"

Author response

- We thank the reviewer for these comments and excellent suggestions. Accordingly, we performed immunostaining at acute and late timepoints to assess WIPI2 and ULK1 puncta formation, which are robustly induced upon iron depletion (Zhao *et al*., 2020; PMID: 32420530). Comparative analysis of DFP and DFP+DGAT1i/2i-treated cells revealed no major differences at acute or late timepoints, suggesting that the defect does not lie at the level of phagophore initiation or encapsulation.
- As the reviewer rightly points out, NIX/BNIP3L play critical roles in DFP-induced mitophagy. Thus, we sought to assay NIX induction and distribution along mitochondria using high-resolution confocal microscopy. Inhibition of LD biogenesis did not alter levels of distribution of mitochondrial NIX. In fact, we observed a modest yet statistically significant increase in mitochondrial NIX upon loss of LD biogenesis combined with iron

depletion – which would be consistent with increased mitochondrial damage. These incisive experiments suggested by the reviewer revealed that inhibition of DGAT1-dependent LD biogenesis does not impair the sensing and priming of damaged mitochondria or early autophagy signalling events – suggesting the mitophagy defect is a consequence of a different stage in the pathway. All of this data is shown below and incorporated **as new panels in Figure 6** (**data shown below**).

- The above experiments enabled us to rigorously dissociate metabolic dysfunction from mitophagy initiation. In addition, from experiments suggested by Reviewer 1, we were able to determine that lysosomal homeostasis is disrupted by loss of LD biogenesis. We attribute this to the presence of non-esterified FAs (NEFAs) which are known to affect lysosomal pH and activity (Li et al., 2008; Las et al. 2011; Jaishy et al. 2015; Jaishy and Abel, 2016), and that NEFAs induce cellular dysfunction in the absence of DGAT1 (Listenberger et al. 2003). Differential lipidomic profiles between the DFP ±DGAT1i/2i suggest other lipids associated with lysosomal dysfunction may also be driving this. In the future, it will be interesting to further explore such lipid imbalances and their effects on mitophagy pathways in a variety of different contexts.
- Upon iron depletion, loss of DGAT1/2 signalling induced lysosomal displacement. This is highly interesting as lysosomal position closely reflects their homeostasis and degradative function (pioneering work by the Grinstein lab and others; Johnson et al. 2016). Furthermore, defective lysosomal positioning also arrests macroautophagy (Korolchuk et al. 2011). This suggests that a role of LD biogenesis is to neutralise the lysosomal dysfunction induced by non-esterified FA's, which has also been shown in other publications. Failure to initiate LD biogenesis reduces mitophagy and cellular viability, because DGAT1 offsets aberrant lipid metabolism that would otherwise compromise lysosomal integrity.

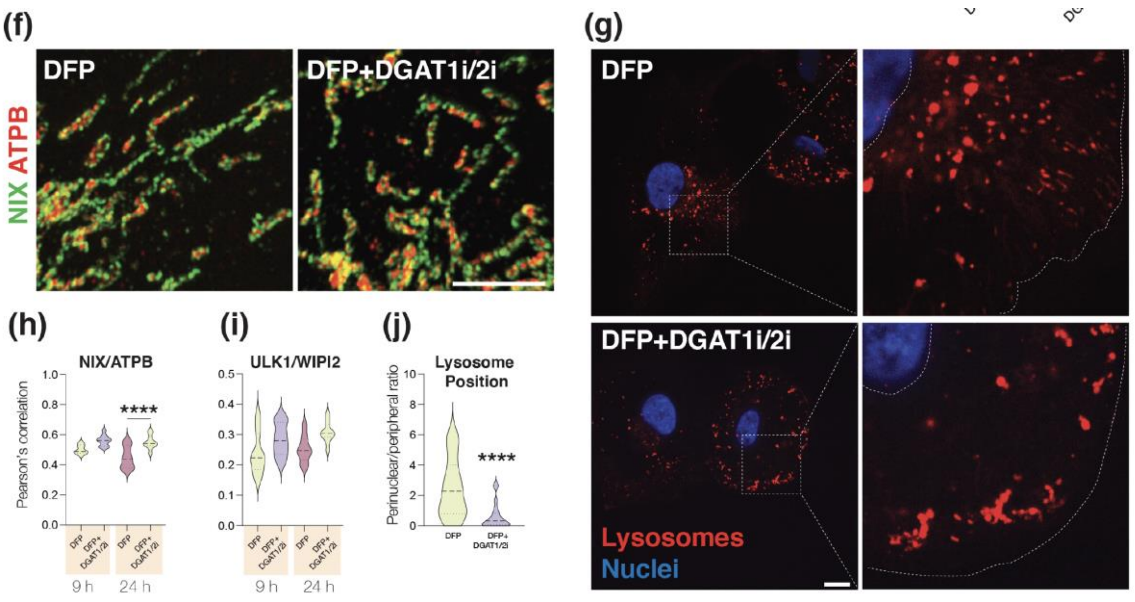

New Figure 6G-K:

<u>Brief description:</u> Loss of LD biosynthesis affects the terminal stages of mitophagy. High resolution confocal imaging of NIX decorating mitochondria at 9 h post DFP treatment, which is not impaired by DGAT1/2 inhibition. The formation or presence of WIPI2/ULK1 positive

puncta at distinct stages is also unaffected. Conversely, lysosomal positioning is disrupted by DGAT1/2 inhibition, as revealed by live cell confocal imaging of cathepsin-reactive lysosomes.

"6. Figure 6: It would be beneficial to clarify the hypothesis behind how DGAT1 regulated lipid droplet biosynthesis protects against mitoROS. Is it via lipid droplet mediated detoxification or via mitophagy? For example, if mitophagy is inhibited via knockout of an autophagy factor (e.g. FIP200, BNIP3/NIX) is the mitoROS protective effect of lipid droplet biogenesis still present/diminished? If it is diminished, to what degree. This could help clarify to what degree lipid droplets contribute to mitoROS production and cell viability via mitophagy vs via a direct role."

Author response

- We thank the reviewer for this great point. In terms of the mtROS angle, we have revised our text in light of recent findings above and have reformulated the manuscript to reflect the new data on lysosomal homeostasis, in addition to our genetic experiments which provide *in vivo* authentication and physiological relevance. Thus, Figure 6 has been updated and a new Figure 7 has been incorporated, as mentioned above.

- Nevertheless, the Reviewer's comments inspired us to investigate if we could mechanistically uncouple DFP-induced LD-biogenesis from the autophagy machinery. This is an important consideration as macroautophagy is required for LD biogenesis upon starvation – LD biogenesis is impaired in *Atg5* KO cells upon starvation (Rambold et al. 2015; Nguyen et al. 2017). This has not been resolved for iron depletion. Here we used autophagy-deficient *ULK1* KO cells generated by CRISPR-Cas9 and incorporated rescue controls by reintroduction of FLAG-ULK1. DFP readily induced DGAT1-dependent LD biogenesis in the absence of ULK1 (a master regulator of autophagy) and this was unaffected by the reintroduction of FLAG-ULK1. We also verified that these cells are not competent for autophagy signalling/ mitophagy unless re-complimented by FLAG-ULK1. Together, these findings verify that the initiation of these pathways is distinct, yet their synergy is required for cell protection. These data are included as a **new Figure 5** and shown below (page 14 of this point-by-point response):

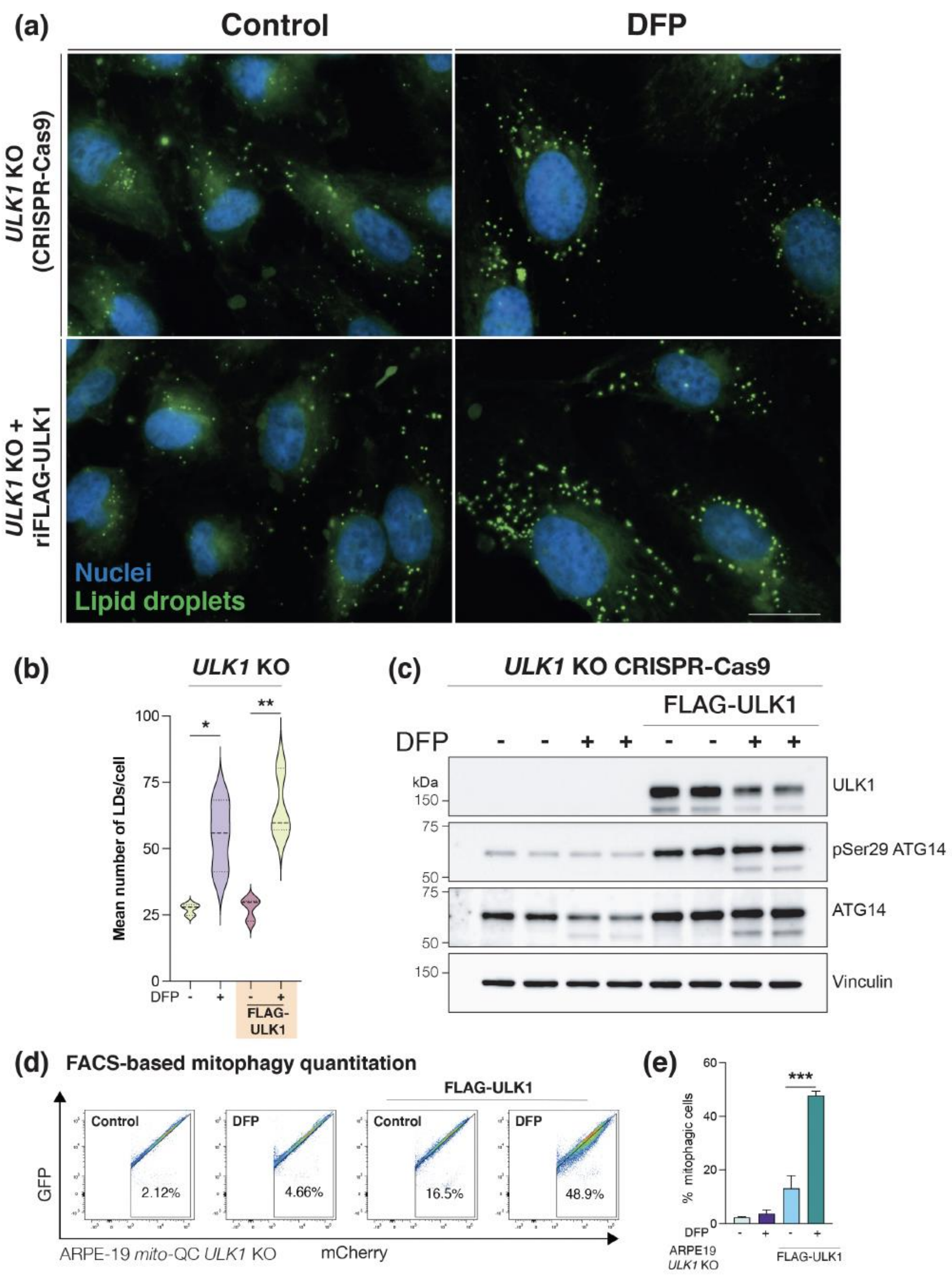

New Figure 5:

<u>Brief description:</u> We uncoupled DFP-induced LD biosynthesis from the autophagy machinery, using *ULK1* KO cells generated by CRISPR-Cas9. These data demonstrate that the formation of LDs upon iron depletion does not require autophagy signalling, in contrast to published data on starvation-induced LD biosynthesis which requires non-selective macroautophagy to liberate fatty acids. ULK1 KO cells were biochemically verified to be

autophagy-deficient and we also confirmed this for mitophagy by quantitation with the *mito*-QC reporter.

Minor Comments:

“1. Figure 2A and Figure 4C, 4F: What is the reference control for the heatmap values? i.e. which sample are they relative to? It would be beneficial to include this information in the figure legend and in the main text when describing the results.”

Author response

- Thanks for highlighting this. The heatmap has been normalised to the untreated control samples. We have clarified this in the revised manuscript and the corresponding legend.

“2. Figure 2A: It would be helpful to highlight/label the clusters, this will help to clearly highlight clusters changes related to lipid metabolism”

Author response

- We will try to do this in the revised manuscript where possible. If this is not possible on the graph, we will include an additional supplemental graph with a more comprehensive subclass analysis.

“3. Figure 2E-G: Plasmalogen biosynthesis is increased, beta oxidation of long/very long chain fatty acids is decreased. These interesting results point toward changes in peroxisome biology. It would be interesting to assess whether peroxisome positioning relative to mitochondria is altered by DFP induced mitophagy.”

Author response

- We thank the reviewer for this suggestion and share their intrigue. A detailed study of peroxisomal function and biology is beyond the scope of the present submission, but we did perform immunolabelling to PEX19 and PMP70 upon DFP treatment ±DGAT1i/2i inhibition. Representative images of PEX19 are shown below for the reviewer's interest, however we do not include this data in the revised manuscript, because we feel it would complicate the message and disrupt the balance of an already data-rich study. We strongly agree with the Reviewer that it will be exciting to decipher how iron depletion affects peroxisome homeostasis – indeed, several other labs are actively pursuing this.
- Interestingly, increased plasmalogen biosynthesis has been recently associated with autophagosome biogenesis and maintenence (Andrejeva et al. 2020).

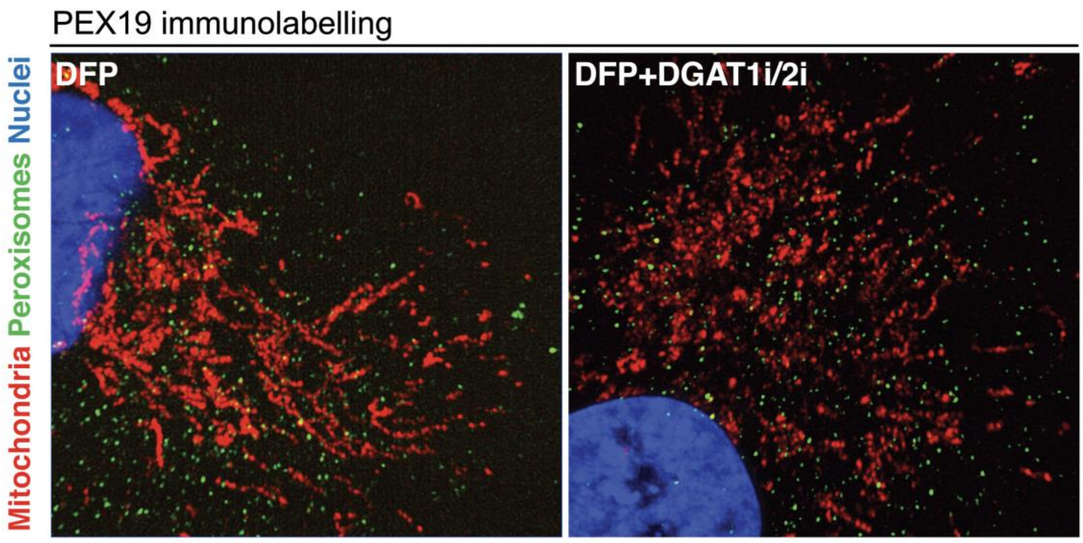

"4. It would be interesting to assess whether the lipid droplet-mitochondria contact events coincide with mitophagy initiation or mitolysosome sites. Together with major comment #5, this experiment could help to provide information on how the lipid droplets are contributing to the mitophagic process."

Author response

- We thank the reviewer for this suggestion. In terms of how LDs are contributing to mitophagy, our data suggests that the LDs do not influence mitochondrial damage sensing or organelle priming, but rather are critical to maintain fatty acid esterification which can compromise lysosomal integrity. Loss of DGAT1 impairs lysosomal homeostasis, leading to reduced levels of mitophagy *in vitro* and *in vivo*.

"5. Figure S4: Quantitation of lipid droplet surface area for the various treatment conditions would help strengthen the author's conclusions."

Author response

- We have done this for **4e and in S4a-c and S5** however differences are also apparent when we quantify LD abundance/frequency per cell.

Reviewer #2 (Significance (Required)):

"Overall, the study reveals new insights into metabolic changes of DFP treated cells, and provides a previously unknown link between lipid droplet biology and mitophagy in the context of DFP treatment. It is a very interesting study, and the discovery of lipid rewiring during DFP treatment (which appears to precede mitophagy) is highly valuable for the mitophagy field (and also the metabolism field). However, the study requires strengthening in terms of depth of knowledge gained in relation to the role of lipid droplets in mitophagy. This is important since it was previously shown that DFP treatment induces lipid droplet formation, DGAT1 is a well characterised lipid droplet biogenesis factor, and lipid droplet formation has previously been shown to contribute to protecting mitochondrial function during starvation autophagy (Nguyen et al (2017) Dev Cell). The major and minor comments suggested to the authors are aimed at helping strengthen the manuscript by digging in a bit deeper into understanding why/how lipid droplets contribute to mitophagy and by clarifying whether the metabolic changes are in fact upstream of early mitophagy initiation signals.

Author response

- We thank the Reviewer for their constructive comments and assistance in helping us strengthen our manuscript by providing more depth to our discovery. We also greatly appreciate their encouraging comments, recognising the broad value this study will provide.

Reviewer #3 (Evidence, reproducibility and clarity (Required)):

Summary:

"Provide a short summary of the findings and key conclusions (including methodology and model system(s) where appropriate).

This study by Long and colleagues aims at unravelling the metabolic events preceding deferiprone (DFP, an iron chelator)-induced PINK1/Parkin-independent mitophagy. To this end, the authors perform temporal metabolomics on DFP-treated mammalian cells. DFP treatment leads to the DGAT1-dependent formation of lipid droplets (LDs) surrounding mitochondria. The authors further propose that chemical inhibition of DGAT1 decreases mitophagy and cell viability through elevated mitochondrial ROS levels. Altogether, these data suggest an interesting link between iron depletion, lipid metabolism and mitophagy."

Author response
We thank the reviewer for their considered critique. They have raised important points and we hoped we have addressed most of these to strengthen the conclusions in our paper.

Major comments:

- Are the key conclusions convincing?

The link between LDs and mitophagy is potentially interesting. However, the paper is quite descriptive and some causal links are missing (E.g. ROS/mitophagy/cell integrity) to firmly establish the mechanism that leads iron chelation to induce mitophagy.

While DGAT1 and 1/2 inhibition completely inhibit the formation of LDs, DGAT1 and 1/2 inhibition only lead to a modest reduction of mitophagy, suggesting that the LDs aren't required for mitophagy. This should be at the very least discussed in the manuscript.

Author response
We thank the reviewer for finding our discovery to be of interest. We apologise for any lack of clarity in our original discussion, and fully agree it is important to be nuanced here. We respectfully disagree that this is a descriptive paper. Indeed, our view is that mitophagy is still robustly launched despite the absence of DGAT1-mediated LD biogenesis - but its efficiency is reduced, and we have new evidence to support this (now independently verified by two independent, unrelated laboratories who are collaborating with us). Although the defect in mitochondrial clearance in cultured cells is modest, it is highly consistent. Importantly, we have now pinpointed the level at which the mechanistic impairment lies, and our revised manuscript has rigorously consolidated this mitophagy defect caused by inhibition of DGAT1-dependent LD formation. Ultimately, the relevance of these observations is now authenticated through *in vivo* genetic experimentation and a striking phenotype, the results of which are enclosed in this point-by-point response.

- Should the authors qualify some of their claims as preliminary or speculative, or remove them altogether?

Some claims are still preliminary and while they shouldn't be removed, they should be supported by additional experiments establishing strong causal links between iron depletion, LD formation, ROS, mitophagy and cell viability.

- Would additional experiments be essential to support the claims of the paper? Request additional experiments only where necessary for the paper as it is, and do not ask authors to open new lines of experimentation.

1. Repeat the main findings with another iron chelator in another cell type.

Author response
We thank the reviewer for this suggestion. In terms of using another iron chelator, it is important to highlight that these molecules act *via* distinct mechanisms. The bidentate chelator DFP is distinct from the other commonly used chelator, desferrioxamine (DFO). DFP has prominent effects on mitochondrial iron, whereas this pool seems to be relatively unaffected by DFO (PMID: 17975016, 23628348, 26752519, 32975364). Indeed, Allen *et al*., (PMID: 24176932) previously reported that DFP induces a greater degree of mitophagy than DFO. We predict that DFO would also induce LD biogenesis in many cell types, but the cell biology may very well be different. Nevertheless, we appreciate the reviewer's suggestion and have tried our best to comply within the constraints of the current situation, which have been significant. Accordingly, we have conducted experiments in two distinct cell types: human fibroblast cells and human U-2 OS osteocarcoma cells showing that deferiprone treatment leads to DGAT1-dependent LD accumulation. Representative images and associated quantitation are now included as new **Extended View Figure EV4a-b** and depicted below:

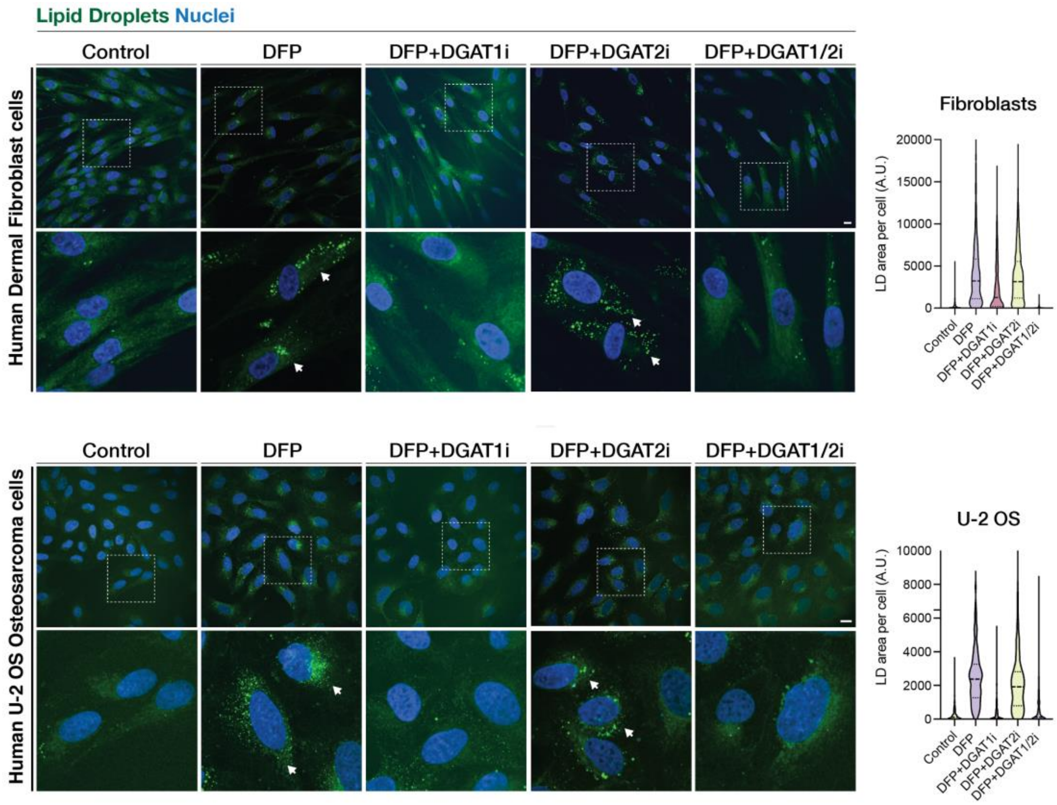

New **Expanded View Figure EV4a-b**

<u>Brief description:</u> iron depletion induces robust LD accumulation across distinct cell types, human ARPE19 cells used in the study and also U-2 OS and fibroblast cells. Across all three distinct cell types, LD biogenesis is blocked upon DGAT1 or combined DGAT1i/2i inhibition.

---

2. LDs can be visualised either by staining with fluorescent dyes (such as BODIPY) or by labelling LD-specific proteins using antibodies (PMID: 23027019). The authors should confirm the BODIPY experiments (Figure 3) with antibodies against LD-specific proteins.

Author response

In the orthogonal validation experiments using distinct cell lines, we also visualised lipid droplets using a distinct dye, LipidTox Deep Red (647) – a widely used reagent in the lipid biology field. We do not plan to include this data, but attach images for the reviewer's interest below:

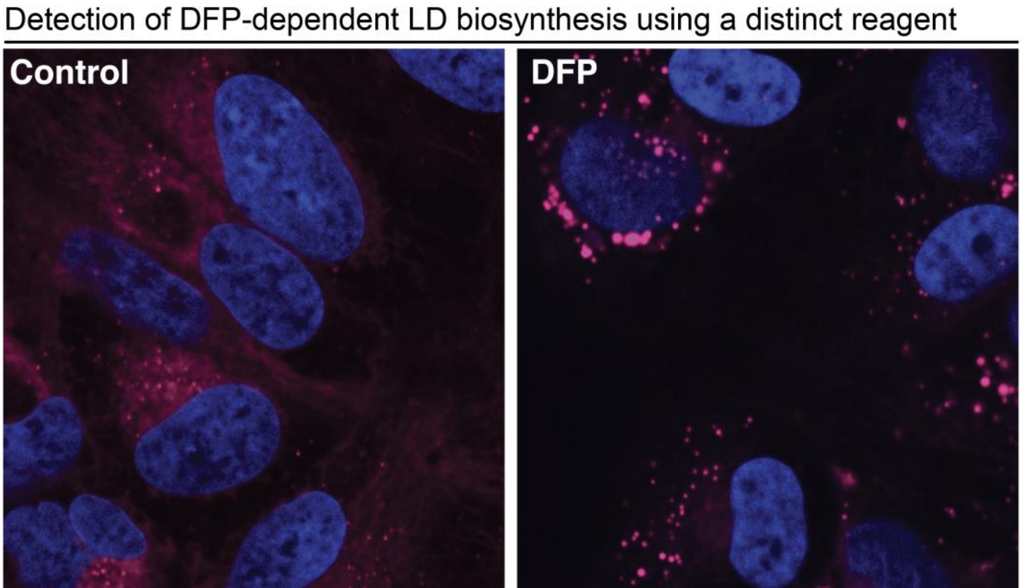

3. The authors should perform a genetic inhibition (CRISPR, siRNA or shRNA) of DGAT1 and DGAT2 to confirm the data obtained with the chemical inhibitors.

- **Author response**
  We thank the reviewer for this suggestion. We have conducted RNAi-mediated depletion of DGAT1 and confirmed that LD biogenesis is suppressed, analogous to the acute inhibitor experiments. These data are shown below and are included in the revised manuscript as **Expanded View Figure EV4c**.

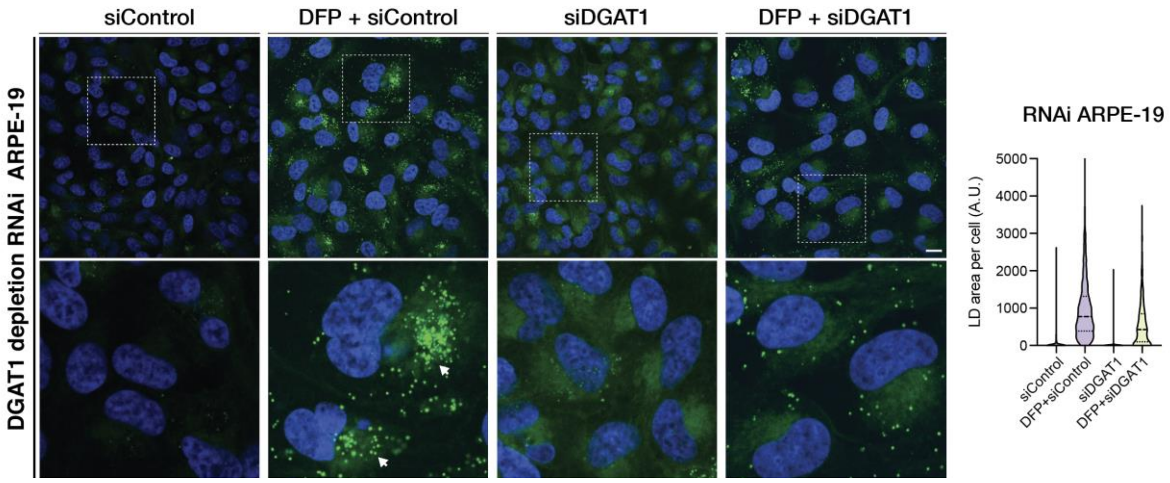

New **Expanded View Figure EV4c**
Brief description: Human ARPE-19 cells were transfected with control small interfering (si) RNA oligonucleotides, or those targeting DGAT1 (siDGAT1) for 72 h and then subjected to

DFP treatment for the last 24 h. Confocal microscopy analysis reveals a robust induction of LD biogenesis in DFP-treated cells with siControl oligonucleotides, but this effect was markedly reduced in the siDGAT1 condition.

- We further verified the effect of impaired LD biogenesis on mitophagy using a distinct cell type and different detection strategy. Using human SH-SY5Y neuroblastoma cells with stable expression of the *mito*-QC reporter, we performed high-content mitophagy quantification by FACS-based analysis. We detected a consistent and statistically significant reduction in DFP-induced mitophagy upon inhibition of LD biogenesis, which is in line with our confocal analyses of ARPE-19 *mito*-QC cells. FACS-based quantitation of mitophagy phenotypes enables high-content analysis but is generally less sensitive than confocal microscopy. Regardless, these data demonstrate a consistent mitophagy defect and are now included in the revised manuscript as an addition to **Figure 6d-e** (also below):

Continued from page 20

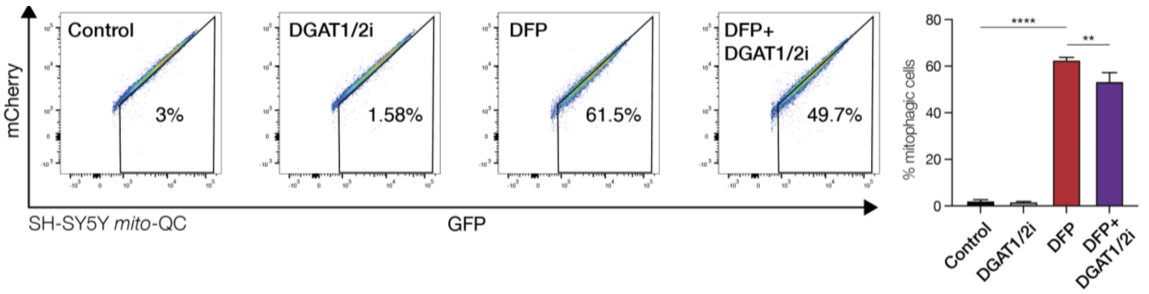

New **Figure 5d-e**: Brief description: FACS-based quantitation showing inhibition of LD biogenesis reduces DFP-induced mitophagy in human SH-SY5Y neuroblastoma cells with stable *mito*-QC expression.

Continued

- Related to the Reviewer's suggestion of a genetic experiment, which was also suggested by Reviewer #2 - we performed a genetic experiment *in vivo*, using depletion of DGAT1 in a distinct mitophagy reporter animal model (*Drosophila matrix*-QC: mCherry-GFP-COXVIII). We thank the reviewer for the suggestion of a genetic experiment, as it has enabled to authenticate the physiological relevance of DGAT1 for mitophagy *in vivo*. Pan-neuronal depletion of DGAT1 (*mdy*) markedly impaired basal mitophagy in brain tissue, accompanied by a significant locomotor phenotype. Taken together, we have used multiple methods to verify the tools used and phenotypes observed in the study and our revised manuscript provides a robust causal and compelling link between lipid homeostasis and physiological mitophagy. We have included these new findings **as new panels in a new main Figure 7** (**data shown below**).

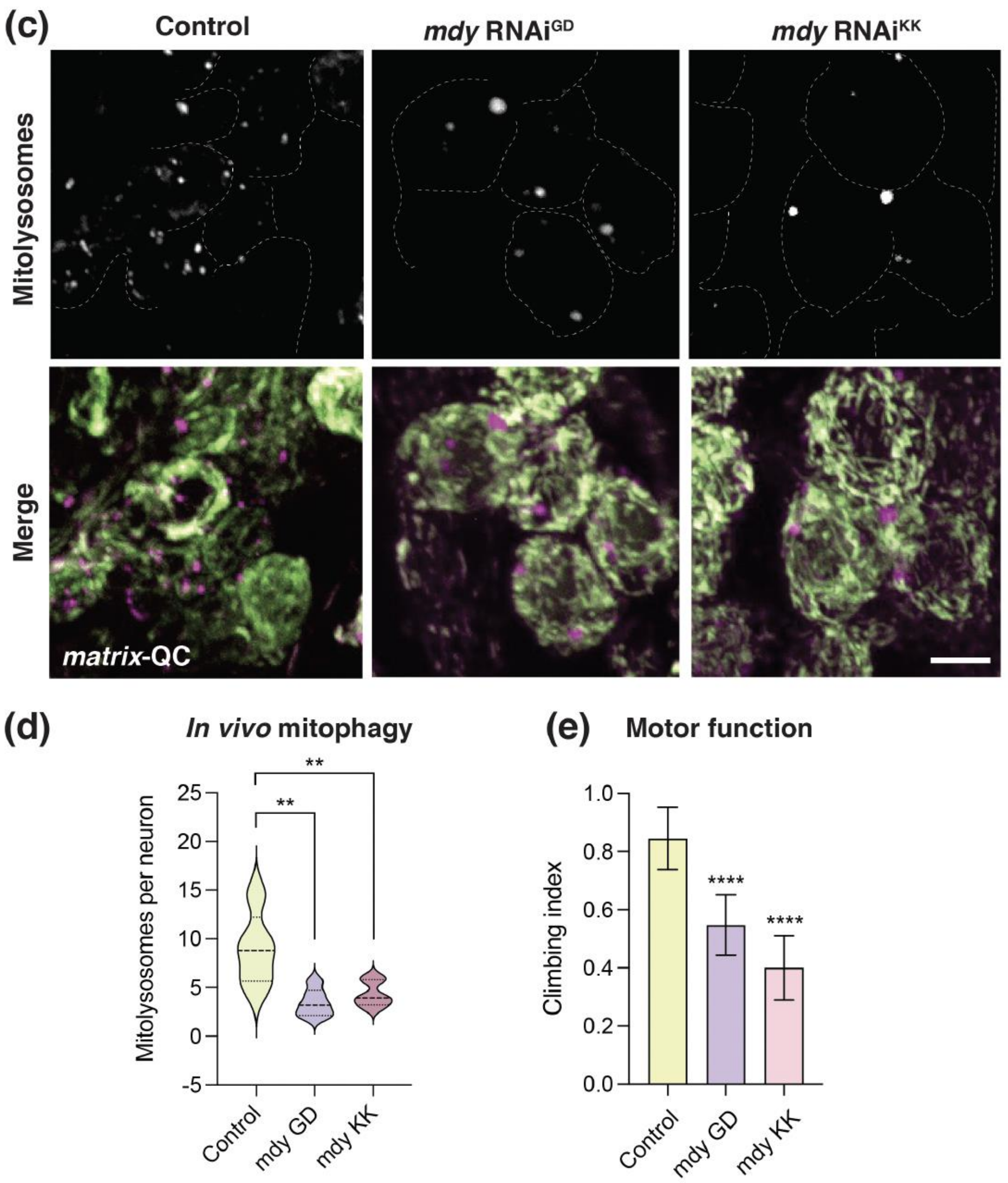

New Figure 7:
Brief description: We assessed the physiological significance of DGAT1 for mitophagy *in vivo* using reporter animals. We accomplished this using pan-neuronal RNAi-mediated depletion of DGAT1 (*mdy*) in *matrix*-QC reporter flies (mCherry-GFP-COXVIII). Quantitation of confocal images from two distinct mutants revealed a robust impairment in mitophagy levels *in vivo* (*n*=6 animals per condition), in addition to motor dysfunction, as revealed by the climbing assay.

"4. Figure 6 is incomplete and disjointed. The authors should establish a firm causal link between LD, ROS, mitophagy and cell integrity. Does DGAT2 inhibition increase DFP-induced mtROS levels?"

Author response

- We apologise for the lack of clarity in our initial submission. In retrospect, we see the reviewer's perspective, and similar feedback was provided by another reviewer. We are fully committed to providing the most precise and reasoned interpretation possible. Accordingly, we have refined our discussion and reduced our focus on mtROS in light of

the compelling physiological phenotype from our genetic experiments. The mtROS data and cell integrity data still stands, these effects are robust and there is an interesting synergy here – but we have restructured the manuscript to provide a more coherent message. As mentioned above, our revised manuscript now contains a rigorous investigation between DGAT1/2 inhibition and mitophagy using several distinct methods. Across all three strategies, we see significant disruption of mitophagy *in vitro* and *in vivo*. Strikingly, mitophagy was most affected *in vivo* and these mutant animals also had a prominent locomotor phenotype. Thus, our manuscript now establishes a clear relationship between lipid homeostasis and physiological mitophagy which has been previously unappreciated. Our present data suggests LDs are not required to sense damaged mitochondria or prime mitochondria for selective autophagy. Rather, LDs offset aberrant lipid accumulation which safeguards lysosomal homeostasis, required for efficient mitophagy (**new Figure 6f-j**). Full resolution of the mechanism was not possible in this manuscript, but we are committed to deconvoluting this relationship further in follow up studies. Deconvoluting how LD biosynthesis protects distinct organelle subpopulations is an enormous task, and one that cannot be achieved presently within our current constraints. Regardless, we are grateful the reviewer for their comments which have helped us refine, streamline and strengthen our manuscript.

- Are the suggested experiments realistic in terms of time and resources? It would help if you could add an estimated cost and time investment for substantial experiments.
Experiments 1-3 should only take 1-2 months (costs around 2000 euros max)
Experiments 4 could take longer and be more costly.
- Are the data and the methods presented in such a way that they can be reproduced?
Yes
- Are the experiments adequately replicated and statistical analysis adequate?
Yes

Minor comments:

- Specific experimental issues that are easily addressable.
Points 1, 2 and 3 above are essential to strengthen the observations.
Point 4 is necessary to establish a mechanistic link between iron chelation, LD formation, ROS, mitophagy and cell viability.
- Are prior studies referenced appropriately?
Overall yes
Discuss the relationship between mitophagy and ROS (lots of existing literature)
- Are the text and figures clear and accurate?
Yes
Minor comment : Figure 3d is missing (yet referred to page 4).

Author response
We will update this in the revised manuscript.

- Do you have suggestions that would help the authors improve the presentation of their data and conclusions?
A cartoon with the suggested mechanism may be useful for discussion.

Author response
We thank the reviewer for this suggestion and agree. Accordingly, we have updated the manuscript with a refined schematic based on new results derived from the additional revision experiments. Please see **new Figure 8**, also below:

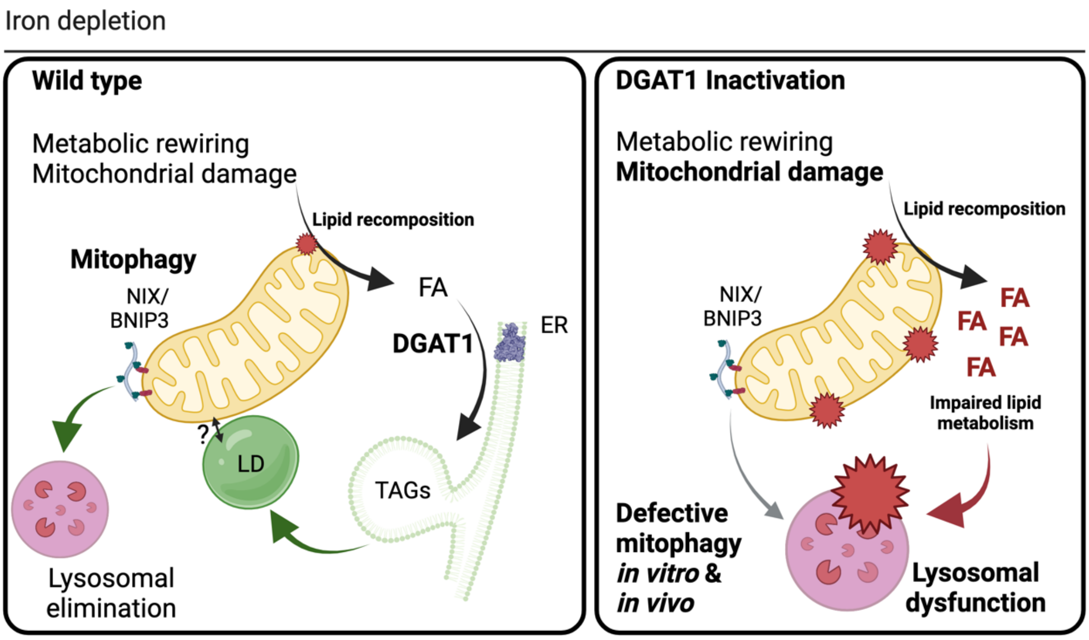

Brief description:
DGAT1 and mitophagy synergise to safeguard cell and tissue integrity
Iron depletion rapidly reshapes the cellular metabolome. DFP treatment alters glucose utilization to promote lipid biosynthesis and TAG storage in lipid droplets *via* DGAT1 activity, upstream of NIX-dependent mitochondrial clearance. Without DGAT1, fatty acids cannot be esterified TAG, compounding lipid dysfunction that impairs lysosomal homeostasis, leading to inefficient mitophagy and promoting cell death. Strikingly, genetic depletion of *DGAT1 in vivo* also impairs basal mitophagy, demonstrating the physiological relevance of our *in vitro* findings.

Reviewer #3 (Significance (Required)):

- Describe the nature and significance of the advance (e.g. conceptual, technical, clinical) for the field.
The link between lipid metabolism and mitophagy is likely to be of interest to the mitophagy field. A link between LDs and non- selective macroautophagy was already established and is mentioned/referenced in the manuscript.
- Place the work in the context of the existing literature (provide references, where appropriate).
The mitophagy field is largely studied at the moment so this is a timely study

- State what audience might be interested in and influenced by the reported findings.
Link between lipid metabolism and mitophagy
- Define your field of expertise with a few keywords to help the authors contextualize your point of view. Indicate if there are any parts of the paper that you do not have sufficient expertise to evaluate.
Expertise in mitophagy
Little expertise in lipid biology

Author response
We thank the Reviewer for their time and assistance in helping us refine our submission. These comments have greatly benefited our study. We greatly appreciate their recognition that our study is timely and of interest.

Dear Thomas,

Thank you for submitting your revised manuscript (EMBOJ-2021-109390R) to The EMBO Journal. Your amended study was sent back to the three referees for re-evaluation, and we have received comments from all of them, which I enclose below. As you will see, the referees stated that the issues raised have been adequately addressed and they are broadly now in favour of publication, pending a minor revision.

Thus, we are pleased to inform you that your manuscript has been accepted in principle for publication in The EMBO Journal.

Please consider the remaining points of referee #1 carefully, and address these by introducing caveats in the discussion of the results where appropriate.

In addition, we need you to take care of a number of minor issues related to formatting of the manuscript text as detailed below, which should be addressed at re-submission.

Please contact me at any time if you have additional questions related to below points.

Thank you for giving us the chance to consider your manuscript for The EMBO Journal. I look forward to your final revision.

with
Best regards,

Daniel

Daniel Klimmeck PhD
Senior Editor
The EMBO Journal

Formatting changes required for the revised version of the manuscript:

>> Adjust the title of the 'Competing Interests' section to 'Disclosure and Competing Interests Statement'.

>> Move the Materials and Methods after the Discussion section.

Further information is available in our Guide For Authors: https://www.embopress.org/page/journal/14602075/authorguide

We realize that it is difficult to revise to a specific deadline. In the interest of protecting the conceptual advance provided by the work, we recommend a revision within 3 months (9th May 2022). Please discuss the revision progress ahead of this time with the editor if you require more time to complete the revisions. Use the link below to submit your revision:

https://emboj.msubmit.net/cgi-bin/main.plex

Referee #1:

The authors successfully addressed most of the Review Commons comments and changed the conclusions based on the new data obtained. The results in flies significantly added to the physiological relevance of the pathway defined in ARPE19. I just have minor comments about some text and conclusions that need to be revised to be consistent with the new data and conclusions:

-Authors write "These findings suggest DFP impairs the import and oxidation of free fatty

acids (FFAs) to mitochondria". As no beta oxidation could be measured, the findings shown do not support this statement. Along the text, it should be clarified that lipid droplets can not only as a result of defective mitochondrial beta oxidation to increase FFA availability, but also by the activation of biosynthetic pathways (lipogenesis). In this regard, the data show here and the importance of DGAT1 strongly supports that there is an early activation of lipogenesis by DFP that precedes iron-chelation induced mitochondrial dysfunction.

-A comment could be added that FFA themselves can not only damage lysosomes, but also mitochondria, which can explain increased NIX recrutiment upon DGAT inhibition. Again, an excessive increase in intracellular FFA can explain mitochondrial dysfunction and increased ROS production, rather than mitochondrial dysfunction being upstream to cause FFA accumulation (see literature of Paolo Bernardi and others on the role of FFA on mitochondrial depolarization and pore opening).

-Authors should consider using lyostracker (ratiometric dye measuring pH). It is a tricky measurement, as the time of incubation and proper washes are critical for reliable measurements. But it would be really nice to have good measures of lysosomal pH. It is a key conclusion of the revised manuscript.

Referee #2:

The authors have done a great job addressing the comments resulting in a strengthened manuscript. The response to reviewers was very detailed and well explained with good justifications. I don't have any further comments to add. Congratulations on a very interesting discovery!

Referee #3:

In my opinoin, the manuscript is now ready for publication in the EMBO J.

- The authors have done their best to confirme their findings with the tools and methods available.
- The depletion of DGAT1 in the mitophagy flies is key to confirm the findings in vivo.
- The authors have restructured the masnucript so that the link between iron depletion, LD biogenesis and mitophagy is clearer (the cratoon - New Figure 8 - is helpful in getting the message across).
- The mechanism remains to be fully undesrtood, but we all know that fully understanding a mechanism can take a long time, and it would be a shame to delay this publication further as I believe it will be of great interest for the EMBO readership.

Review Commons article RC-2021-00870R (Accepted-in-principle)
Long *et al*.
Response to Reviewers comments II.

Author response

We thank the Editor and the Reviewers for their excellent and helpful feedback. We agree with Reviewer 1 and have incorporated their suggestions as requested.

Many thanks once again to everyone for a very constructive review process.

On behalf of the authors,
Tom McWilliams

Reviewer comments

Referee #1:

"The authors successfully addressed most of the Review Commons comments and changed the conclusions based on the new data obtained. The results in flies significantly added to the physiological relevance of the pathway defined in ARPE19. I just have minor comments about some text and conclusions that need to be revised to be consistent with the new data and conclusions:

-Authors write "These findings suggest DFP impairs the import and oxidation of free fatty acids (FFAs) to mitochondria". As no beta oxidation could be measured, the findings shown do not support this statement. Along the text, it should be clarified that lipid droplets can not only as a result of defective mitochondrial beta oxidation to increase FFA availability, but also by the activation of biosynthetic pathways (lipogenesis). In this regard, the data show here and the importance of DGAT1 strongly supports that there is an early activation of lipogenesis by DFP that precedes iron-chelation induced mitochondrial dysfunction.

-A comment could be added that FFA themselves can not only damage lysosomes, but also mitochondria, which can explain increased NIX recrutiment upon DGAT inhibition. Again, an excessive increase in intracellular FFA can explain mitochondrial dysfunction and increased ROS production, rather than mitochondrial dysfunction being upstream to cause FFA accumulation (see literature of Paolo Bernardi and others on the role of FFA on mitochondrial depolarization and pore opening).

-Authors should consider using lyostracker (ratiometric dye measuring pH). It is a tricky measurement, as the time of incubation and proper washes are critical for reliable measurements. But it would be really nice to have good measures of lysosomal pH. It is a key conclusion of the revised manuscript."

Author response

We thank the Reviewer for their time and constructive feedback; their input has improved and strengthened our manuscript. We agree with their comments and have incorporated these helpful clarifications and associated citations into the revised submission.

We have updated the manuscript to include the following amendments as suggested:

Lines 128-129, Page number 5 of the "merged PDF", highlighted in yellow
"*These findings suggest DFP treatment may impair fatty acid metabolism with a corresponding reciprocal increase in TAG biosynthesis*.

Lines 398-400, Page number 11 of the "merged PDF", highlighted in yellow
"Ultimately, iron depletion reshaped the metabolome by eight hours of treatment, with the early induction of de novo lipogenesis and DGAT1 activity required to esterify fatty acids to TAG for storage within LDs."

Line 438-440, Page number 12 of the "merged PDF", highlighted in yellow
"Aside from lysosomal homeostasis, excess NEFAs also induce mitochondrial dysfunction, which might explain the increased NIX recruitment and mtROS levels observed upon DGAT inhibition (Penzo et al. 2002)."

We thank the Reviewer once again for their insightful contributions to our manuscript.

Referee #2:

"The authors have done a great job addressing the comments resulting in a strengthened manuscript. The response to reviewers was very detailed and well explained with good justifications. I don't have any further comments to add. Congratulations on a very interesting discovery!"

Author response

We thank the Reviewer for their time, positive comments and helpful contributions.

Referee #3:

"In my opinoin, the manuscript is now ready for publication in the EMBO J.

- The authors have done their best to confirme their findings with the tools and methods available.
- The depletion of DGAT1 in the mitophagy flies is key to confirm the findings in vivo.
- The authors have restructured the masnucript so that the link between iron depletion,biogenesis and mitophagy is clearer (the cratoon - New Figure 8 - is helpful in getting the message across).
- The mechanism remains to be fully undesrtood, but we all know that fully understanding a mechanism can take a long time, and it would be a shame to delay this publication further as I believe it will be of great interest for the EMBO readership."

Author response

We thank the Reviewer for their time, constructive feedback, and helpful insights. We greatly appreciate their understanding.

2nd Revision - Editorial Decision 25th Feb 2022

Dear Dr McWilliams,

Thank you for submitting the revised version of your manuscript. I have now evaluated your amended manuscript and concluded that the remaining minor concerns have been sufficiently addressed.

Thus, I am pleased to inform you that your manuscript has been accepted for publication in the EMBO Journal.

Please note that it is EMBO Journal policy for the transcript of the editorial process (containing referee reports and your response letter) to be published as an online supplement to each paper.

Also, in case you might NOT want the transparent process file published at all, you will also need to inform us via email immediately. More information is available here:
https://www.embopress.org/page/journal/14602075/authorguide#transparentprocess

----

Please note that in order to be able to start the production process, our publisher will need and contact you regarding the following forms:

- PAGE CHARGE AUTHORISATION (For Articles and Resources)
http://onlinelibrary.wiley.com/journal/10.1002/(ISSN)1460-2075/homepage/tej_apc.pdf

- LICENCE TO PUBLISH (for non-Open Access)

Your article cannot be published until the publisher has received the appropriate signed license agreement. Once your article has been received by Wiley for production you will receive an email from Wiley's Author Services system, which will ask you to log in and will present them with the appropriate license for completion.

- LICENCE TO PUBLISH for OPEN ACCESS papers

Authors of accepted peer-reviewed original research articles may choose to pay a fee in order for their published article to be made freely accessible to all online immediately upon publication. The EMBO Open fee is fixed at $5,200 (+ VAT where applicable).

We offer two licenses for Open Access papers, CC-BY and CC-BY-NC-ND.
For more information on these licenses, please visit:
http://creativecommons.org/licenses/by-nc-nd/3.0/deed.en_US

- PAYMENT FOR OPEN ACCESS papers

You also need to complete our payment system for Open Access articles. Please follow this link and select EMBO Journal from the drop down list and then complete the payment process: https://authorservices.wiley.com/bauthor/onlineopen_order.asp

Notably, please be reminded that under the DEAL agreement of European scientific institutions with our publisher Wiley, you could be eligible for free publication of your article in the open access format. Please contact either the administration at your institution or Wiley (embojournal@wiley.com) to clarify further questions.

Should you be planning a Press Release on your article, please get in contact with embojournal@wiley.com as early as possible, in order to coordinate publication and release dates.

On a different note, I would like to alert you that EMBO Press is currently developing a new format for a video-synopsis of work published with us, which essentially is a short, author-generated film explaining the core findings in hand drawings, and, as we believe, can be very useful to increase visibility of the work. This has proven to offer a nice opportunity for exposure i.p. for the first author(s) of the study. Please see the following link for representative examples and their integration into the article web page:
https://www.embopress.org/video_synopses
https://www.embopress.org/doi/full/10.15252/embj.2019103932

Please let me know, should you be interested to engage in commissioning a similar video synopsis for your work. According operation instructions are available and intuitive.

If you have any questions, please do not hesitate to call or email the Editorial Office.

Thank you for this contribution to The EMBO Journal and congratulations on a successful publication!

Please consider us again in the future for your most exciting work.

Kind regards,

Daniel Klimmeck

Daniel Klimmeck, PhD
Senior Editor
The EMBO Journal
EMBO
Postfach 1022-40
Meyerhofstrasse 1
D-69117 Heidelberg
contact@embojournal.org
Submit at: http://emboj.msubmit.net

EMBO PRESS

YOU MUST COMPLETE ALL CELLS WITH A PINK BACKGROUND ↓

PLEASE NOTE THAT THIS CHECKLIST WILL BE PUBLISHED ALONGSIDE YOUR PAPER

Corresponding Author Name: Thomas G. McWilliams

Journal Submitted to: The EMBO Journal
Manuscript Number: EMBOJ-2021-109390R

Reporting Checklist For Life Sciences Articles (Rev. June 2017)

This checklist is used to ensure good reporting standards and to improve the reproducibility of published results. These guidelines are consistent with the Principles and Guidelines for Reporting Preclinical Research issued by the NIH in 2014. Please follow the journal's authorship guidelines in preparing your manuscript.

A- Figures
1. Data
The data shown in figures should satisfy the following conditions:

- ➔ the data were obtained and processed according to the field's best practice and are presented to reflect the results of the experiments in an accurate and unbiased manner.
- ➔ figure panels include only data points, measurements or observations that can be compared to each other in a scientifically meaningful way.
- ➔ graphs include clearly labeled error bars for independent experiments and sample sizes. Unless justified, error bars should not be shown for technical replicates.
- ➔ if n< 5, the individual data points from each experiment should be plotted and any statistical test employed should be justified
- ➔ Source Data should be included to report the data underlying graphs. Please follow the guidelines set out in the author ship guidelines on Data Presentation.

2. Captions

Each figure caption should contain the following information, for each panel where they are relevant:

- ➔ a specification of the experimental system investigated (eg cell line, species name).
- ➔ the assay(s) and method(s) used to carry out the reported observations and measurements
- ➔ an explicit mention of the biological and chemical entity(ies) that are being measured.
- ➔ an explicit mention of the biological and chemical entity(ies) that are altered/varied/perturbed in a controlled manner.

- ➔ the exact sample size (n) for each experimental group/condition, given as a number, not a range;
- ➔ a description of the sample collection allowing the reader to understand whether the samples represent technical or biological replicates (including how many animals, litters, cultures, etc.).
- ➔ a statement of how many times the experiment shown was independently replicated in the laboratory.
- ➔ definitions of statistical methods and measures:
  - common tests, such as t-test (please specify whether paired vs. unpaired), simple $\chi 2$ tests, Wilcoxon and Mann-Whitney tests, can be unambiguously identified by name only, but more complex techniques should be described in the methods section;
  - are tests one-sided or two-sided?
  - are there adjustments for multiple comparisons?
  - exact statistical test results, e.g., P values = x but not P values < x;
  - definition of 'center values' as median or average;
  - definition of error bars as s.d. or s.e.m.

Any descriptions too long for the figure legend should be included in the methods section and/or with the source data.

In the pink boxes below, please ensure that the answers to the following questions are reported in the manuscript itself. Every question should be answered. If the question is not relevant to your research, please write NA (non applicable). We encourage you to include a specific subsection in the methods section for statistics, reagents, animal models and human subjects.

USEFUL LINKS FOR COMPLETING THIS FORM

http://www.antibodypedia.com
http://1degreebio.org
http://www.equator-network.org/reporting-guidelines/improving-bioscience-research-repor
http://grants.nih.gov/grants/olaw/olaw.htm
http://www.mrc.ac.uk/Ourresearch/Ethicsresearchguidance/Useofanimals/index.htm
http://ClinicalTrials.gov
http://www.consort-statement.org
http://www.consort-statement.org/checklists/view/32-consort/66-title
http://www.equator-network.org/reporting-guidelines/reporting-recommendations-for-tumo
http://datadryad.org
http://figshare.com
http://www.ncbi.nlm.nih.gov/gap
http://www.ebi.ac.uk/ega
http://biomodels.net/
http://biomodels.net/miriam/
http://jjj.biochem.sun.ac.za
https://osp.od.nih.gov/biosafety-biosecurity-and-emerging-biotechnology/
http://www.selectagents.gov/

B- Statistics and general methods

Please fill out these boxes ↓ (Do not worry if you cannot see all your text once you press return)

1.a. How was the sample size chosen to ensure adequate power to detect a pre-specified effect size?	No formal sample size calculation was performed but sample sizes were guided by previous experience from extensive phenotyping. The sample size depicted in biochemical assays reflects the number of independently measured experiments (n). The number of technical and independent biological experiment is stated in each figure legend.
1.b. For animal studies, include a statement about sample size estimate even if no statistical methods were used.	Due to the lack of specific regulation of use of Drosophila, sample size estimates were determined in accordance with previous phenotyping experience rather than power calculations. Number of flies used in each experiment is indicated in the figure legends and materials and methods.
2. Describe inclusion/exclusion criteria if samples or animals were excluded from the analysis. Were the criteria pre-established?	No animals were specifically included or excluded in any of the anlaysis performed in this work.
3. Were any steps taken to minimize the effects of subjective bias when allocating animals/samples to treatment (e.g. randomization procedure)? If yes, please describe.	No
For animal studies, include a statement about randomization even if no randomization was used.	For all the analyses, samples were collected and processed simultaneously and therefore no randomization was used in the animal studies.
4.a. Were any steps taken to minimize the effects of subjective bias during group allocation or/and when assessing results (e.g. blinding of the investigator)? If yes please describe.	Yes. Mitophagy and motor function assays in Drosophila were analysed in a blinded manner, for motor function assays - groups of flies used were blinded by a different investigator than the one performing the assay.
4.b. For animal studies, include a statement about blinding even if no blinding was done	Mitophagy images in Drosophila were blinded prior to analysis. For motor fuction assays, the groups of flies used were blinded by a different investigator than the one performing the assay.
5. For every figure, are statistical tests justified as appropriate?	Yes
Do the data meet the assumptions of the tests (e.g., normal distribution)? Describe any methods used to assess it.	Yes. For climbing assays, the data are not normally distributed so a Kruskal-Wallis nonparametric test with Dunn's post-hoc correction for multiple comparisons was used. For analysis of mitolysosomes, the data passed normality test so statistical significance was calculated by one-way ANOVA with Šidák's post-test correction for multiple samples
Is there an estimate of variation within each group of data?	No

Is the variance similar between the groups that are being statistically compared?	Yes

C- Reagents

6. To show that antibodies were profiled for use in the system under study (assay and species), provide a citation, catalog number and/or clone number, supplementary information or reference to an antibody validation profile. e.g., Antibodypedia (see link list at top right), 1DegreeBio (see link list at top right).	All primary antibodies have been validated for specificity in human cell or tissue specimens, by the manufacturer. Each primary antibody has featured in several publications as listed on the manufacturers website and the CiteAb database. Complete information regarding RRID numbers, catalog numbers, clones and dilutions can be found in the materials and methods.
7. Identify the source of cell lines and report if they were recently authenticated (e.g., by STR profiling) and tested for mycoplasma contamination.	Cells were tested negative for Mycoplasma. Cell lines were puchased from ATCC and displayed expected cell morphologies.

* for all hyperlinks, please see the table at the top right of the document

D- Animal Models

8. Report species, strain, gender, age of animals and genetic modification status where applicable. Please detail housing and husbandry conditions and the source of animals.	All the details regarding Drosopohila melanogaster housing, husbandry, source of strains (RRID numbers), gender and conditions for each experiment are explain in the material and methods section of this manuscript. For viablity and mitophagy assays a mix of males and females were used to perform the experiment. For motor function assay, 2 days old adult males were used.
9. For experiments involving live vertebrates, include a statement of compliance with ethical regulations and identify the committee(s) approving the experiments.	N/A
10. We recommend consulting the ARRIVE guidelines (see link list at top right) (PLoS Biol. 8(6), e1000412, 2010) to ensure that other relevant aspects of animal studies are adequately reported. See author guidelines, under 'Reporting Guidelines'. See also: NIH (see link list at top right) and MRC (see link list at top right) recommendations. Please confirm compliance.	N/A

E- Human Subjects

11. Identify the committee(s) approving the study protocol.	N/A
12. Include a statement confirming that informed consent was obtained from all subjects and that the experiments conformed to the principles set out in the WMA Declaration of Helsinki and the Department of Health and Human Services Belmont Report.	N/A
13. For publication of patient photos, include a statement confirming that consent to publish was obtained.	N/A
14. Report any restrictions on the availability (and/or on the use) of human data or samples.	N/A
15. Report the clinical trial registration number (at ClinicalTrials.gov or equivalent), where applicable.	N/A
16. For phase II and III randomized controlled trials, please refer to the CONSORT flow diagram (see link list at top right) and submit the CONSORT checklist (see link list at top right) with your submission. See author guidelines, under 'Reporting Guidelines'. Please confirm you have submitted this list.	N/A
17. For tumor marker prognostic studies, we recommend that you follow the REMARK reporting guidelines (see link list at top right). See author guidelines, under 'Reporting Guidelines'. Please confirm you have followed these guidelines.	N/A

F- Data Accessibility

18: Provide a "Data Availability" section at the end of the Materials & Methods, listing the accession codes for data generated in this study and deposited in a public database (e.g. RNA-Seq data: Gene Expression Omnibus GSE39462, Proteomics data: PRIDE PXD000208 etc.) Please refer to our author guidelines for 'Data Deposition'.<br><br>Data deposition in a public repository is mandatory for:<br>a. Protein, DNA and RNA sequences<br>b. Macromolecular structures<br>c. Crystallographic data for small molecules<br>d. Functional genomics data<br>e. Proteomics and molecular interactions	N/A
19. Deposition is strongly recommended for any datasets that are central and integral to the study; please consider the journal's data policy. If no structured public repository exists for a given data type, we encourage the provision of datasets in the manuscript as a Supplementary Document (see author guidelines under 'Expanded View' or in unstructured repositories such as Dryad (see link list at top right) or Figshare (see link list at top right).	N/A
20. Access to human clinical and genomic datasets should be provided with as few restrictions as possible while respecting ethical obligations to the patients and relevant medical and legal issues. If practically possible and compatible with the individual consent agreement used in the study, such data should be deposited in one of the major public access-controlled repositories such as dbGAP (see link list at top right) or EGA (see link list at top right).	N/A
21. Computational models that are central and integral to a study should be shared without restrictions and provided in a machine-readable form. The relevant accession numbers or links should be provided. When possible, standardized format (SBML, CellML) should be used instead of scripts (e.g. MATLAB). Authors are strongly encouraged to follow the MIRIAM guidelines (see link list at top right) and deposit their model in a public database such as Biomodels (see link list at top right) or JWS Online (see link list at top right). If computer source code is provided with the paper, it should be deposited in a public repository or included in supplementary information.	N/A

G- Dual use research of concern

22. Could your study fall under dual use research restrictions? Please check biosecurity documents (see link list at top right) and list of select agents and toxins (APHIS/CDC) (see link list at top right). According to our biosecurity guidelines, provide a statement only if it could.	N/A
